# The SARS-CoV-2 receptor and other key components of the Renin-Angiotensin-Aldosterone System related to COVID-19 are expressed in enterocytes in larval zebrafish

**DOI:** 10.1242/bio.058172

**Published:** 2021-03-23

**Authors:** John H. Postlethwait, Michelle S. Massaquoi, Dylan R. Farnsworth, Yi-Lin Yan, Karen Guillemin, Adam C. Miller

**Affiliations:** 1 Institute of Neuroscience, University of Oregon, Eugene, OR 97403, USA; 2 Institute of Molecular Biology, University of Oregon, Eugene, OR 97403, USA

**Keywords:** scRNA-seq, Apelin, COVID-19, *Danio rerio*, Conserved synteny, Genome duplication

## Abstract

People with underlying conditions, including hypertension, obesity, and diabetes, are especially susceptible to negative outcomes after infection with coronavirus SARS-CoV-2, which causes COVID-19. Hypertension and respiratory inflammation are exacerbated by the Renin-Angiotensin-Aldosterone System (RAAS), which normally protects from rapidly dropping blood pressure via Angiotensin II (Ang II) produced by the enzyme Ace. The Ace paralog Ace2 degrades Ang II, counteracting its chronic effects, and serves as the SARS-CoV-2 receptor. *Ace*, the coronavirus, and COVID-19 comorbidities all regulate *Ace2*, but we do not yet understand how. To exploit zebrafish (*Danio rerio*) to help understand the relationship of the RAAS to COVID-19, we must identify zebrafish orthologs and co-orthologs of human RAAS genes and understand their expression patterns. To achieve these goals, we conducted genomic and phylogenetic analyses and investigated single cell transcriptomes. Results showed that most human RAAS genes have one or more zebrafish orthologs or co-orthologs. Results identified a specific type of enterocyte as the specific site of expression of zebrafish orthologs of key RAAS components, including Ace, Ace2, Slc6a19 (SARS-CoV-2 co-receptor), and the Angiotensin-related peptide cleaving enzymes Anpep (receptor for the common cold coronavirus HCoV-229E), and Dpp4 (receptor for the Middle East Respiratory Syndrome virus, MERS-CoV). Results identified specific vascular cell subtypes expressing Ang II receptors, *apelin*, and *apelin receptor* genes. These results identify genes and cell types to exploit zebrafish as a disease model for understanding mechanisms of COVID-19.

## Abstract (precis)

**Summary:** Genomic analyses identify zebrafish orthologs of the Renin-Angiotensin-Aldosterone System that contribute to COVID-19 comorbidities and single-cell transcriptomics shows that they act in a specialized intestinal cell type.

## INTRODUCTION

Coronaviruses have led to three epidemics in the 21st century: Severe Acute Respiratory syndrome caused by SARS-CoV (WHO, 2004), Middle East Respiratory Syndrome, caused by MERS-CoV (WHO, 2016), and Corona Virus Disease 2019 (COVID-19) (Lu et al., 2020) caused by SARS-CoV-2. COVID-19 symptoms include pneumonia, fever, persistent cough, lung inflammation, diarrhea, acute kidney injury, and death (Cheng et al., 2020; Meo et al., 2020). Remarkably, about 20% of SARS-CoV-19 infections are asymptomatic (Bai et al., 2020; Streeck et al., 2020), promoting surreptitious dissemination. COVID-19 risk factors include sex, race, and age (Du et al., 2020; Jin et al., 2020; Price-Haywood et al., 2020; Richardson et al., 2020). Risk factors correlate with certain chronic health problems: among nearly 6000 patients hospitalized with COVID-19 in New York, 94% had a pre-existing health issue, frequently hypertension (57%), obesity (42%), and diabetes (34%) (Richardson et al., 2020). We do not yet fully understand how these underlying conditions exacerbate COVID-19, but they are related to the Renin-Angiotensin-Aldosterone System (RAAS), which modulates blood volume and blood pressure (Cabandugama et al., 2017; Fyhrquist and Saijonmaa, 2008). The RAAS is linked to COVID-19 in two important ways. First, COVID-19 comorbidities mimic a chronically over-active RAAS and second, the receptor for SARS-CoV-2 (Ace2) is a key RAAS component.

Homeostasis of the cardiovascular and respiratory systems that are impacted by COVID-19 depend on the RAAS hormone Angiotensin II (Ang II). This peptide promotes vasoconstriction, salt and water retention, inflammation, and production of reactive oxygen species (ROS) (Fig. S1). These features are helpful to counteract a sudden drop in blood pressure or infection but are harmful when chronic (Fyhrquist and Saijonmaa, 2008). Vasoconstriction and inflammation also contribute to COVID-19 comorbidities, including hypertension, diabetes, cardiovascular disease, and cerebrovascular disease (Wang et al., 2020). Ang II forms when Ace cleaves two amino acids from Ang I, a peptide produced when Renin digests Angiotensinogen (Fyhrquist and Saijonmaa, 2008). Ace inhibitor drugs and Ang II receptor blockers decrease Ang II effects and thus dampen hypertension and other COVID-19 comorbidities (Rice et al., 2004; Messerli et al., 2018; Natesh et al., 2004; Sommerstein et al., 2020). Furthermore, people taking Ace inhibitors and Ang II receptor blockers at home had reduced risks of having COVID-19 disease after adjusting for other factors and did not show increased risk of ICU care (Hippisley-Cox et al., 2020).

COVID-19 also links to the RAAS because the spike protein on the surface of SARS-CoV-2 binds near the active site of Ace2, a cell-surface monocarboxy peptidase, allowing infection (Gallagher and Buchmeier, 2001; Hoffmann et al., 2020; Millet and Whittaker, 2015; Simmons et al., 2013; Walls et al., 2020; Yan et al., 2020a). The normal role of Ace2 is to metabolize Ang II to Ang1-7, thus decreasing hypertension and retention of water and salt. The binding of SARS-CoV to Ace2, however, inhibits Ace2 activity (Kuba et al., 2005). Mice lacking *Angiotensinogen* or *Ang II receptor* genes show decreased obesity, insulin resistance, and hypertension (Massiera et al., 2001; Yvan-Charvet et al., 2005), supporting the notion that RAAS activation contributes to COVID-19 comorbidities. Ace2 can also cleave Apelin (Apln) peptides, which cause vasodilation, increased heart muscle contractility, angiogenesis, fluid homeostasis, and regulation of energy metabolism, thus, countering effects of Ang II (De Mota et al., 2004; Dray et al., 2008; Kasai et al., 2004; Szokodi et al., 2002; Yang et al., 2017). Apln is a positive regulator of Ace2, so decreasing Apln downregulates Ace2 expression, thereby decreasing the number of virus receptors, but increasing Ang II levels, prolonging harmful effects on comorbidities (Sato et al., 2013).

The molecular genetic bases of complex diseases like COVID-19 are often illuminated by investigations in model organisms (Wangler et al., 2017). Among vertebrate disease models, zebrafish (*Danio rerio*) is a workhorse for understanding the development and physiology of organs related to COVID-19, including heart and vasculature, kidney, liver, and others (e.g. Bournele and Beis, 2016; Goessling and Sadler, 2015; Morales and Wingert, 2017). Zebrafish can even contribute to understanding communicable human diseases (Bouz and Al Hasawi, 2018; Clay et al., 2007; Sullivan et al., 2017). Zebrafish accelerates research into human disease mechanisms because embryos and larvae develop in a dish and are optically favorable, allowing visualization of transgenic fluorescent markers in functioning organs in living animals (e.g. Wiles et al., 2016). For example, transgenic zebrafish expressing GFP in TNFA-positive cells and mCherry in neutrophils (Lam et al., 2012; Marjoram et al., 2015) can make visible components of the ‘cytokine storm’ that can lead to death of adult COVID-19 patients and to the pediatric multi-system inflammatory syndrome that affects some SARS-CoV-2-infected children (New York Department of Health, 2020; Ye et al., 2020; Zhang et al., 2020). Zebrafish have vertebrate-specific organs and organ systems that mimic our own, they develop and function using similar regulatory mechanisms, and they are sensitive to SARS-CoV-2 exposure (Kraus et al., 2020 preprint). In addition, small size makes zebrafish suitable for screens of therapeutic molecules at a scale not possible in mammals (Lam and Peterson, 2019).

To evaluate zebrafish as a model for mechanistic insights into the links between COVID-19 comorbidities and the RAAS, we first identified the orthologs and co-orthologs of human RAAS- and apelin-related genes in zebrafish. Zebrafish orthologs of human genes are sometimes obscured by genome duplication events [two rounds in stem vertebrates (Dehal and Boore, 2005; Simakov et al., 2020) and a third round in stem teleosts (Amores et al., 1998; Jaillon et al., 2004; Postlethwait et al., 1999; Taylor et al., 2001)]. Second, we explored the expression of RAAS-related genes in single-cell transcriptomic (scRNA-seq) experiments from zebrafish embryos and larvae, providing the first organism-wide view of RAAS gene expression at the level of individual cell types. Analyses showed first, that zebrafish and humans share most genomic features of the RAAS and apelin systems while identifying previously unrecognized orthologs and co-orthologs and clarifying relationships; and second, that many RAAS components are expressed by a specific type of enterocyte, making them the focal cell type for the RAAS that merits further exploration. A similar cell type in humans allows SARS-CoV-2 infection, the production of infectious virus, and likely some COVID-19 pathologies (Stanifer et al., 2020). These studies support zebrafish as a model for investigating the relationship of the RAAS to COVID-19 pathologies.

## RESULTS

Analyses described below follow an outline of RAAS/Apln signaling in Fig. S1 (see also Fyhrquist and Saijonmaa, 2008; Zhang et al., 2018).

### Angiotensinogen

**Fig. 1.**
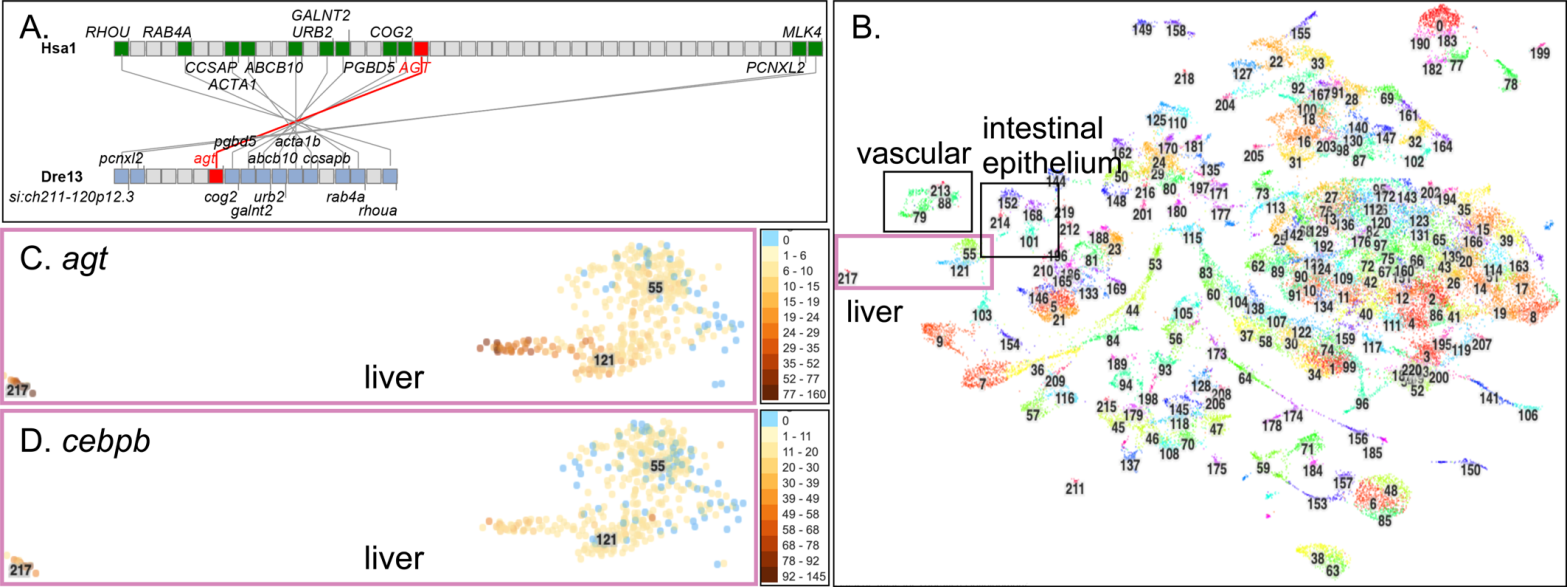
**Genomics and expression of *AGT*.** (A) The section of *H. sapiens* chromosome 1 (Hsa1) that contains *AGT* is conserved with the segment of *D. rerio* chromosome 13 (Dre13) that contains *agt*. (B) The 220 clusters from the zebrafish scRNA-seq Atlas (Farnsworth et al., 2020). Boxes indicate the liver, vascular endothelium, and intestinal epithelium clusters. (C) Cells in hepatocyte clusters c217, c121, and c121 express *agt.* Each dot represents a cell. Color intensity indicates expression level according to the scale at the right [the number of Unique Molecular Identifiers (UMIs; unique reads) in each individual cell that mapped to the gene of interest]. Blue cells are not expressing. (D) Expression of the Agt-regulator *cebpb* in larval liver cells.

Angiotensinogen (Agt) is the protein precursor of Ang II (Fig. S1.1). *AGT* (ENSGT00890000139531) on human (*Homo sapiens*) chromosome 1 (Hsa1) and *agt* (ENSDARG00000016412) on zebrafish (*D. rerio*) chromosome 13 (Dre13) conserve syntenies (Fig. 1A). Phylogenetic analysis confirmed that zebrafish has a single ortholog of human *AGT* (Fig. S2A). Mammalian and zebrafish Agt proteins share their overall three-dimensional structure (Lu et al., 2016) and are both strongly expressed in adult liver (Cheng et al., 2006; Fagerberg et al., 2014). Analysis of our zebrafish scRNA-seq atlas (here called the ‘Atlas’), which combines cells from 1 and 2 day post-fertilization (dpf) embryos and from 5 dpf larvae (Farnsworth et al., 2020), identified three 5 dpf clusters [cluster-217 (c217), c55, c121] that specifically expressed *agt* (Fig. 1B,C, see Fig. S10 for expression of *agt* and other RAAS genes in the context of the entire Atlas). Cluster cell types were identified by the organs and cell types that express the cluster's differentially expressed genes as assayed by *in situ* hybridization experiments accessed mainly at ZFIN, as previously described (Farnsworth et al., 2020); these three clusters specifically expressed hepatocyte genes such as *serpina7*, *apobb.2*, and *hp* (Cheng et al., 2006; Tingaud-Sequeira et al., 2012).

Cortisol and inflammation both control *agt* expression via the glucocorticoid receptor Nr3c1 and by Cebpb, Cebpd, and other enhancer-binding factors (Brasier and Li, 1996; Demura et al., 2015). Most *agt-*expressing liver cells in the Atlas also expressed *nr3c1* at low levels (Figs S2B,C and S10E), but other cell types, including periderm, basal skin cells, ionocytes, fast skeletal muscle, photoreceptors, and vascular endothelium, expressed *nr3c1* at higher levels. Nearly all *agt*-expressing cells in 5 dpf zebrafish livers expressed the *agt*-regulator *cebpb* (Fig. 1D) and *cebpd* (Fig. S2D), although other cell types in the Atlas also expressed these genes (Fig. S10D,F). This result shows that genes expected to initiate the RAAS are already active in hepatocyte cell types in 5 dpf zebrafish.

### Renin

Renin cleaves Ang I from Angiotensinogen (Fig. S1.1). Conserved syntenies and phylogenetics (Fig. S3A,D) support orthology of human *REN* to zebrafish *ren* (ENSDARG00000041858) (Liang et al., 2004). *Renin* is expressed by kidney juxtaglomerular cells in adult mammals and adult zebrafish (Gomez et al., 1988; Hoshijima and Hirose, 2007; Jones et al., 1990; Liang et al., 2004). In mammalian embryos and fetuses, however, *Ren*-expressing cells arise predominantly from the adrenal but also from other primordia, including skin, nervous system, spleen, testis, eyes, and others (Gomez et al., 1988; Jones et al., 2000; Sequeira Lopez et al., 2004). Only three cells in the Atlas expressed detectable levels of *renin* transcript and they were in multiple cell types as in mammalian fetuses, one of which appeared to be an interrenal (adrenal homolog) precursor (c89) (Yan et al., 2020b) as determined by co-expression of steroidogenic genes including *star* and *hsd3b1* (Fig. S3B,C; Fig. S10G). The hypothesis that *Renin*, *Ctsd*, and *Napsa* derived by duplication in stem bony vertebrates (Liang et al., 2004) gains support from our genomic analyses showing that the portions of Hsa1, Hsa11, and Hsa19 that contain these three genes, along with a part of Hsa12, make up four ohnologous regions (Fig. S3E) predicted by two rounds of Vertebrate Genome Duplication (VGD1 and VGD2; Dehal and Boore, 2005; Simakov et al., 2020); ohnologs are paralogs arising in a genome duplication (Wolfe, 2000).

### Angiotensin I

**Fig. 2.**
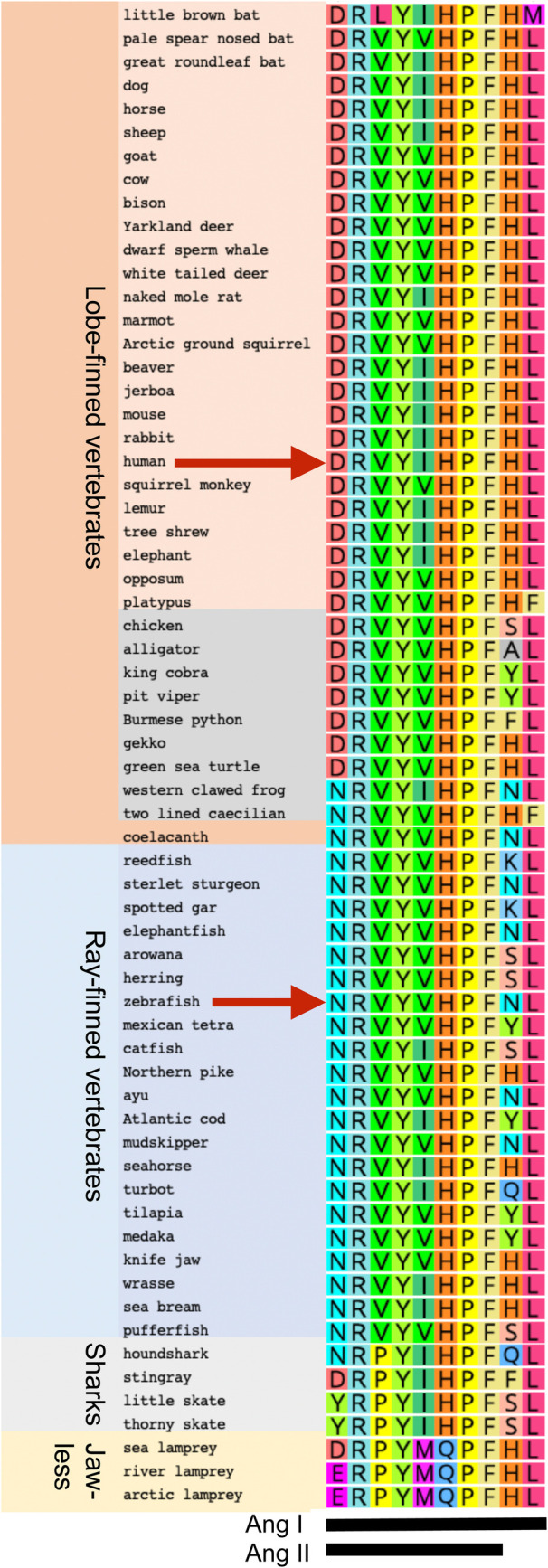
**Evolution of Angiotensin sequences.** Arrows indicate human and zebrafish sequences. Species organized according to published phylogenies (Hughes et al., 2018; Upham et al., 2019). Ang I and Ang II sequences indicated at bottom. Table S1 contains Latin names and accession numbers.

Angiotensin I (Ang I; Fig. S1.1) varies in sequence among vertebrates (Takei et al., 1993). We downloaded Angiotensinogen sequences across vertebrate phylogeny, extracted their Ang I sequence, and found that most placental mammals have the same Ang I sequence as humans, likely the ancestral eutherian state (DRVYIHPFHL, Fig. 2). Some Artiodactyls, like cows and whales, however, have a valine (V) at position 5, as do squirrel-related mammals and some bats. These replacements are lineage-specific because each is embedded in a clade with isoleucine at position 5. The little brown bat *Myotis lucifugus* (Vespertilioniodea) has the most variant Ang I among sampled therians, with leucine rather than the otherwise invariant valine at position 3 and methionine rather than the otherwise invariant leucine at position 10. Vespertilioniodid bats harbor coronaviruses most closely related to SARS-CoV-2 and are the presumed wild source of this zoonotic virus (Anthony et al., 2017; Cotten et al., 2013; Lau et al., 2020; Toosy and O'sullivan, 2019). Monotremes and marsupials have valine rather than isoleucine at position 5, which is the ancestral mammalian state (DRVYVHPFHL) because most non-mammalian tetrapods also have valine at this position. Most non-mammalian sarcopterygians (lobe-finned vertebrates) have, like mammals, aspartic acid at position 1, although basally diverging lobe-finned vertebrates, including amphibia and coelacanth, have asparagine at position 1. Because actinopterygians (ray-finned vertebrates) also have asparagine at position 1, this is likely the ancestral state for all Osteichthyes (bony vertebrates). Although mammals have histidine at position 9, non-mammalian lobe-finned vertebrates have a variety of amino acid residues at this position. Because the most basally diverging lobe-finned vertebrates (clawed frog *Xenopus tropicalis* and coelacanth) both have asparagine at position 9, and because basally diverging ray-finned vertebrates also have asparagine at position 9, the sequence NRVYVHPFNL is likely the ancestral state for lobe-finned vertebrates.

Ang I sequences vary more among ray-finned than lobe-finned vertebrates (Fig. 2). Because coelacanth and several basally diverging ray-finned vertebrates (sturgeon, elephant fish) have the sequence NRVYVHPFNL, this is likely the ancestral Ang I sequence for all bony vertebrates. Several ray-finned vertebrates have isoleucine at position 5, representing independent mutations that happen to match placental mammals. Position 9 is highly variable in ray fins as in lobe fins. Zebrafish Ang I is NRVYVHPFNL, differing from the human form at positions 1, 5, and 9.

The Angiotensin system was likely already active in stem vertebrates because Chondrichthyes (cartilaginous fish) and even agnathans (jawless vertebrates) possess Angiotensinogen genes (Takei et al., 1993). At position 1, some cartilaginous fish have arginine like most ray-finned vertebrates, but others have asparagine or tryptophan, and position 9 is variable. The ancestral Ang I sequence in jawed vertebrates was likely the same as in ancestral bony fishes (NRVYVHPFNL).

Angiotensinogen genes in jawless vertebrates appear to encode an Angiotensin that shares the amino-terminal four residues with mammals but varies in the carboxy-terminal six residues (Wong and Takei, 2011). At position 1, lampreys have either aspartic acid or glutamic acid, but the conserved isoleucine or leucine at position 5 is replaced with methionine followed by glutamine replacing the otherwise invariant histidine. Lamprey Ang II alters cardiovascular dynamics in live lampreys but teleost Ang II (NRVYVHPF) does not (Wong and Takei, 2011), showing that stem jawless vertebrates already had key components of the RAAS but that ligands and receptors evolved differences. Searching for Atg sequences in the non-vertebrate chordate amphioxus returned serine protease inhibitors (SERPINs), but not Angiotensinogen, suggesting that Ang I signaling originated in vertebrates after divergence from non-vertebrate chordate ancestors.

### Angiotensin converting enzyme

**Fig. 3.**
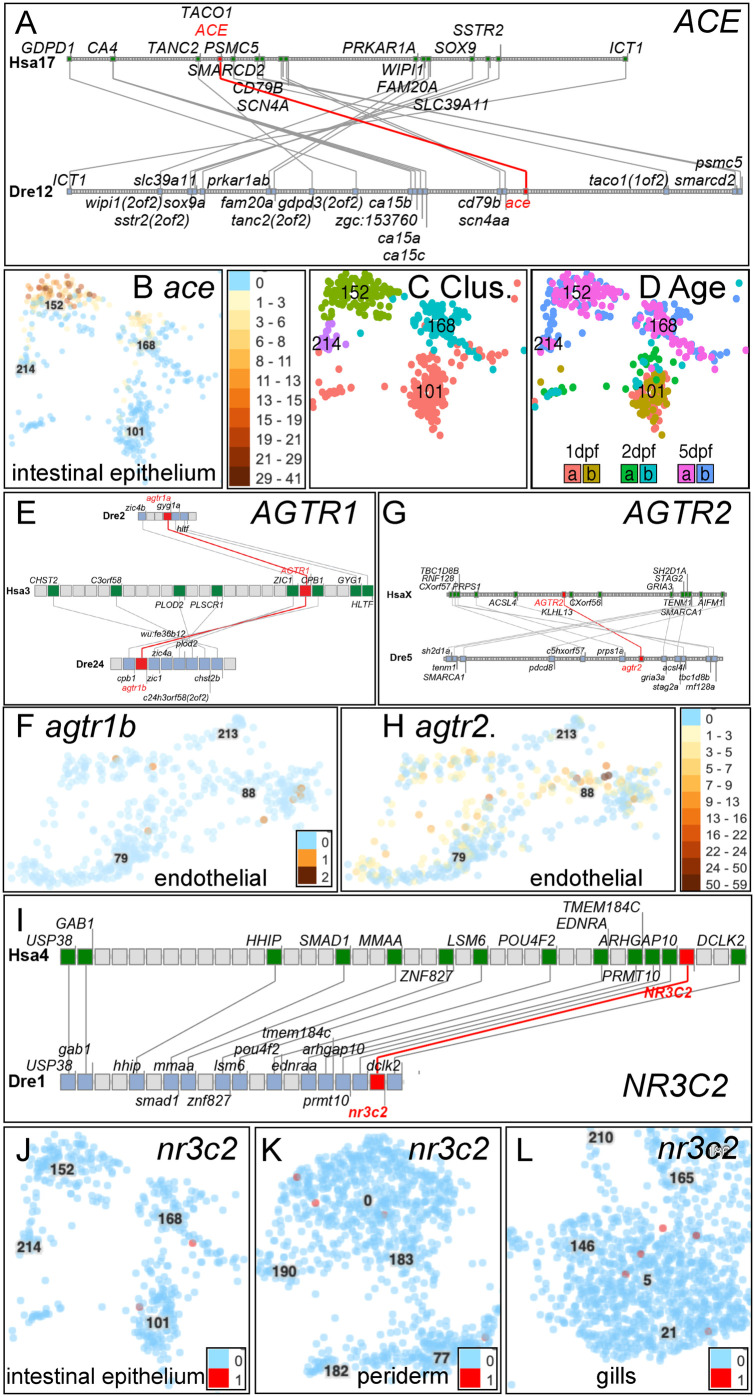
**Genomics and expression of *ace*, *agtr*, and *nr3c2*. (**A) Conserved syntenies verify orthology of zebrafish *ace* to human *ACE*. (B) Expression of *ace* in a specific intestinal epithelial cell type in c152. (C) Clusters c101, c152, c168, and c214 are intestinal epithelial cell types (Farnsworth et al., 2020). (D) The age of each cell, as indicated in the insert (two replicates at each of three developmental stages), showed that *ace* is expressed in a 5 dpf intestinal cell type c152 that does not exist at 1dpf or 2dpf. (E) Double conserved synteny of zebrafish *agtr1a* and *agtr1b* to human *AGTR1*. (F) *agtr1b* was expressed only in endothelial cells (c88). Expression of *agtr1a* was not detected. (G) Conserved synteny of *agtr2* and *AGTR2* confirms orthology. (H) Expression of *agtr2* was detected in c88 and c79 endothelial cells, including some in which *agtr1b* was detected. (I) Conserved syntenies for the aldosterone receptor gene *nr3c2*. (J) Expression detected for *nr3c2* in intestinal epithelial clusters c168 and c101. (K-L) Expression of *nr3c2* in the embryonic periderm (c0, c77) and in larval gills (c5).

Angiotensin converting enzyme (Ace) transforms Ang I to Ang II (Fig. S1.2). Syntenies are conserved between *ace* (ENSDARG00000079166) in zebrafish and *ACE* (ENSG00000159640) in human supporting orthology (Fig. 3A). In humans, *ACE* is expressed four times stronger in the small intestine than in lungs (Fagerberg et al., 2014) and in 5 dpf zebrafish, *ace* is also expressed in the gut (Rauch et al., 2003). In the Atlas, *ace* was expressed almost exclusively in 5 dpf intestinal epithelium cluster c152 (Fig. 3B­-D, Fig. S10H). The most statistically differentially expressed genes in c152 encode fatty acid binding proteins and apolipoproteins (Farnsworth et al., 2020), suggesting that these cells function in lipid biology.

### Angiotensin II

Angiotensin II (Ang II) forms when Ace cleaves two C-terminal amino acid residues from Ang I (Fig. S1.3). Ang II contributes to hypertension, an important COVID-19 comorbidity, and promotes inflammation, which leads to poor COVID-19 outcomes. Human and zebrafish Ang II differ only at the first and fifth residues (DRVTIHPF versus NRVTVHPF, Fig. 2). Importantly, the fish and human peptides act about equally to stimulate heterologous systems (Russell et al., 2001; Vilella et al., 1996). Zebrafish treated with Ang II increase sodium uptake as expected if Ang II function is conserved between human and zebrafish (Kumai et al., 2014).

### Angiotensin II receptor type 1 and type 2

Angiotensin II receptor type 1 and type 2 (Agtr1 and Agtr2, Fig. S1.4) transduce Ang II signals on vascular and other cells. In mammals, Agtr1 mediates inflammation and the production of aldosterone in the adrenal cortex, thereby increasing the kidney's reabsorption of sodium (Higuchi et al., 2007). Agtr2 broadly opposes Agtr1 by decreasing cell proliferation and vasoconstriction (Wang et al., 1998b). Zebrafish has two co-orthologs of *AGTR1* (*agtr1a* (ENSDARG00000018616) and *agtr1b* (ENSDARG00000045443) that reside in double conserved synteny with human *AGTR1*, verifying orthology (Fig. 3E). Human *AGTR1* and *AGTR2* are expressed in vascular endothelial cells; likewise, the Atlas showed *agtr1b* expression strongest in endothelial cells (c88, Fig. 3F, Fig. S10), and in an unidentified type of mesenchyme (c135), likely vascular precursors. In mammals, the kidney controls ion and water balance and expresses *Agtr1* (Miyata et al., 1999), but in fish, ionocytes in the skin control ion and water balance (Dymowska et al., 2012; Guh et al., 2015; Inokuchi et al., 2017; Kwong et al., 2016) although they did not have detectable *agtr1a* or *agtr1b* expression in the Atlas despite the fact that eel ionocytes express a protein that binds a mouse antibody to Agtr1 (Marsigliante et al., 1997). Expression of *agtr1a* was not detected in the Atlas.

Zebrafish has a single ortholog of human *AGTR2* (Wong and Takei, 2013) with conserved syntenies (Fig. 3G). *In situ* hybridization identified *agtr2* (ENSDARG00000035552) expression in zebrafish endothelia (Wong et al., 2009), which the Atlas confirmed (Fig. 3H). Expression of *agtr2* was also detected in 5 dpf mesenchyme (c135) and in 5 dpf enteric smooth muscle cells (c197) (Fig. S10J). Agtr antagonists that patients take to control hypertension also ameliorate zebrafish models of heart failure (Quan et al., 2020), showing conserved structure and function of angiotensin receptors.

In mammals, Agtr1 mediates adrenal secretion of aldosterone (Rainey et al., 2004). Teleosts have no aldosterone, lacking an ortholog of the aldosterone-synthesizing enzyme *Cyp11b2*, but nevertheless have an ortholog of *Nr3c2*, which encodes the aldosterone (mineralocorticoid) receptor (Bridgham et al., 2006). In fish, Nr3c2 is stimulated either by the aldosterone precursor 11-deoxycorticosterone or by cortisol (Kumai et al., 2014; Sturm et al., 2005). Human and zebrafish share strong conserved syntenies around *NR3C2* and *nr3c2* (ENSDARG00000102082, Fig. 3I). In the Atlas, *nr3c2* was expressed in a few cells of 5 dpf intestinal epithelium (c101, c168), in periderm in 1 dpf and 2 dpf embryos (c0), in gills of 5 dpf larvae (c5) (Fig. 3J-L), and in one NaK ionocyte cell (c128); these cell types contribute to water and salt balance at these stages (Dymowska et al., 2012; Fu et al., 2010; Hoffmann et al., 2018; Rombough, 2007). Zebrafish embryos also expressed *nr3c2* in some retinal progenitors (Farnsworth et al., 2020) and a few other scattered cells in the Atlas (Fig. S10).

### Angiotensin I converting enzyme-2

**Fig. 4.**
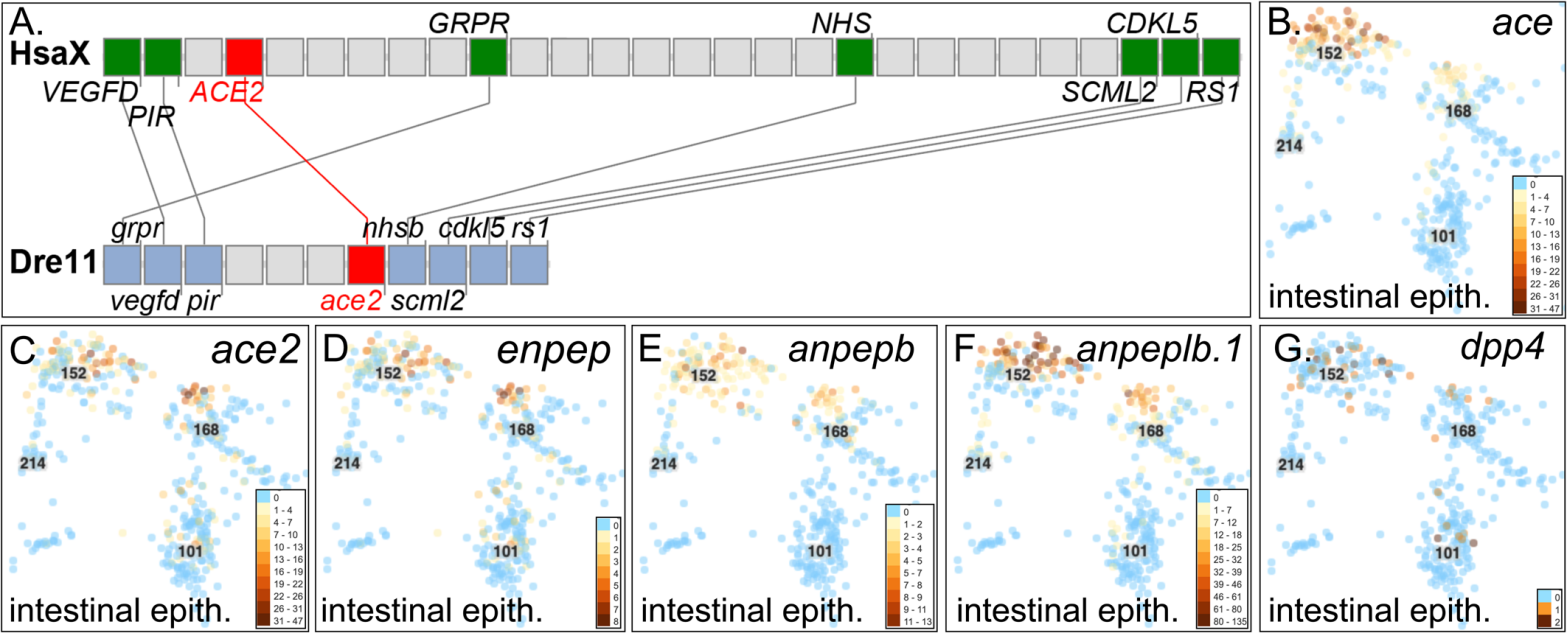
**Genomics and expression of *ace2, enpep, anpep* and *dpp4*.** (A) Conserved synteny supports orthology of human *ACE2* to zebrafish *ace2.* Expression of *ace* (B), *ace2* (C), *enpep* (D), *anpepb* (ENSDARG00000041083) (E), *anpeplb.1* (ENSDARG00000103878) (F), and *dpp4* (G). All were expressed mainly in the intestinal epithelial cell type c152.

Angiotensin I converting enzyme-2 (Ace2, Fig. S1.7) normally functions to remove an amino acid from Ang II to form Ang 1-7 (the seven amino terminal residues in Fig. 2). Phylogenetics (ENSGT00940000158077, Fig. S4A) and conserved syntenies (Fig. 4A) support orthology of human *ACE2* (ENSG00000130234) to zebrafish *ace2* (ENSDARG00000016918) (Chou et al., 2006). Human *ACE2* is expressed three to four times stronger in the intestine than in gall bladder, kidney, and testis, sixfold stronger than in heart, and 190-fold stronger than in lungs (Fagerberg et al., 2014; Hamming et al., 2004; Xu et al., 2020), perhaps providing a route of infection. In the Atlas, *ace2* was expressed exclusively in c152, the cluster that expressed *ace* (Fig. 4B, C; Fig. S10L). Co-expression of *ace* and *ace2* identifies this digestive tract cell type as a likely mediator of RAAS activity. SARS-CoV-2-mediated down-regulation of its receptor ACE2 in gut cells may help explain gastrointestinal pathologies of COVID-19 (Guan et al., 2020; New York Department of Health, 2020).

### Mas1

Mas1 is a receptor for Ang 1-7 (Fig. S1.8,S9) (Santos et al., 2003). *MAS1* is present in mammals, Sauropsids, and amphibians, and a related gene (ENSLACG00000015240) is found in coelacanth, a basally diverging lobe-finned vertebrate, but it is missing from sequenced genomes of ray-finned vertebrates (Fournier et al., 2012). Conserved syntenies suggest a mechanism for this situation. In human, *MAS1* lies in the gene sequence: *TCP- MRPL18-MAS1-IGF2R-SLC22A1-SLC22A2.* Zebrafish orthologs of genes to the right of *MAS1* are adjacent as in human, but zebrafish orthologs of genes to the left of *MAS1* are on different chromosomes in both zebrafish and spotted gar, a basally diverging ray-finned vertebrate (Braasch et al., 2016). Thus, the genomic neighborhood expected to contain the ray-fin ortholog of *MAS1* is rearranged with respect to the human genome, consistent either with gene loss due to a chromosome rearrangement breakpoint in stem ray-fin vertebrates, or to the origin of *MAS1* in stem lobe-fin vertebrates. A different G-protein coupled receptor (GPCR) in this large protein family (Rinne et al., 2019) may act as a zebrafish Ang 1-7 receptor.

### Enpep

Glutamyl aminopeptidase (Enpep) converts Ang II to Ang III (Fig. S1.10), which stimulates systemic blood pressure similar to Ang II (Mizutani et al., 2008; Yugandhar and Clark, 2013). Zebrafish has a single *enpep* (ENSDARG00000057064) gene on Dre13; phylogenetic analysis (ENSGT00940000156946) and conserved syntenies with human *ENPEP* on Hsa4 (Fig. S4B,C and S10 M) verify orthology. Cells in the Atlas that expressed *enpep* also expressed *ace* and *ace2* in c152 and c168 (Fig. 4B-D), increasing the relevance of this cell type to COVID-19.

### Anpep

Alanyl aminopeptidase (Anpep) metabolizes Ang III to Ang IV (Fig. S1.11) and is a receptor for the the common cold coronavirus HCoV-229E (Fehr and Perlman, 2015). Humans have a single *ANPEP* gene, but zebrafish has five *ANPEP*-related genes distributed on three chromosomes. Analysis of gene trees and conserved syntenies supports the model that before the divergence of lobe-finned and ray-finned vertebrates, tandem duplication produced *anpep* and *anpepl* (Figs S5, S6). The *anpepl* gene was lost in stem lobe-finned vertebrates. In ray-finned vertebrates, the TGD produced ‘*a*’ copies of both *anpep* and *anpepl* on one chromosome and ‘*b*’ copies of both genes on the duplicate chromosome. One chromosome became Dre7 containing both *anpepa* (ENSDARG00000036809, ZFIN *si:ch211-106j24.1*) and *anpepla* (ENSDARG00000089706, ZFIN *si:ch211-276a23.5*). The ‘b’ copy fates were more complex: first, a previously identified chromosome fission event (Nakatani and McLysaght, 2017) separated *anpepb* (ENSDARG00000041083, ZFIN: *anpepa*) on Dre18 from the temporary *anpeplb* gene on Dre25, which then duplicated tandemly to form *anpeplb.1* (ENSDARG00000103878, ZFIN: *anpepb*) and *anpeplb.2* (ENSDARG00000097285, ZFIN: *si:ch211-147g22.5*). This genomic analysis clarifies relationships of zebrafish *ANPEP* paralogs necessary to connect human and zebrafish biology.

In human, ANPEP is expressed about six times stronger in the duodenum and small intestine than in the third highest organ, the kidney (Fagerberg et al., 2014). In zebrafish, *anpepb* and *anpeplb.1* were expressed in the same intestinal cell type as *ace*, *ace2*, and *enpep* (c152) (Fig. 4E,F). In contrast, *anpepa* was not expressed in the intestine, but was expressed in a 5 dpf blood vessel cell type (c201) different from the cell types expressing Angiotensin receptors *agtr1b* and *agtr2*, as well as in a few cranial neural crest cells that had developed by 5dpf (Fig. S10N,O). The other two *ANPEP*-related zebrafish genes (*anpepla*, *anpeplb.2*) were not expressed in the Atlas. These five zebrafish *ANPEP-*related genes may share among them the original functions of the ancestral *anpep* gene, some of which might have been retained by human *ANPEP*.

### DPP4

Dipeptidyl peptidase-4 (DPP4) is the receptor for the Middle East Respiratory Syndrome coronavirus MERS-CoV (Raj et al., 2013). DPP4 inhibitors also inhibit Ace and are anti-diabetic (Abouelkheir and El-Metwally, 2019). Ang II stimulates DPP4 activity in the mammalian kidney (Aroor et al., 2016). Zebrafish *dpp4* (ENSDARG00000079420) shares syntenies with human *DPP4*, verifying orthology (Fig. S7). In the zebrafish Atlas, *dpp4* was expressed exclusively in cells that express *ace*, *ace2, enpep, anpepa*, and *anpepb* (Fig. 4G; Fig. S10P), improving resolution of *in situ* hybridizations (Thisse and Thisse, 2004).

### Slc6a19

**Fig. 5.**
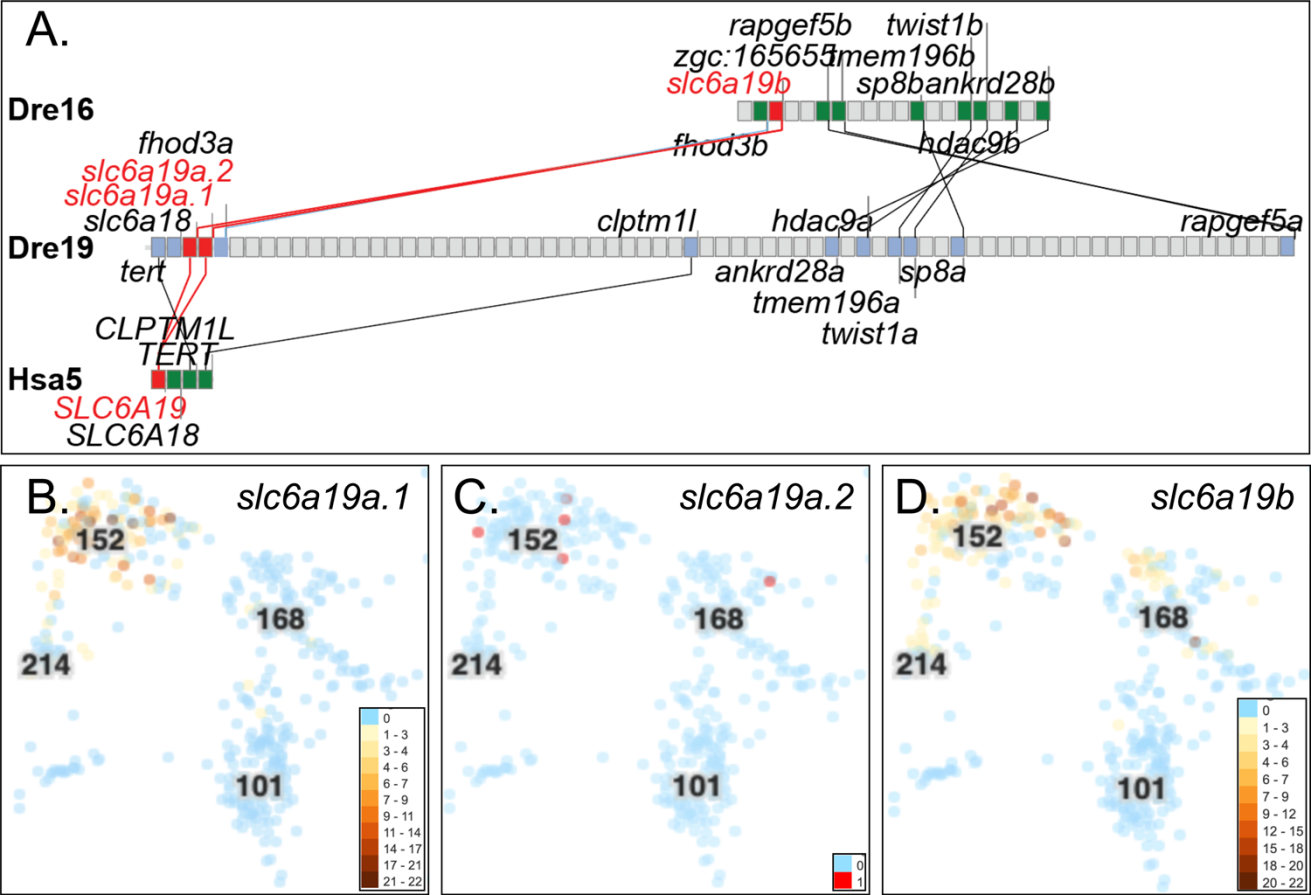
**Conserved syntenies and expression of *SLC6A19*-related genes*.*** (A) Conserved syntenies show double conserved synteny of a part of Dre16 containing *slc6a19b* and a portion of Dre19 containing *slc6a19a.1*, and *slc6a19.2* and their relationship to human chromosome Hsa5 around *SLC6A19*. (B-D) Expression of *slc6a19a.1*,* slc6a19a.2*, and *slc6a19b*, respectively, in the zebrafish Atlas in c152 and c168, which represent the same larval intestinal epithelial cells that expressed *ace* and *ace2*.

Slc6a19 (Fig. S1.12) is a neutral amino acid transporter and forms a heterodimer with ACE2 (Yan et al., 2020a). Zebrafish has three *SLC6A19* co-orthologs: *slc6a19a.1* (ENSDARG00000018621) and *slc6a19.2* (ENSDARG00000091560) are tandem duplicates on chromosome Dre19 and *slc6a19b* (ENSDARG00000056719) on Dre16 represent TGD duplicate loci according to conserved syntenies and phylogenetics (Fig. 5A; Fig. S8). *SLC6A19* is expressed about equally in small intestine, duodenum, and kidney, about 22-fold stronger than the next organ, the colon (Fagerberg et al., 2014). In the Atlas, all three *slc6a19* paralogs were expressed exclusively in the same intestinal cell type as *ace2* (c152, Fig. 5B-D; Fig. S10Q-S), as expected if Slc6a19 proteins interact in a similar way in zebrafish and human.

Hints for the role of Slc6a19 in COVID-19 come from population genetic studies. *SLC6A19* and *SLC6A18* paralogs lie adjacent in human and zebrafish genomes and, along with *SLC6a20* on Hsa3, represent a closely related gene clade (Kristensen et al., 2011). In birds, *Slc6a19* (ENSJHYG00000013510) and *Slc6a18* (ENSJHYG00000013530) are adjacent and syntenic with *Slc6a20* (ENSJHYG00000002729). These data imply origin by tandem duplications followed by translocation in mammals after divergence from birds. Like *Slc6A19, Slc6A20* is expressed in intestinal enterocytes and kidney cells and interacts functionally with ACE2 (Romeo et al., 2006; Vuille-dit-Bille et al., 2015). Importantly, *SLC6A20* is at the peak of a genome-wide association study for poor outcomes from COVID-19 (Ellinghaus et al., 2020). These findings raise the novel hypothesis that SLC6A20 variants contribute to variation in COVID-19 outcomes due to differences in expression or protein function related to interactions with ACE2, the SARS-CoV-2 receptor.

### Adam17

**Fig. 6.**
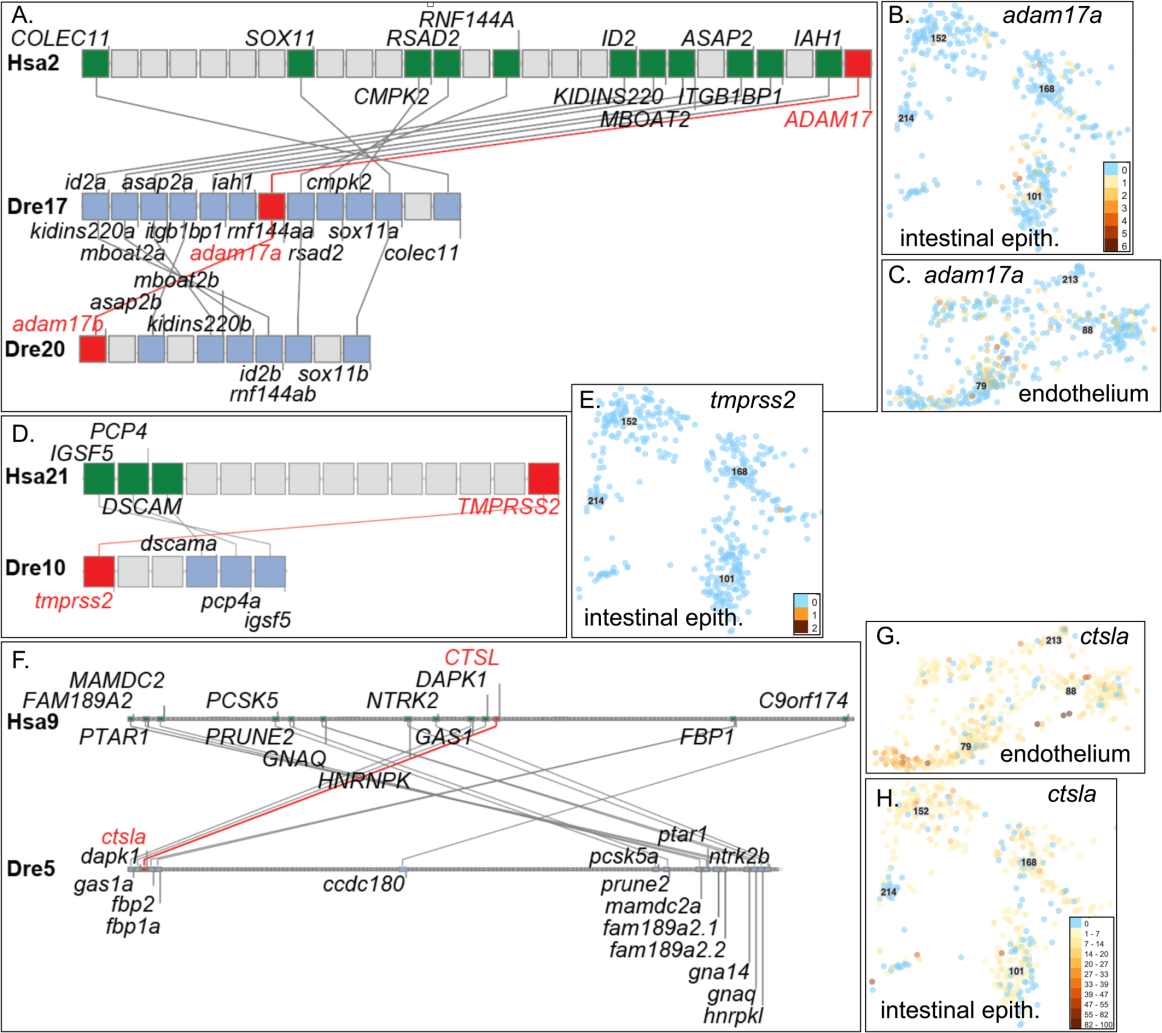
**Conserved syntenies and expression of a*dam17*, *tmprss2* and c*tsl*.** (A) Double conserved synteny between Dre17 and Dre20 confirming co-orthology of *adam17a* and *adam17b* to *ADAM17* and their origin in the TGD*.* (B) Expression of *adam17a* in intestinal epithelium and (C) vascular endothelium. (D) Conserved syntenies verify orthology of *tmprss2* to *TMPRSS2*. (E) Expression of *tmprss2* was detected in one cell in intestinal epithelium c168 and in only 12 other cells broadly dispersed in the atlas. (F) Human *CTSL* shares conserved syntenies with zebrafish *ctsla.* (G) Expression of *ctsla* in the endothelium and (H) intestinal epithelium.

Adam17 (Fig. S1.13) is a metalloendopeptidase widely expressed in human cells (Fagerberg et al., 2014) that cleaves the membrane isoform of Ace2 and makes the soluble protein sAce2 (Lambert et al., 2005). Zebrafish has two copies of *adam17* in double conserved synteny with human *ADAM17* (Fig. 6A) showing they are TGD co-orthologs of *ADAM17*. The Atlas showed *adam17a* (ENSDARG00000043213) expression stronger in embryonic intestinal epithelium than in larval *ace2*-expressing c152 cells (Fig. 6B), and in several vascular endothelial cells (Fig. 6C; Fig. S10T). The duplicate (*adam17b*, ENSDARG00000093093) was expressed in just a few widely dispersed individual cells (Fig. S10U). It is unknown whether zebrafish has soluble Ace2.

### Tmprss2

Tmprss2 (Fig. S1.16) is a transmembrane serine protease that activates the coronavirus spike protein, stimulating virus entry into cells (Hoffmann et al., 2020; Millet and Whittaker, 2015; Walls et al., 2020). Human *TMPRSS2* expression is highest in prostate, followed by the digestive system, and below that, the lung (Fagerberg et al., 2014). *TMPRSS2* in human and *tmprss2* (ENSDARG00000098686) in zebrafish occupy regions of conserved synteny, confirming orthology (Fig. 6D). Only one intestinal epithelial cell expressed *tmprss2* in the Atlas (c168, Fig. 6E) as well as three kidney cells (Fig. S10), so its relevance to possible spike protein activation is unknown.

### Cathepsin L

Cathepsin L (Ctsl) can cleave the spike protein of SARS-CoV (Simmons et al., 2005). *CTSL* on Hsa9 shows conserved synteny with zebrafish *ctsla* (ENSDARG00000007836) on Dre5 (Fig. 6F). Zebrafish *ctslb* (ENSDARG00000074306) on Dre12 is in a cluster of 12 tandemly duplicated paralogs that do not show conserved synteny with human *CTSL*, thus not supporting that it is a TGD ohnolog of *ctsla*. In the Atlas, *ctslb* was expressed exclusively in c207 (Fig. S10X), the hatching gland (Rauch et al., 2003; Thisse and Thisse, 2004), which digests the egg shell. In contrast, *ctsla* is expressed in the endothelium (Fig. 6G), along with *agtr1b* and *agtr2* (Fig. 3F,H), in the intestine with *ace* and *ace2*, and in liver, kidney, spleen, and gill, and weaker broadly in the nervous system. Because Ctsl cleaves the spike protein of SARS-CoV (Simmons et al., 2005), it may also activate the spike protein of SARS-CoV-2; expression of *ctsla* in zebrafish *ace2-*expressing cells makes it a likely candidate to model SARS-CoV-2 function in COVID-19.

### Apelin

**Fig. 7.**
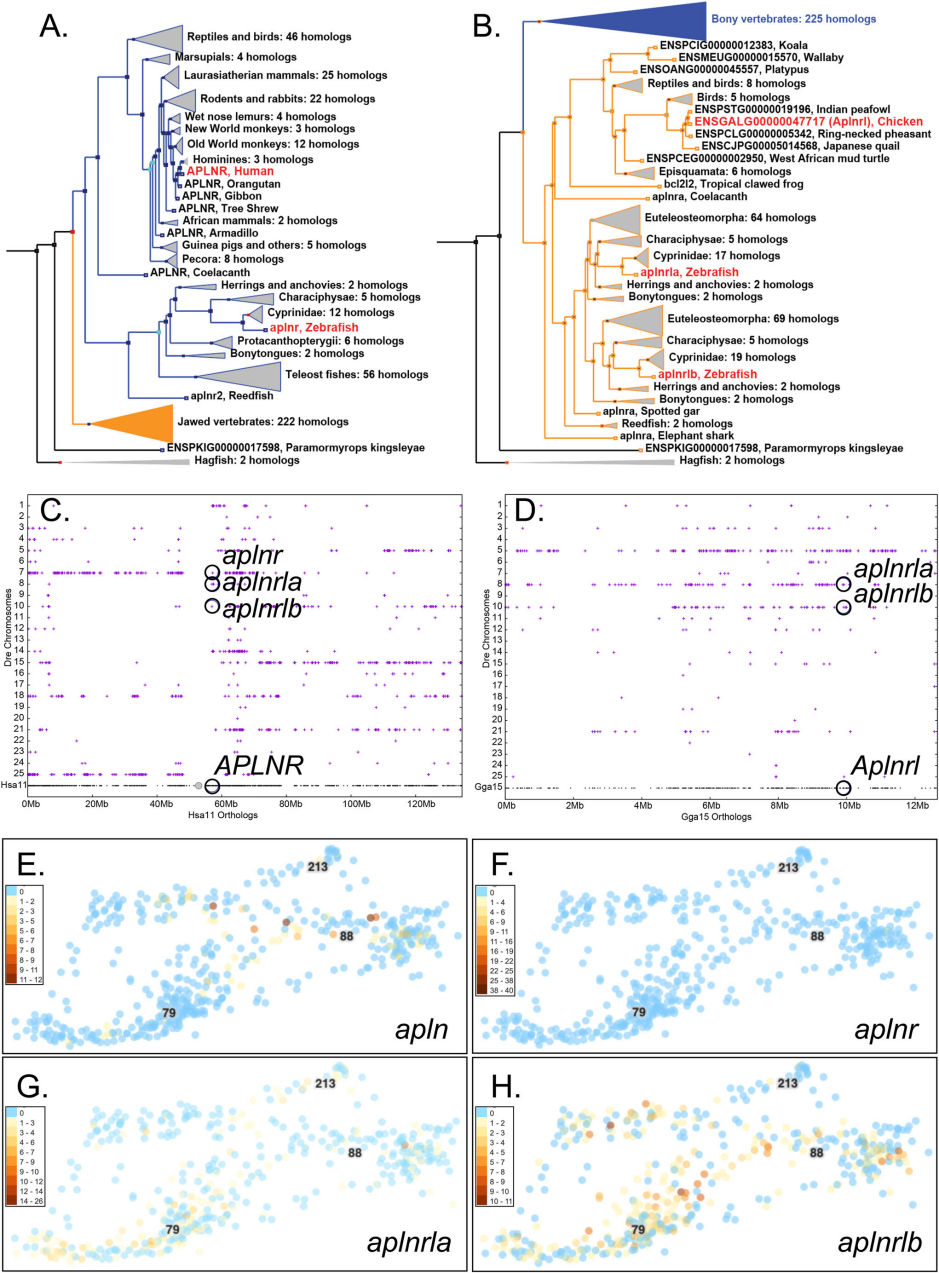
**Genomics and expression of Apelin and its receptors.** (A) Phylogenetic tree (ENSGT01000000214406) with the *APLNR/’aplnr2’* (suggested new name *aplnr*, ENSDARG00000004447) subtree (blue in panel B) expanded. (B) The same tree with the ‘*aplnra’/’aplnrb’* (suggested new name *aplnrla*, ENSDARG00000002172)/*aplnrlb* (ENSDARG00000036670) subtree (orange in panel A) expanded. (C) A dot plot representing orthologs and paralogs of Hsa11 genes versus zebrafish (Dre) chromosomes plotted directly above the location of each Hsa11 gene showing extensive conservation with Dre7, the site of *aplnr*, but not Dre8 or Dre10, the locations of *aplnrla* and *aplnrlb*, respectively. (D) A dot plot of chicken chromosome Gga15 versus zebrafish chromosomes showing the sites of *aplnrla* and *aplnrlb* and their ortholog in chicken (*Aplnrl*, ENSGALG00000047717) identified in the tree in B. (E-H) Expression of *apln*, *aplnr*, *aplnrla*, and *aplnrlb* in endothelial cells.

Apelin (Apln, Fig. S1.17-18) peptides oppose Ang II effects (De Mota et al., 2004; Dray et al., 2008; Kasai et al., 2004; Szokodi et al., 2002). Zebrafish has a single copy of *apln* (ENSDARG00000053279, see ENSGT00390000014020) (Quertermous, 2007; Scott et al., 2007; Zeng et al., 2007) that has strong conserved synteny with human *APLN* (Fig. S9). In zebrafish, *apln* is expressed in axial chordamesoderm, notochord, heart primordium, and vasculature (Helker et al., 2015; Kwon et al., 2016; Scott et al., 2007; Zeng et al., 2007), which the Atlas confirms, in endothelial cell type c88 at 1, 2 and 5 dpf but not in the other two endothelial cell types (Fig. 7E). The co-expression of venous markers (*efnb2a*, *hey2*, and *tbx20*) and arterial markers (*flt4*, *ephb4*) (Fischer et al., 2004; Lawson et al., 2001; Saint-Geniez et al., 2003; Szeto et al., 2002; Thompson et al., 1998; Wang et al., 1998a; Zhou et al., 2015) show that *apln* was expressed in venous endothelium (Fig. S10Ya-d). In addition, *apln* was expressed in cardiac muscle (c205) at 1 and 2 dpf, cardiac neural crest (c69) at 1 dpf, vessel precursors (c117) at 1 dpf, NCC type ionocytes at 1 and 2 dpf, photoreceptors (c115) at 5 dpf, and notochord (c158, c149) (Fig. S10Y), extending prior studies (Helker et al., 2015; Kwon et al., 2016; Scott et al., 2007; Zeng et al., 2007). A zebrafish *apln*-knockout mutant allele had normal vasculogenesis (Helker et al., 2015), suggesting that Apln is not required in vessels that secrete it, but that it regulates the appropriate migration of myocardial progenitors (Helker et al., 2015; Scott et al., 2007; Zeng et al., 2007).

### Apln receptor

Apln receptor (Aplnr, alias Agtr1l1 or Apj) has a single copy gene in mammals but three in zebrafish called ‘*aplnra’* (ENSDARG00000002172), ‘*aplnrb’* (ENSDARG00000036670), and ‘*aplnr2’* (ENSDARG00000004447, also called *aplnr3a*; Zhang et al., 2018). Phylogenetic analysis for *APLNR*-related genes (ENSGT01000000214406) showed that human *ALPNR* (ENSG00000134817 on Hsa11) branched with chicken *Alpnr* (ENSGALG00000004841) and zebrafish ‘*aplnr2’* (ENSDARG00000004447), as expected if these three genes were orthologs and ‘*aplnr2*’ is misnamed (Fig. 7A). Genomic analysis showed that about half of Hsa11, including Hsa11p and the proximal part of Hsa11q that contains *ALPNR*, is orthologous to the portion of Dre7 that contains ‘*aplnr2’*, verifying a nomenclature mismatch (Fig. 7C). The TGD copy of this part of Hsa11 includes parts of Dre25 and Dre14 or Dre21, which lost the TGD co-ortholog of ‘*aplnr2’* (Fig. 7C). Dre8 and Dre10, the locations of ‘*aplnra’* (ENSDARG00000002172), and ‘*aplnrb’* (ENSDARG00000036670), respectively, had substantially fewer conserved syntenies with Hsa11, suggesting that they represent paralogons from earlier genome duplication events in stem vertebrates (VGD1 and VGD2). These phylogenetic and conserved synteny analyses converge to support the orthology of *APLNR* to ‘*alpnr2’* (ENSDARG00000004447) in zebrafish. This conclusion differs from an earlier report (Zhang et al., 2018) that did not consider phylogenetic analysis or analyze conserved synteny for the relevant parts of Hsa11 to Dre7. These considerations suggest that changing the name of zebrafish ‘*alpnr2’* (ENSDARG00000004447) to *alpnr* would conform to nomenclature rules and better connect zebrafish medical models to human biology.

Analysis of zebrafish ‘*aplnra’* and ‘*aplnrb’* in the same tree (ENSGT01000000214406) showed that most teleost clades have orthologs of each gene, that the bony tongues (Osteoglossiformes), which branch deep within teleosts, root each clade; furthermore, spotted gar and reedfish serve as pre-TGD outgroups, as expected from historical species relationships (Fig. 7B). The sister group of the ray-finned ‘*aplnra’+‘alpnrb’* clade is a lobe-finned vertebrate clade rooted on coelacanth and amphibia and includes ‘reptiles’, birds, monotremes and marsupials, but no eutherians (Fig. 7B), which we here call the *Aplnrl* clade. Conserved syntenies showed that chicken (*Gallus gallus*) chromosome Gga15, which contains *Aplnrl* (ENSGALG00000047717), has orthologs on both Dre8 and Dre10, the sites of ‘*aplnra’* (new *aplnrla*) and ‘*aplnrb’* (new *aplnrlb*), respectively, as well as Dre5 (Fig. 7D). These conserved syntenies verify that: 1) *Aplnrl* was present in the last common ancestor of human and zebrafish; 2) *Aplnrl* was lost from eutherians after they diverged from marsupials; and 3) *aplnrla* and *aplnrlb* are TGD paralogs.

These analyses support the hypothesis that the last common ancestor of zebrafish and human had at least two *Aplnr*-related genes; one became *APLNR* in human and *alpnr* (ENSDARG00000004447, old ‘*alpnr2*’) in teleosts and the other was lost in eutherians but retained in other lobe-fin vertebrates and ray-fin vertebrates, and subsequently duplicated in the TGD, becoming *aplnlra* (ENSDARG00000002172, old ‘*aplnra’*) and *aplnrlb* (ENSDARG00000036670, old ‘*aplnrb’*) in zebrafish, thus better connecting zebrafish to human biology.

Atlas cells expressing *aplnr* (ENSDARG00000004447) occupied midline fates, including prominently the floorplate (c176), hypochord (c218), and scleroderm (c33), as well as an embryonic intestinal epithelium cell type (c101) (Fig. S10Z). Expression of *alpnr* was detected in the spleen and heart of zebrafish adults by qPCR (Zhang et al., 2018) and the Atlas confirmed expression in larval spleen cluster c162, weak in three cardiac neural crest cells (c69) at 1 dpf, a single cardiac muscle cell (c205) at 2 dpf, and a single 5 dpf endothelial cell (Fig. 7F). Expression of *aplnrla* was strongest in endothelial cell cluster c79, which also expresses arterial markers *flt4* and *ephb4* (c88, Fig. 7G); thus, venous cells were expressing the ligand gene *apln* (Fig. 7E) and arterial cells were expressing the receptor gene *aplnrla*. In addition, *aplnrla* was expressed in several clusters of basal skin cells (c46, c70, c108, c118, c145), in the pharyngeal endoderm (c45, c179), in the retina (c83) and lens (c106) (Fig. S10AA), confirming and extending previous reports (Helker et al., 2015; Kwon et al., 2016; Zhou et al., 2015). Expression of *aplnrlb* was detected in the Atlas both in venous and arterial cell clusters (c79 and c88, Fig. 7H), as well as in a few slow muscle cells at 1 dpf (c209) and several myoblasts at 2 and 5 dpf (c84, c44) (Fig. S10BB), confirming *in situ* hybridization studies (Tucker et al., 2007). These single-cell gene expression clusters in zebrafish are consistent with Apelin signaling in COVID-19 comorbidities in human because they act in cardiovascular development (Deshwar et al., 2016; Scott et al., 2007; Zeng et al., 2007).

### What is the *ace2-*expressing cell type?

**Fig. 8.**
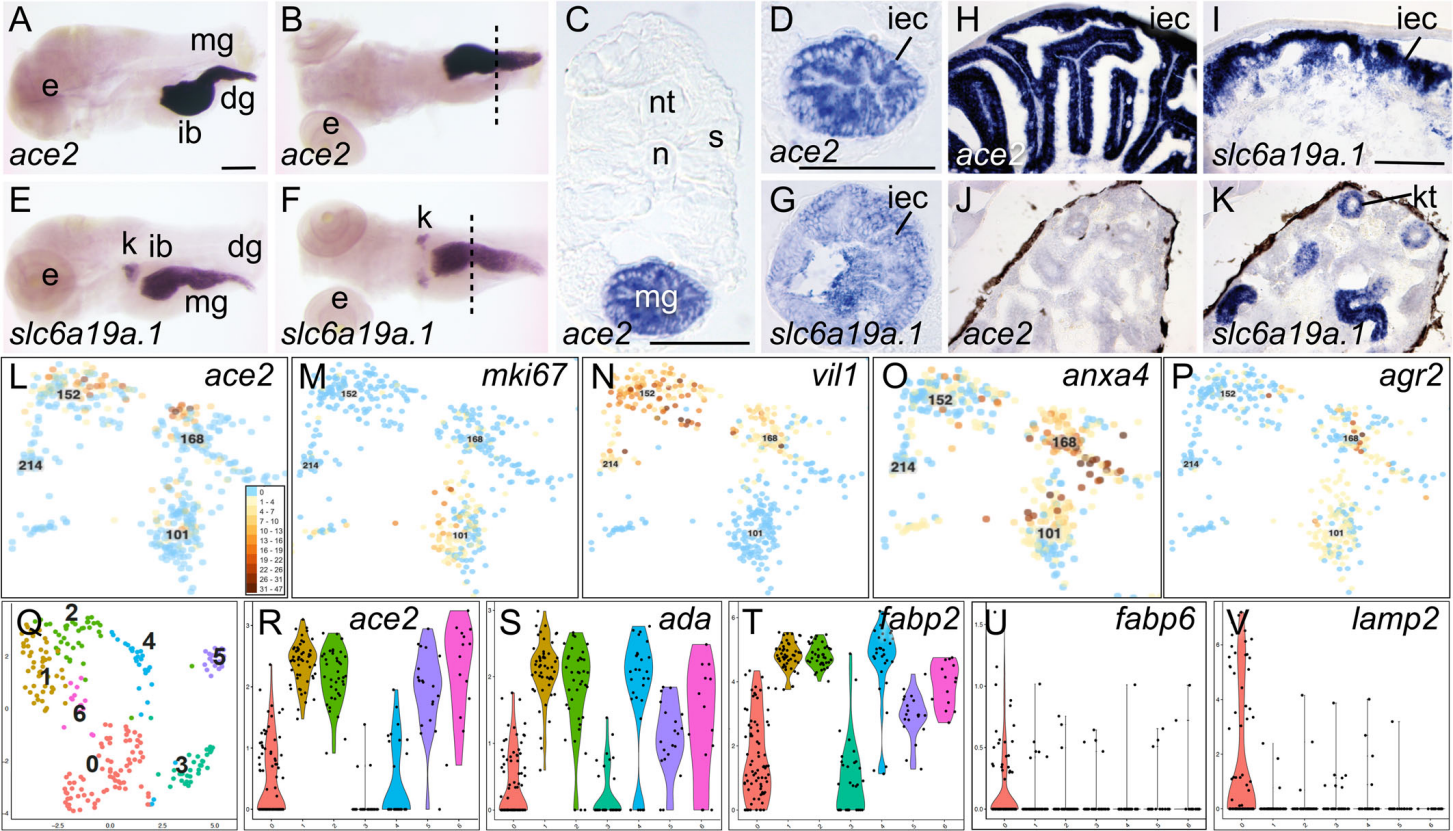
**Identifying the *ace2*-expressing cell type.** (A-K) *In situ* hybridization for *ace2* and *slc6a19a.1.* (A-G) 5 dpf larvae, (A) lateral view and (B) ventral view of *ace2* expression. (C) Cross-section of larva stained for *ace2* expression at level of the dotted line in B. (D) Close-up of the middle gut cross-section showing intestinal epithelial cells expressing *ace2*. (E) Lateral view and (F) ventral view of *slc6a19a.1* expression. (G) Cross-section at level of the dotted line in F. (H-K) Adults. H, *ace2* expression in middle gut, with (J) no expression in kidney. (I,K) *slc6a19a.1* expression in middle gut and kidney, respectively. (L-P) Feature plots of intestinal epithelial cells expressing *ace2* and marker genes for intestinal stem cells (*mki16*), enterocytes (*villin, vil1*), enteroendocrine cells (*anxa4*), and goblet cells (*agr2*). (Q) Umap plot for subclustering of cells in original c152, c168, and c214. (R) Violin plot for *ace2* in the subclusters. (S-V) Violin plots for genes representing regions along the mouse intestine, including (S) duodenum (*ada*); (T) jejunum (*fabp2*); (U) ileum (*fabp6*); and (V) colon (*lamp2*). dg, distal gut; e, eye; ib, intestinal bulb; iec, intestinal epithelial cell; k, kidney; kt, kidney tubule; mg, middle gut; n, notochord; nt, neural tube; s, somite. Scale bar in A for A, B, E, F, 100 μm; in C, 50 μm; in D for D and G, 50 μm; in I for H-K, 100 µm.

To find out, we performed *in situ* hybridizations for *ace2* and *slc6a19* in 5 dpf larvae and adults. Wholemounts showed *ace2* expression exclusively in the digestive tract (Fig. 8A,B). Cross sections verified specific expression in intestinal epithelial cells (Fig. 8C,D). Probe for *slc6a19a.1* detected expression in intestinal epithelial cells and in the developing kidney (Fig. 8E-G), as in mammals (Broer et al., 2011; Kleta et al., 2004). These expression domains persisted in adults (Fig. 8H-K).

To identify *ace2-*expressing cells at single-cell resolution, we explored marker genes mouse and other organisms express in intestinal stem cells (*mki67*, *pcna*, *bmib*, Aghaallaei et al., 2016; Yu et al., 2018) absorptive enterocytes (*alpi*, *vil1*, *slc34a2a*, Makky et al., 2007; Wallace et al., 2005; Yang et al., 2012), peptide hormone-secreting enteroendocrine cells (*gip*, *ccka*, *cckb*, *ghrl*, *gcga*, *gcgb*, *pyyb*, *anxa4*, Crosnier et al., 2005; Sommer and Mostoslavsky, 2014; Zhang et al., 2014), and mucus-secreting goblet cells (*mucin* genes, *agr2*, Komiya et al., 1999; Lai et al., 2016; Treveil et al., 2020). Within the intestine, the zebrafish ortholog of mouse stem cell markers (e.g. *mki67*) were expressed almost exclusively in c101 in 1 dpf and 2 dpf cells, failing to overlap the 5 dpf *ace2*-expressing cells (Fig. 8L,M), and in other rapidly proliferating cells (Fig. S10CC). Enterocyte markers (e.g. *villin*, *alpi.2*, *slc34a2a*) were expressed strongest in c152 but also in some c168 and c214 cells, mimicking *ace2* (Fig. 8L,N, Fig. S10DDa, DDb, DDc). The few cells expressing genes encoding enteroendocrine peptide hormones in c152 were also expressing *ace2*, but more enteroendocrine cells were in c168, as verified by expression of *anxa4*, a marker for enteroendocrine cells (Crosnier et al., 2005; Zhang et al., 2014) (Fig. 8L,O; Fig. S10EE, see also *ccka* in Fig. S10FF). In the intestine, goblet cell markers (e.g. *agr2*) (Komiya et al., 1999; Lai et al., 2016), were expressed mainly in c168, not co-expressed with *ace2* (Fig. 8L,P) [*agr2* was also expressed strongly in taste buds (c196) and otic cells (c93)]; goblet cell mucin genes *muc2.1* and *muc2.2* were not expressed in the Atlas. These results show that zebrafish *ace2-*expressing cells are mostly a type of enterocyte, as in mammals (Hamming et al., 2004), although some may have an enteroendocrine function in 5 dpf larvae.

Regions along the length of the zebrafish digestive tract have gene expression domains corresponding to regions along the mammalian intestine, including duodenum, jejunum, ileum, and colon (Lickwar et al., 2017). To learn which of these regions most strongly expresses *ace2*, we subclustered the three 5 dpf intestinal cell clusters (c152, 168, 214) and identified seven subclusters (Fig. 8Q). Expression of *ace2* was especially strong in subclusters-1, -2, -5, and -6 (Fig. 8R). Genes encoding markers of anterior regions, including *ada* (mouse duodenum, Tang et al., 2014) and *fabp2* (mouse jejunum; Larsson et al., 2012), were expressed mainly in subcluster-1, -2, -4, -5, and -6 (Fig. 8S,T; Fig. S10SHH,II) similar to *ace2*. Zebrafish orthologs of mouse ileum markers, such as *fabp6* (Fig. 8U) and *slc10a2* (Fig. S10JJ,KK) were expressed almost exclusively in subcluster-0, which had a relatively low expression of *ace2*. Genes marking the distal region of the zebrafish digestive tract (mouse colon), like *lamp2* (Fig. 8V), were expressed most strongly in subcluster-0, and stronger in gills and endothelium (Fig. S10LL). These results show that *ace2* is expressed in enterocytes along the length of the zebrafish intestine, with the strongest expression in proximal regions corresponding to the mouse duodenum and jejunum, confirming *in situ* hybridization results (Fig. 8A-K).

## DISCUSSION

These analyses show that: (1) nearly all components of the human Renin-Angiotensin-Aldosterone-System are conserved in zebrafish; (2) zebrafish has multiple paralogs of some RAAS components and results clarify their evolutionary relationships to human genes; (3) zebrafish and human RAAS genes are generally expressed in equivalent cell types; and (4) a specific type of enterocyte is newly identified as a focus of RAAS component expression, including the SARS-CoV-2 receptor. Because many components of the RAAS function in zebrafish as in humans, these investigations define the genes and cell types for investigating mechanisms leading to COVID-19 mechanisms in a zebrafish model.

### Zebrafish orthologs and co-orthologs of human RAAS components

Key conserved features include *ace2*, which encodes the zebrafish ortholog of the SARS-CoV-2 receptor, and *ace*, which encodes the zebrafish ortholog of the enzyme that produces Ang II, a peptide that can exacerbate symptoms of COVID-19 (hypertension, inflammation). Zebrafish preserves enzymes that metabolize Ang-related peptides in mammals, including Ace, Ace2, Enpep, and Anpep, as well as Ang II receptors Agtr1 and Agtr2. Zebrafish has orthologs of human genes encoding SLC6A19, which binds ACE2, and ADAM17, which creates soluble ACE2. Furthermore, zebrafish has an ortholog of *TMPRSS2*, which encodes an enzyme that activates coronavirus spike protein for binding ACE2 thus allowing infection. Zebrafish also has orthologs of genes encoding the ligand and receptors for the Apelin signaling system.

Zebrafish has a single ortholog of most RAAS genes but has duplicates of some. TGD duplicates include (1) *agtr1a* and *agtr1b*; (2) *anpepa* and *anpepb*; (3) the *slc6a19a* locus and *slc6a19b*; and (4) *aplnrla* and *aplnrlb*. Other duplicated RAAS genes are tandem duplicates, including (1) *anpep* and *anpepl*; (2) *anpepla.1* and *anpepla.2*; and (3) *slc6a19a.1* and *slc6a19a.2*. Significantly, the functions of both zebrafish co-orthologs must be considered when translating zebrafish science to human biology.

The RAAS/Apelin system also provides an example of ‘ohnologs gone missing’, in which one ohnolog arising in vertebrate genome duplication events is lost in either ray-finned or lobe-finned vertebrates but retained in the other taxon (Postlethwait, 2007); this situation happened for *Aplnrl* genes.

Zebrafish appears to lack two major RAAS components. Zebrafish and other ray-finned vertebrates lack an ortholog of the Ang1-7 receptor *MAS1* (Bader et al., 2014; Fournier et al., 2012; Hoffmann et al., 2018), a member of a large gene family characterized by gene duplication and chromosome rearrangements (Rinne et al., 2019). Another G-protein-coupled receptor may substitute, or Ang1-7 may not have a receptor in ray-finned vertebrates.

The second missing component is aldosterone. In tetrapods, this adrenal steroid causes the kidney to resorb sodium and secrete potassium and hydrogen ions, thereby regulating water balance and blood pressure. Teleosts, cartilaginous fishes, and jawless fishes lack aldosterone (Bridgham et al., 2006; Jiang et al., 1998; Nunez and Trant, 1999; Simpson and Wright, 1970), which emerged in tetrapods after evolution of the ability of Cyp11b1 to hydroxylate corticosterone (Bülow and Bernhardt, 2002; Jiang et al., 1998; Nonaka et al., 1995). Curiously, teleosts nevertheless have an ortholog of Nr3c2, the aldosterone (mineralocorticoid) receptor (Bridgham et al., 2006), which in fish is stimulated by either the aldosterone precursor 11-deoxycorticosterone or by cortisol (Kumai et al., 2014; Sturm et al., 2005). In the Atlas, *nr3c2* was expressed in embryonic periderm and larval gills, which help regulate salt balance at these developmental stages (Dymowska et al., 2012; Fu et al., 2010; Hoffmann et al., 2018; Rombough, 2007), suggesting that an ancestral role of Nr3c2 included salt and water balance even in the absence of aldosterone. Zebrafish mutants lacking active *nr3c2* responded poorly to a swirling stress test, but their response to hypotonic or hypertonic stress has not been reported (Faught and Vijayan, 2018). We also don't know whether zebrafish Angiotensin receptors stimulate the release of 11-deoxycorticosterone, cortisol, or another steroid.

The conservation of RAAS/apelin components makes zebrafish an appropriate model to investigate their roles in the mechanisms of COVID-19.

### Zebrafish express COVID-19-related RAAS genes in cell types as in humans

Results showed that, like humans, zebrafish liver cells express *Angiotensinogen*. In the Atlas, 5 dpf larvae had three types of hepatocytes, adding developmental and cellular precision to expression studies in adult zebrafish (Cheng et al., 2006). *Agt* expression in mammals relies on cortisol and inflammation (Brasier and Li, 1996; Demura et al., 2015). Cortisol binds the glucocorticoid receptor Nr3c1 and inflammation acts via interleukins and Tnfa to cause CCAAT-binding proteins like Cebpb and Cebpd to bind *Agt* enhancer elements. The co-expression of *agt*, *cebpb*, *cebpd*, and to some extent *nr3c1* shows that gene expression is as expected if the initiation of the RAAS cascade is similar in humans and zebrafish.

Angiotensinogen is cleaved to form Ang I by Renin, which adult kidney juxtaglomerular cells secrete in mammals and zebrafish (Gomez et al., 1988; Hoshijima and Hirose, 2007; Jones et al., 1990; Liang et al., 2004; Rider et al., 2015). Renin-expressing cells in fetal mammals first appear in several tissues and organs, but predominantly in the adrenal (Gomez et al., 1988; Sequeira Lopez et al., 2004). Zebrafish may share this developmental pattern because one of the three *renin-*expressing cells in the Atlas was an interrenal cell (Yan et al., 2020b) (the fish adrenal equivalent; (Liang et al., 2004), although larger sample sizes are needed to draw firm conclusions.

Ace, which cleaves Ang I to Ang II, was co-expressed with several other RAAS-components almost exclusively in a subtype of enterocytes as judged by their co-expression of absorptive enterocyte markers *alpi.2, vil1*, and *slc34a2a* (Makky et al., 2007; Wallace et al., 2005; Yang et al., 2012). Larval enterocytes expressed not only *ace*, but also *ace2* and *tmprss2*, the human ortholog of which activates the coronavirus spike protein, thereby stimulating infection, and expresses in the human digestive system (Fagerberg et al., 2014; Hoffmann et al., 2020; Millet and Whittaker, 2015; Walls et al., 2020). The human protease CTSL cleaves the spike protein of SARS-CoV (Simmons et al., 2005) and we show that in zebrafish, *ctsla* is also strongly expressed in *ace2*-expressing cells.

Zebrafish enterocytes also encode other enzymes that cleave Ang peptides in mammals (Enpep, Anpepa, Anpepb, and Dpp4). Expression of these genes in zebrafish enterocytes suggests that their counterpart in human could be important for coronavirus infections because: (1) ANPEP is a receptor for the human common cold coronavirus HCoV-229E (Fehr and Perlman, 2015); (2) DPP4 is the receptor for MERS-CoV, the causative agent of the Middle East Respiratory Syndrome (Raj et al., 2013); and (3) ACE2 is the receptor for SARS-CoV-2. The zebrafish scRNA-seq results suggest that the corresponding human cell type may contribute to digestive tract symptoms experienced by many COVID-19 patients (Guan et al., 2020; New York Department of Health, 2020).

Enterocytes also express zebrafish *slc6a19* genes. In human, SLC6A19 binds ACE2 to form the SARS-CoV-19 receptor (Yan et al., 2020a). Co-expression of *ace2* and *slc6a19* in zebrafish suggests functions shared with human. SLC6A20, which our genomic analysis showed is a likely tandem duplication-derived paralog of SLC6A19, also interacts functionally with ACE2 (Kristensen et al., 2011; Vuille-dit-Bille et al., 2015). Significantly, *SLC6A20* is at the peak of the strongest of two genome-wide association study loci for undesirable COVID-19 outcomes (Ellinghaus et al., 2020). We suggest that genetic variants near *SLC6A20* affect the severity of COVID-19 symptoms due to interactions of SLC6A20 with ACE2.

Angiotensin peptides bind to Agtr1 and Agtr2 receptors on vascular cells to help regulate vasoconstriction, and on the adrenal to stimulate secretion of aldosterone, leading to salt and water retention, which may be related to comorbidities of hypertension and obesity-related kidney damage (Fyhrquist and Saijonmaa, 2008). Correspondingly, our zebrafish scRNA-seq analysis showed that endothelial cells express *agtr1b* and confirmed *agtr2* expression in vascular cells (Wong et al., 2009).

The conserved expression of zebrafish RAAS-related genes shown here supports the contention that RAAS regulation and function are similar in zebrafish and human and the novel finding of the nexus of RAAS component expression in specific enterocytes focuses future COVID-19-related research on these cells.

### The zebrafish RAAS functions like that of mammals

Hypertension, obesity, and diabetes are COVID-19 comorbidities (Richardson et al., 2020); they relate to the role of the RAAS in salt and water balance, vessel function, and inflammation; and they lie downstream of Ang II (Cabandugama et al., 2017; Fyhrquist and Saijonmaa, 2008). In humans, adrenal-derived glucocorticoids and inflammation induce Angiotensinogen (Brasier and Li, 1996; Stockhammer et al., 2010); inflammation also upregulates *agt* in zebrafish (Stockhammer et al., 2010), suggesting conserved regulatory mechanisms. The role of cortisol in *agt* regulation in zebrafish remains to be investigated.

As in human, *Renin* transcription in zebrafish is regulated by plasma salt concentration (Hoshijima and Hirose, 2007; Rider et al., 2015). In larval zebrafish, levels of Ang II downstream of Renin increase after exposure to acidic or ion-poor water (Kumai et al., 2014), similar to the response of Ang II in humans with high levels of circulating sodium (Crowley and Coffman, 2012). Zebrafish *ren* mutants are normally adult viable but have enlarged swim bladders, as expected from aberrant water balance, and dramatically more Renin cells in the kidney, suggesting regulation by a negative feedback loop (Mullins et al., 2019). Treating larval zebrafish with Ang I or Ang II caused animals to accumulate Na^+^ (Kumai et al., 2014), showing that zebrafish larvae respond similarly to mammals. In mammals, Ang II acts on sodium uptake when aldosterone binds to aldosterone receptor, but fish have no aldosterone, so although input and outcome are similar, the steroid must differ, likely either cortisol or 11-deoxycorticosterone in fish (Sturm et al., 2005). Recent investigations in zebrafish with knockout mutations in the glucocorticoid receptor (*nr3a1*) and the mineralocorticoid receptor (*nr3a2*) genes promise to clarify these issues (Faught and Vijayan, 2018, 2020; Ziv et al., 2013).

The zebrafish RAAS is pharmacologically similar to that of mammals. The Ace inhibitor lisinopril blocks effects of Ang I on sodium uptake in zebrafish, as predicted if Ace were required to convert Ang I to Ang II in zebrafish as in mammals (Kumai et al., 2014). Zebrafish cultured from 24 hpf to 96 dpf in water containing the Ace inhibitor captopril do not differ in survival from controls and neither do fish in water with 5% of the normal salt concentration (Rider et al., 2015), but the combination of captopril and low ionic strength water reduces survival by about 95% and increases Renin expression (Rider et al., 2015). Ace inhibition by captopril also upregulates *ren* in adult and larval zebrafish (Hoshijima and Hirose, 2007; Rider et al., 2017, 2015) and affects cardiovascular function (Margiotta-Casaluci et al., 2019). Captopril also counters the effect of SARS-CoV-2 spike protein binding domain on heart rate in larval zebrafish (Kraus et al., 2020 preprint). The Ace inhibitor blood pressure medication enalapril causes intraocular blood vessels to dilate in zebrafish (Kitambi et al., 2009) as expected from conserved functions. The fact that Ace inhibitors have similar effects in humans and zebrafish supports physiological conservation of the Ace system (Prokop et al., 2015).

Drugs that block the Ang II receptor Agtr1 also show conserved RAAS function. The selective Agtr1 antagonist telmisartan blocks sodium uptake in zebrafish cultured in low ionic strength water as expected if Ang II acts on sodium retention by binding to Agtr1 (Kumai et al., 2014), even though fish lack aldosterone. The high blood pressure drug fimasartan, an Ang II receptor antagonist, also ameliorates zebrafish models of heart failure, normalizing the expression of atrial natriuretic peptide, reducing cell death around the heart, and improving blood flow (Quan et al., 2020). These results show that Agtr1 structure and function are conserved in human and zebrafish.

### Conclusions

These discussions show that zebrafish has nearly all RAAS and Apelin signaling components, distinguish between previously confusing zebrafish orthologs and paralogs of human RAAS-related genes, identify for the first time a specific cell type in which many critical zebrafish RAAS components are expressed, and first identify these special cells as enterocytes. Coupled with data from the literature showing that the RAAS functions in similar ways in zebrafish and human and that RAAS-related drugs tend to act in zebrafish as they do in humans, our analysis shows zebrafish to be a valuable model for interrogating roles of the RAAS in COVID-19 comorbidities. Given the exquisite imaging that zebrafish embryos and larvae provide and the ease of genome editing, zebrafish offers a highly useful system for research into the mechanisms of COVID-19 pathologies and comorbidities. Knock-in of the human *ACE2* gene into zebrafish coupled with challenges with synthetic SARS-CoV-2 spike protein (Kraus et al., 2020 preprint) will illuminate both COVID-19 comorbidities and factors affecting disease severity. The use of appropriate genotypes of zebrafish in a small molecule screen could help identify small drug-like molecules that inhibit the interaction of the SARS-CoV-2 spike protein with the human receptor or the inflammation that accompanies severe COVID-19 outcomes.

## MATERIALS AND METHODS

### Phylogenies and conserved syntenies

To identify RAAS-related genes, we utilized phylogenetic analysis based on phylogenetic distance, tree structure, and gene duplications embedded in ComparaTree (Flicek et al., 2010). To verify orthologs and co-orthologs, we analyzed conserved syntenies using the Synteny Database (Catchen et al., 2009). Nomenclature rules for genes and proteins in human, mouse, and zebrafish use conventions described at the Zebrafish Information Network (ZFIN, https://wiki.zfin.org/display/general/ZFIN+Zebrafish+Nomenclature+Conventions). For non-human sarcopterygians, we use mouse conventions and for actinopterygians, we use zebrafish conventions.

### *In situ* hybridizations

*In situ* hybridization was performed as previously described (Rodriguez-Mari et al., 2005) using probes made from the following primers and cDNA: *ace2* (ENSDARG00000016918) using a 1200 bp fragment extending from partial exon-9 to exon-18 [primers: forward (F)- CTGTTGGAGAGATCATGTCGCTTTCT and reverse (R)- TGTCTTCCTCAAAGGCTTTGTTCACT]; *slc6a19a.1* (ENSDARG00000018621) using a 609-bp fragment extending from partial exon-4 to exon-8 (primers F- TGGATTATTTCTGGTACCGGGAGACT and R- ATGATGTCTGGTCTGCTGATGTTGAG); *slc6a19.a.2* (ENSDARG00000091560) using a 1050-bp fragment including partial exons 3-10 (primers F- TTACCCTGGAGCCAGTGTCCTATTAA and R-GATGATCAGCAGAGGAATGGATACGG); *slc6a19b* (ENSDARG00000056719) using a 1100-bp fragment extending from the exon-2/3 border to exon-10 (F- GAGTTGGCATTGCATCTATGTGTGTG and R-GTTTCCTGAGCCCTGGACAAATATCA).

### Single cell transcriptomics

The zebrafish single cell transcriptomic Atlas (Farnsworth et al., 2020) provided single-cell expression patterns of zebrafish RAAS and Alpn-signaling components. Animals: zebrafish (*D. rerio*) were reared using standard husbandry (Westerfield, 2007). Strains used were *Tg(olig2:GFP)vu12* for two samples at 1 and 5 dpf and one sample at 2 dpf, and *Tg(elavl3:GCaMP6s)* for one sample at 2 dpf. Embryos were dissociated as described (Farnsworth et al., 2020). Fifteen animals of mixed but unknown sex were used for each of the six batches. Work was conducted under the approval of the University of Oregon IACUC, protocol number 18-31. Reagents: dissociated cells were prepared for scRNA-seq using the 10X Chromium platform v.2 chemistry. cDNA libraries were amplified by 15 PCR cycles and sequenced on Illumina Hi-seq or Next-seq. Statistics: sequencing data were analyzed by Cell Ranger version 2.2.0 (Zheng et al., 2017) and Seurat (Satija et al., 2015). For further details, see (Farnsworth et al., 2020) and https://www.adammillerlab.com/. Annotation: analysis of differential gene expression used Seurat's FindAllMarkers function and Wilcoxon rank sum test (Satija et al., 2015). The 16 most differentially expressed genes in each cluster were defined as those with the highest ratio of percent of cells expressing the gene in a cluster over the percent of cells expressing the gene in all other cells in the Atlas. *In situ* hybridization patterns for genes found in ZFIN (https://zfin.org/action/marker/search) facilitated annotation of the most likely cell type for each cluster. Data availability: scRNA-seq data are publicly available at https://cells.ucsc.edu/?ds=zebrafish-dev.

## Supplementary Material

Supplementary information
