# Supplementary material for: The SARS-CoV-2 receptor and other key components of the Renin-Angiotensin-Aldosterone System related to COVID-19 are expressed in enterocytes in larval zebrafish
Source: Biol Open. 2021 Mar 23;10(3):bio058172. doi: 10.1242/bio.058172 (PMC8015242; doi:10.1242/bio.058172)
Supplement: Supplementary information [file biolopen-10-058172-s1.pdf]

*Figure S1. An overview of the mammalian RAAS.*

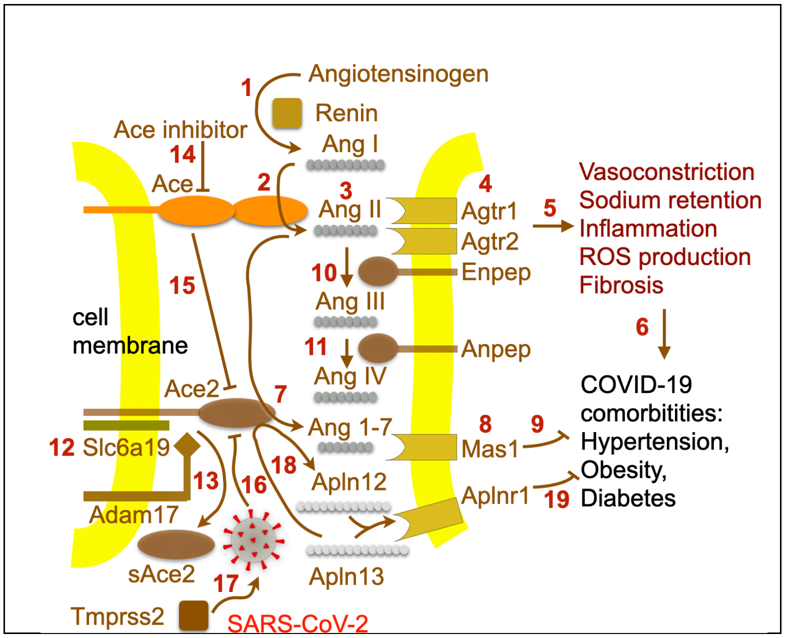


In the RAAS (Renin-Angiotensin-Aldosterone System), the kidney enzyme Renin cleaves the liver protein Angiotensinogen (Agt) into the 10-amino acid peptide hormone Angiotensin I (Ang I, Step 1). Angiotensin I converting enzyme (Ace, Step 2), a dipeptidyl carboxypeptidase paralog of Ace2, converts Ang I to the 8-amino acid peptide Ang II (Step 3), which can bind to Angiotensin receptors (Agtr1 and Agtr2, Step 4), thus promoting vasoconstriction, salt and water retention by the kidney, inflammation, and production of harmful reactive oxygen species (ROS) (Step 5) (Fyhrquist and Saijonmaa, 2008). Ang II normally helps maintain blood pressure after a sudden drop in blood flow to the kidney, or reduced sodium excretion, which could lead to hypotension and fainting if a person is dehydrated or stands up too quickly. At least some of these features appear to contribute to COVID-19 comorbidities, including hypertension, diabetes, cardiovascular disease, and cerebrovascular disease (Step 6) (Wang et al., 2020). Ang II levels decrease when Angiotensin I converting enzyme 2 (Ace2) removes an amino acid thus producing Ang1-7 (Step 7), which binds to the G protein-coupled receptor Mas1 (Step 8) to promote vasodilation and diuresis (Step 9), thus countering many of the effects of Ang II (Fyhrquist and Saijonmaa, 2008). Ang II can also be catabolized by the enzymes Enpep and Anpep to other products (Steps 10 and 11) (Holmes et al., 2017). Ace2 also acts as a protein chaperone for the neutral amino acid transporter Slc6a19 (Step 12) (Camargo et al., 2009). Ace2 can appear in plasma after cleavage by the enzyme Adam17 (Step 13) (Lambert et al., 2005). The significance of soluble Ace2 is not yet well understood (Jiang et al., 2014), although it has recently been suggested that its higher level in females may help them fight COVID-19 better than men (Ciaglia et al., 2020) and to provide a potential therapeutic approach (Batlle et al., 2020).

Ace inhibitor drugs (Step 14) bind to the active site of Ace but not Ace2 (Rice et al., 2004), which decreases Ang II production and thus dampens hypertension and other COVID-19 underlying conditions (Messerli et al., 2018; Natesh et al., 2004; Sommerstein et al., 2020). Drugs that act as Ang II receptor blockers reduce the efficacy of Ang II and thus also improve cardiovascular health. About 19% of people hospitalized for COVID-19 have been taking one of these drugs due their beneficial effects on what turn out to be COVID-19 comorbidities, especially hypertension (Richardson et al., 2020). We do not yet know fully the physiological differences between Ace inhibitors and Ang II receptor blockers (Vaduganathan et al., 2020). It is also not fully known whether Ace inhibitors or Angiotensin receptor blockers benefit or harm COVID-19 patients (Sommerstein et al., 2020; Vaduganathan et al., 2020).

Ace inhibitors have effects in addition to decreasing Ang II levels; they cause an increase of Ace2 expression in the heart, intestine and other organs (Ferrario et al., 2005; Kuster et al., 2020; Vuille-dit-Bille et al., 2015). This finding suggests that Ace activity inhibits *Ace2* transcription (Step 15). Surprisingly, COVID-19 comorbidities themselves, including hypertension, myocardial infarction, and diabetes, also upregulate Ace2 expression (Burrell et al., 2005; Roca-Ho et al., 2017; Uri et al.,

Biology Open • Supplementary information

2016; Uri et al., 2014; Walters et al., 2017). The mechanisms by which Ace activity and COVID-19 comorbidities regulate Ace2 expression, however, remain unknown.

The RAAS may contribute to COVID-19 not only because Ang II promotes comorbidities, but also because SARS-CoV-2 enters cells by binding to Ace2 (Step 16) near its active site (Hoffmann et al., 2020; Yan et al., 2020). The transmembrane serine protease Tmprss2 (Step 17) cleaves off a portion of the SARS-CoV-2 spike protein, thus activating it for binding to Ace2 and allowing the virus to enter cells (Hoffmann et al., 2020; Millet and Whittaker, 2015; Walls et al., 2020). The binding of SARS-CoV-2 to Ace2 inhibits Ace2 activity (Kuba et al., 2005), which would decrease the destruction of Ang II (Step 7), thereby amplifying levels of Ang II and leading to more intense COVID-19 morbidities (Steps 4-6). The notion that RAAS activation leads to COVID-19 comorbidities is supported by knockout mice that lack Angiotensinogen or its receptors, which show less obesity, less insulin resistance, and less hypertension (Massiera et al., 2001; Yvan-Charvet et al., 2005).

Ace2 also interacts with the Apelin-Apelin Receptor system. Apelin (Apln, Step 18) peptides of various length cause vasodilation, increase heart muscle contractility, angiogenesis, fluid homeostasis, and contribute to energy metabolism regulation; thus, Apelin tends to counter the effects of Ang II (De Mota et al., 2004; Dray et al., 2008; Kasai et al., 2004; Szokodi et al., 2002). Ace2 can cleave Apln-13 to Apln-12, and both peptides appear to be physiologically active (Yang et al., 2017). Apelin is a positive regulator of Ace2, so that decreasing Apelin downregulates Ace2 expression, and hence reduces Ang II degradation, thereby increasing Ang II levels (Sato et al., 2013); thus, Apln and its receptors (one of which was formerly called Agtrl1a, Angiotensin receptor-like 1a) may be relevant to COVID-19.

Figure S2. *Angiotensinogen* phylogeny and expression. A. Phylogenetic tree ENSGT00890000139531 from Ensembl showing a single zebrafish ortholog of mammalian *Agt*. B. Expression of *agt* in liver clusters c55, c121, and c217; expression scale at right. C. Expression of potential regulator *nr3c1,* encoding the cortisol receptor. Cell colors reflect *agt*-expression and *nr3c1*-expressing cells are circled. Periderm cells, basal skin cells, ionocytes, fast skeletal muscle, photoreceptors, and vascular endothelium expressed *nr3c1* at higher levels than these in the liver (see Supplementary Fig. S10E. C. Expression of the *agt*-regulator *cebpd* in *agt*-expressing hepatocytes (see also Fig. S10F).

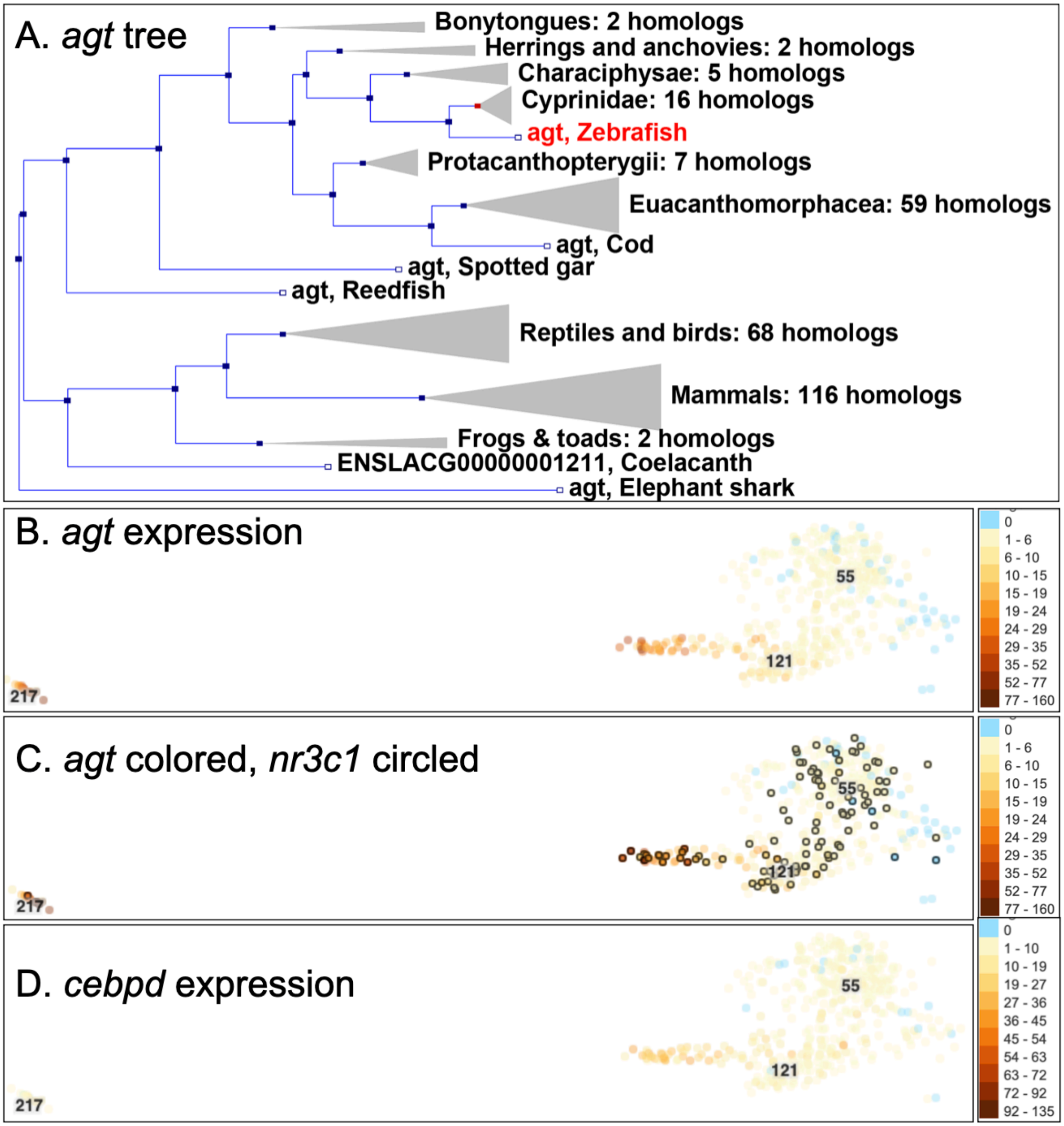


Biology Open • Supplementary information

Biology Open (2021): doi:10.1242/bio.058172 Supplementary information

**Figure S3. Renin genomics**. A. Conserved syntenies confirm orthology of zebrafish *ren* to human *REN*. B, C. *ren* (orange cell) in c87, which contains precursors to the interrenal, the fish equivalent of the adrenal cortex, which expresses *Ren* in the fetus. Interrenal cells expressing cortisol-synthesis genes *star* (B) and *hsd3b1* (C ) are circled. D. Zebrafish and other teleosts have one *renin* gene (Tree ENSGT00940000157898). E. Origin of protease-encoding genes *RENIN* (Hsa1)*, CTSD* (Hsa11)*,* and *NAPSA* (Hsa19) by whole genome duplication. Orthologs and paralogs of Hsa11 genes plotted on rows corresponding to their chromosome directly above or below the location of the Hsa11 gene. Hsa12 lacks a *CTSD* paralog.

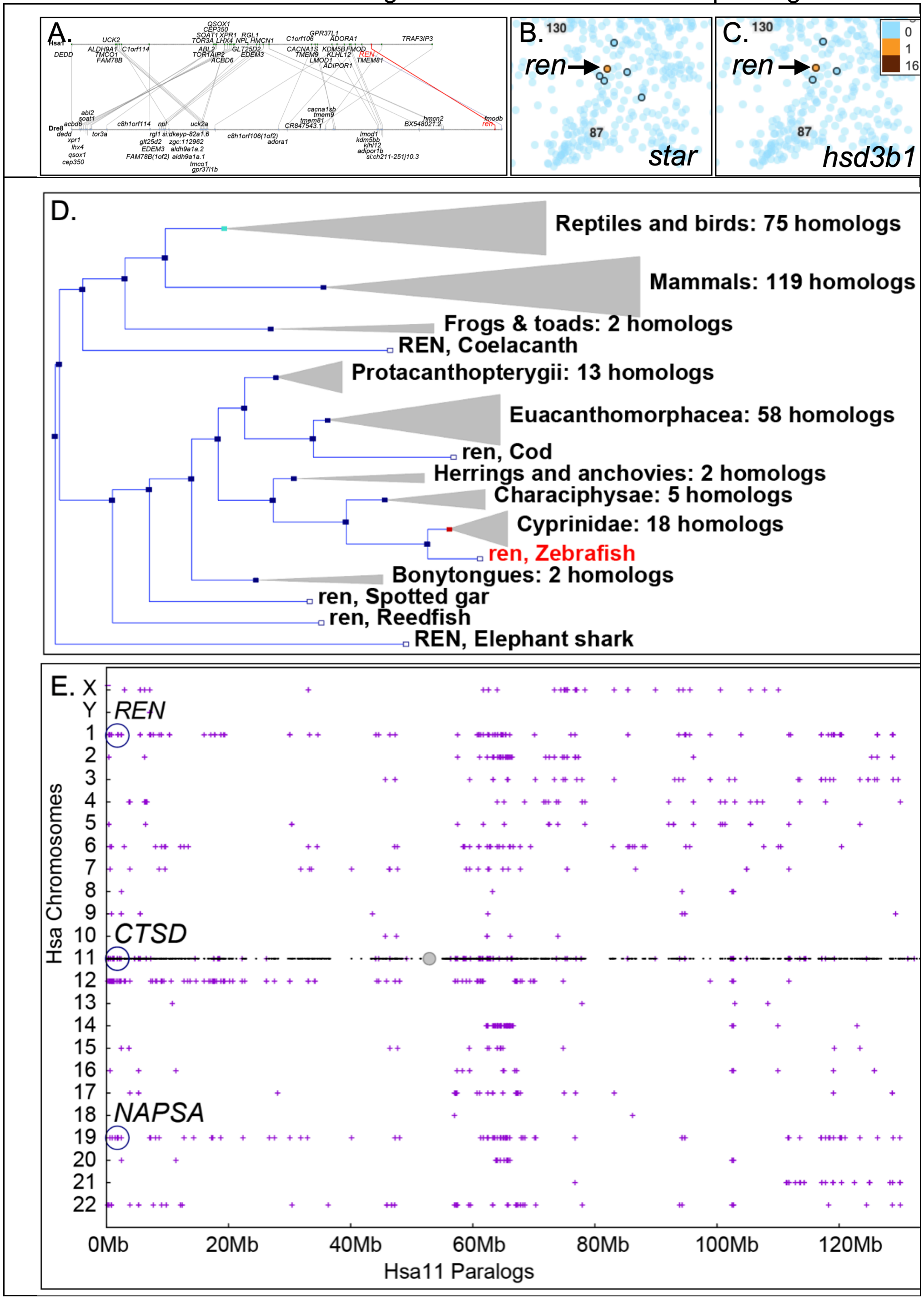


Biology Open • Supplementary information

**Figure S4. Genomics of *ace2* and *enpep*.** A. Gene tree ENSGT00940000158077 shows that zebrafish has one *ace2* (ENSDARG00000016918) ortholog of the human *ACE2* gene (ENSG00000130234). B. Gene tree ENSGT00940000156946 showing one *enpep* ortholog in zebrafish. C. Conserved syntenies verify the orthology of zebrafish *enpep* (ENSDARG00000057064) to human *ENPEP* (ENSG00000138792).

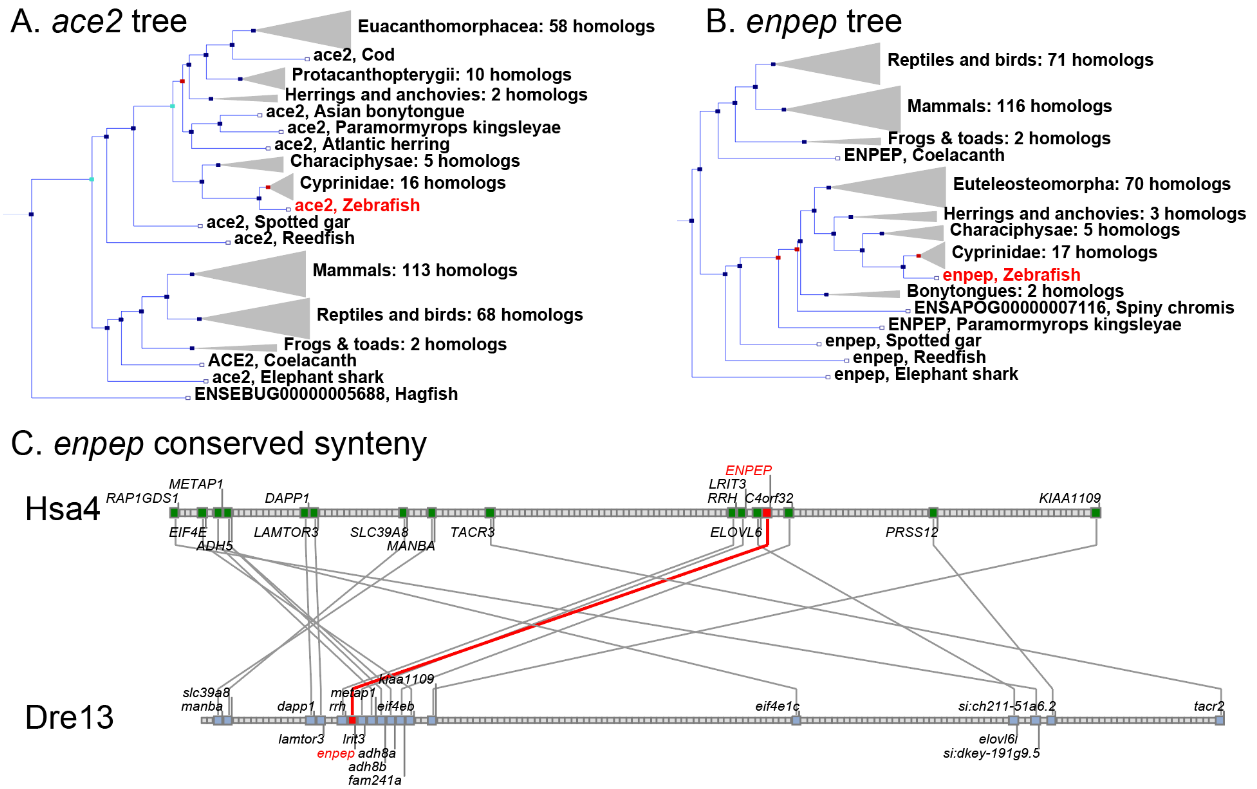


Biology Open • Supplementary information

**Figure S5. What is the evolutionary relationship between the human *ENPEP* gene and its five zebrafish paralogs?** A. Conserved synteny analysis showed that zebrafish chromosomes Dre18, Dre25, and Dre7 have orthologs on human chromosome Hsa15 surrounding *ANPEP*. Two *ANPEP*-related genes and their surroundings on Dre7 have paralogs (ohnologs) from the teleost genome duplication event on either Dre18 or Dre25, but not both (other than these two *ANPEP*-related genes). This result is consistent with a previously identified translocation event in the zebrafish lineage (Nakatani and McLysaght, 2017) that separated tandemly duplicated *anpep* paralogs and argues that the two *anpep*-related genes on Dre7 and the three on Dre18+Dre25 derive from the teleost genome duplication. According to zebrafish nomenclature conventions, one pair should represent ‘a’ copies and the other pair ‘b’ copies. B. Comparatree ENSGT00940000154876 contains the human *ANPEP* gene (ENSG00000166825) and branches that are consistent with the three zebrafish genes in the tree being co-orthologs of the human gene. The two adjacent *ANPEP*-related genes on Dre25 (*anpepb.1* (ENSDARG00000103878) and *anpepb.2* (ENSDARG00000097285)) are sisters in the tree and adjacent on the chromosome, so they resulted from a tandem duplication event. These tandem duplicates in the *anpepb* clade form a sister to a clade containing *anpepa* (ENSDARG00000036809) on Dre7, as expected for paralogs (ohnologs) from the teleost genome duplication. C. A separate Comparatree (ENSGT00940000164605) contains *anpeplb* (ENSDARG00000041083) on Dre18 and *anpepla* (ENSDARG00000089706) on Dre7. These genes fall as sisters, as expected from TGD duplicates, and they are less related to *ANPEP* or the other three *anpep*-related genes in the tree in panel C. The two genes *anpepa* and *anpepla* on Dre7 are adjacent and in the same orientation. The finding that *anpepa* and the *anpepb* genes are more closely related phylogenetically to the human *ANPEP* gene than are the *anpepl* genes suggests that the tandem duplication to give *anpep* and *anpepl* occurred before the divergence of the zebrafish and human lineages but that *anpepl* was lost in stem lobe-finned vertebrates and thus is not in humans. D. A dot plot represents Hsa15 genes in order along the horizontal axis and directly above them, their orthologs or paralogs on zebrafish chromosomes. The plot shows the location of *ANPEP* on Hsa15 and reveals that Dre7, Dre18, and Dre25 are co-orthologous to Hsa15 over the distal 60Mb of the chromosome, consistent with Dre7 and (Dre18+Dre25) arising as duplicates from the TGD followed by a previously identified translocation (Nakatani and McLysaght, 2017) that separated Dre18 from Dre25 with *anpeplb* on one of the chromosomes and *anpepb* on the other. Supplementary Figure S6 shows a model for *Anpep* gene origins.

Biology Open • Supplementary information

Supplementary Figure S5A.

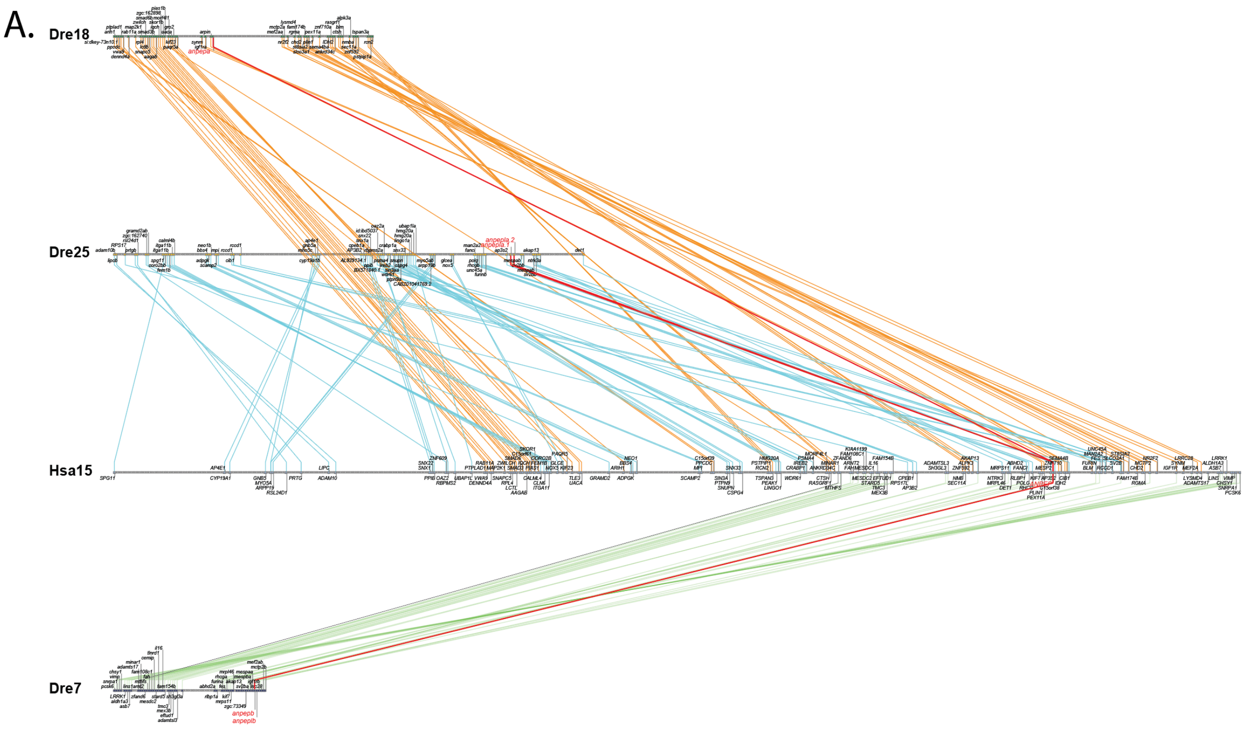

Supplementary Figure S5B-D.

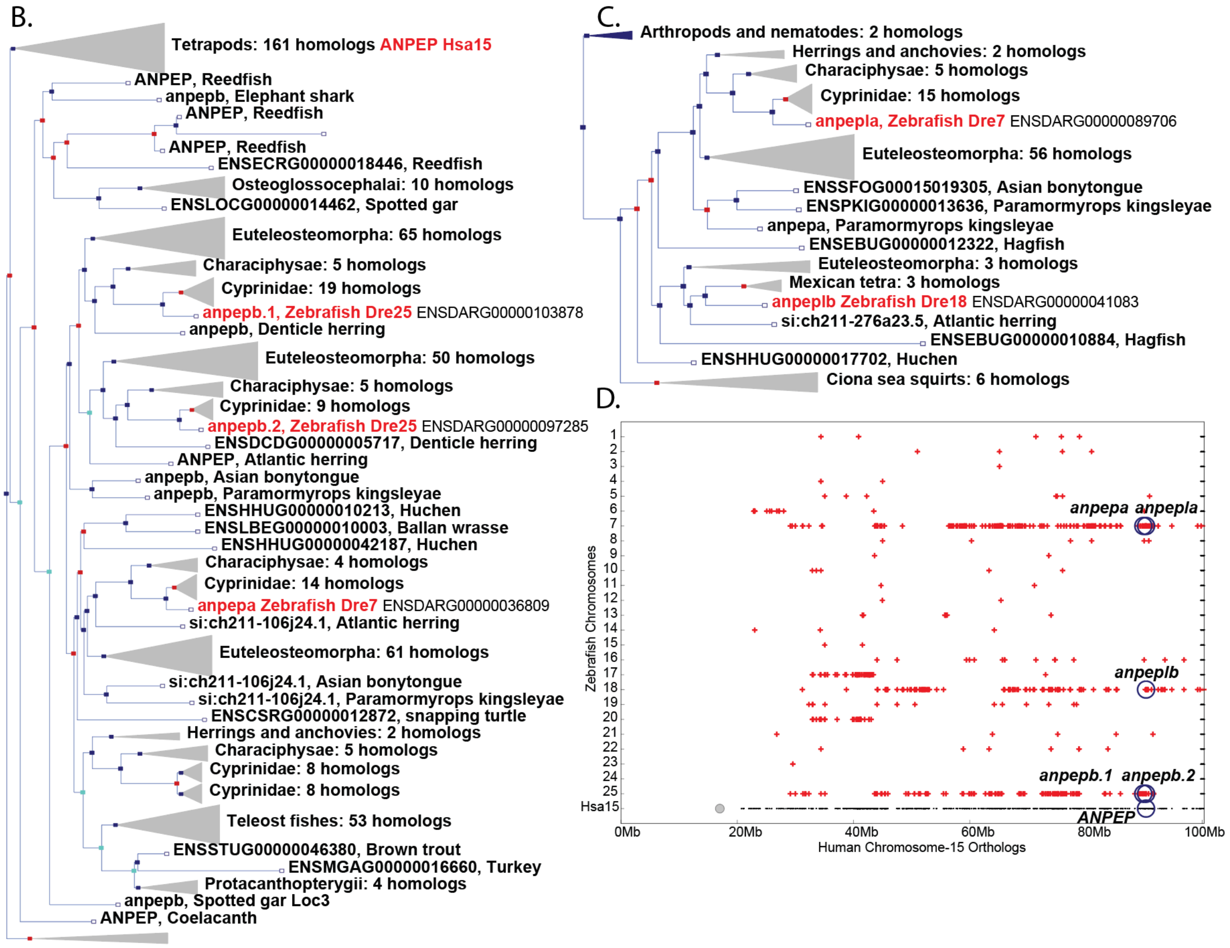


Biology Open • Supplementary information

**Figure S6. A model for the origin of the five zebrafish genes related to the single human *ANPEP* gene**. Trees and conserved syntenies (Supplemental Fig. S5) suggest that an original *anpep* gene was tandemly duplicated to give *anpep* and *anpepl* followed by loss of *anpepl* in lobe-finned vertebrates, leaving *ANPEP* (ENSG00000166825) in humans. The tandem duplication to form *anpep* and *anpepl* likely occurred before the divergence of ray-finned and lobe-finned vertebrates because *ANPEP* is substantially more closely related phylogenetically to *anpepa* and *anpepb* than to *anpepla* and *anpeplb* (Fig. S5 B, C). In the ray-finned lineage, the teleost genome duplication produced duplicates of both *anpep* and *anpepl*. The 'a' copies of both genes (*anpepa* (ENSDARG00000036809) and *anpepla* (ENSDARG00000089706)) are on zebrafish chromosome Dre7, but the 'b' copies of *anpeplb* and *anpepb* were separated by a chromosome fission event leaving *anpeplb* (ENSDARG00000041083) on Dre18 and *anpeplb* on the precursor of Dre25. Finally, *anpeplb* experienced a tandem duplication, giving *anpepb.1* (ENSDARG00000103878) and *anpepb.2* (ENSDARG00000097285).

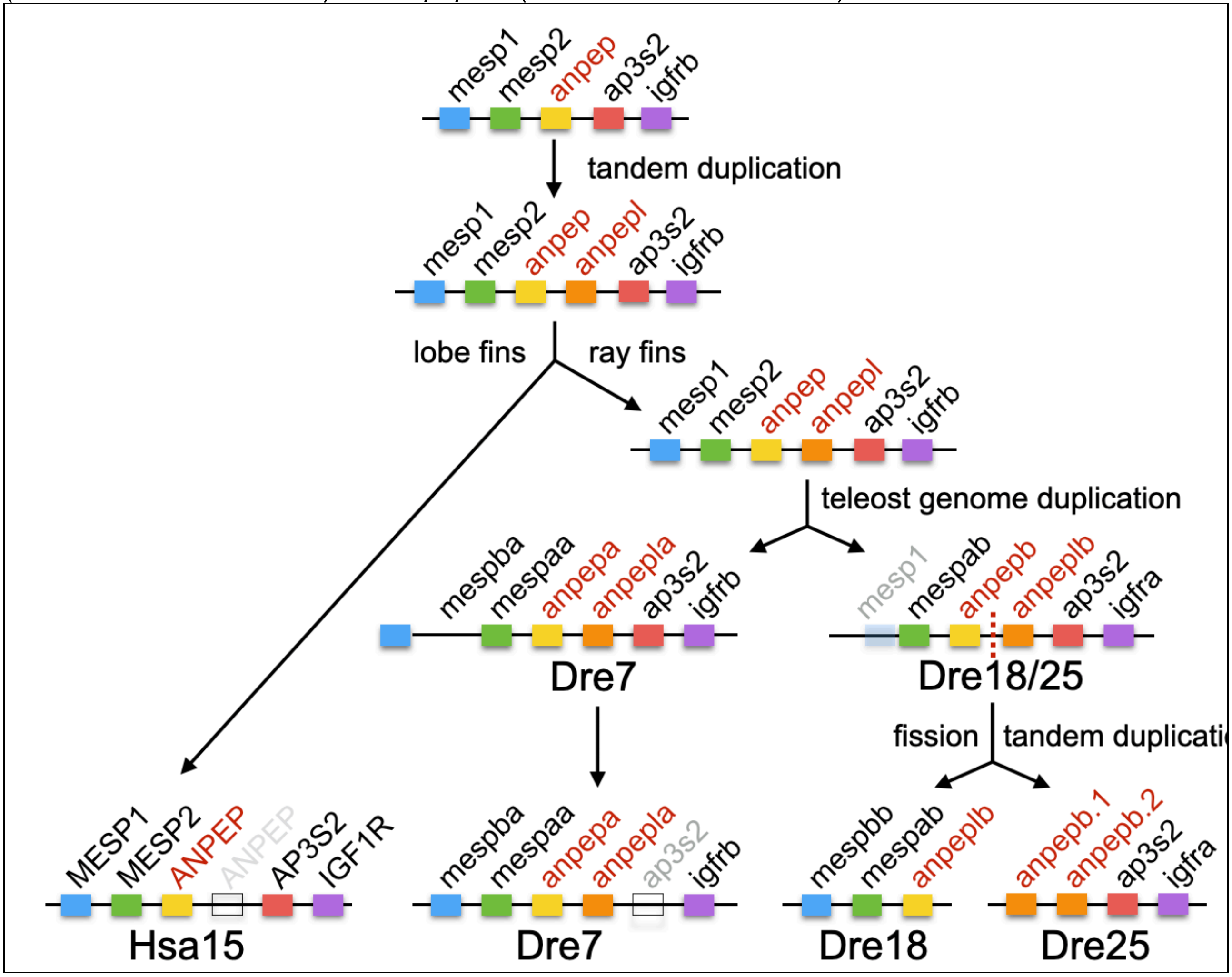


Biology Open • Supplementary information

Figure S7. Conserved syntenies for the human gene *DPP4* (ENSG00000197635) and the zebrafish gene *dpp4* (ENSDARG00000079420), confirming orthologies.

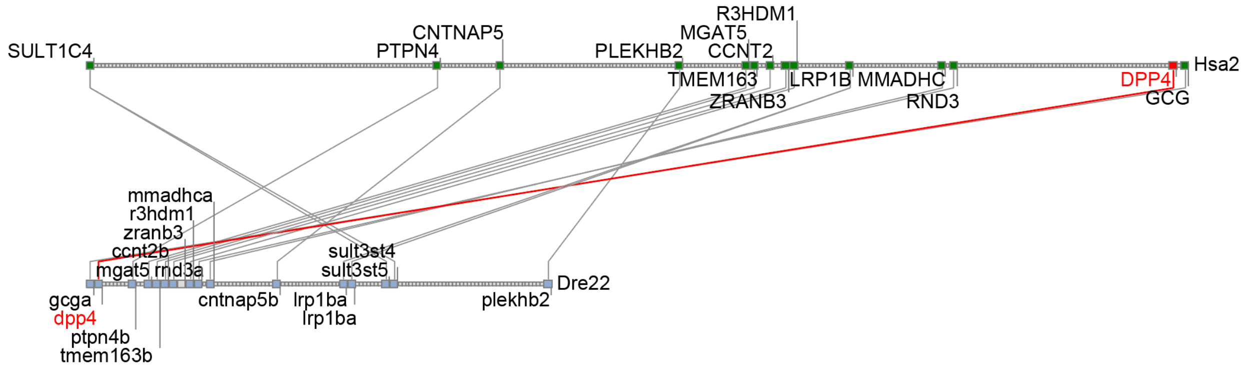


Biology Open • Supplementary information

**Figure S8. Phylogenetic tree for *SLC6A19*-related genes**. Phylogenetics (gene tree ENSGT00940000154896) shows that human *SLC6A19* (ENSG00000174358) has three co-orthologs in zebrafish. The zebrafish *slc6a19a.1* (ENSDARG00000018621) and *slc6a19.2* (ENSDARG00000091560) genes are tandem duplicates on chromosome Dre19 linked to the *hoxaa* cluster and occupy sister clades in the tree, while the clade containing *slc6a19b* (ENSDARG00000056719) on Dre16 linked to the *hoxab* cluster diverges as a sister to the *slc6a19a.1+slc6a19.2* clade. This tree supports the conclusion that *slc6a19a* and *slc6a19b* arose in the teleost genome duplication followed by a tandem duplication of *slc6a19a* to produce *slc6a19a.1* and *slc6a19.2*.

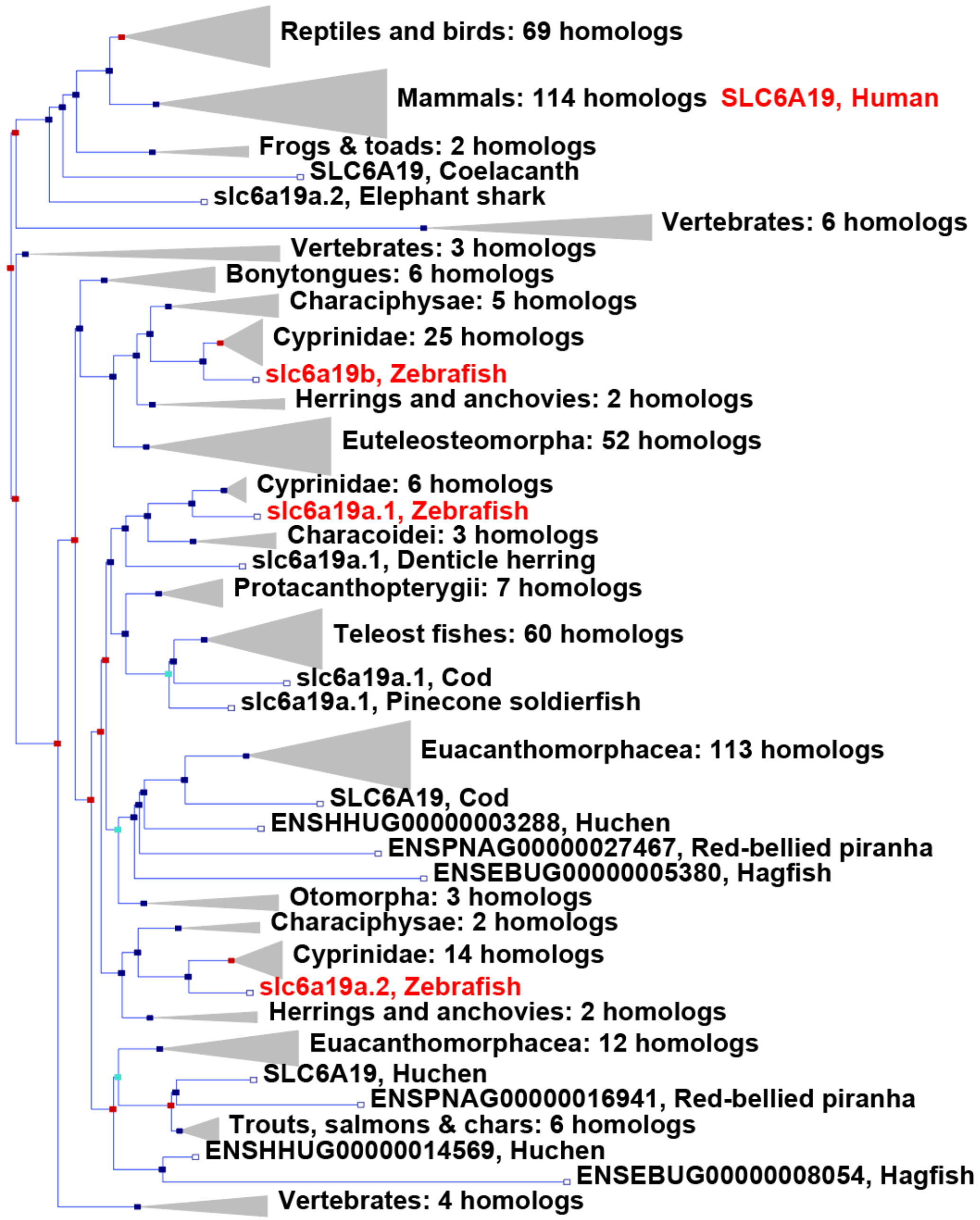


Biology Open • Supplementary information

**Figure S9. Phylogenetic tree and conserved synteny for *apln*.** A. The phylogenetic tree ENSGT00390000014020 shows that zebrafish has a single ortholog of *APLN* (ENSG00000171388). B. Conserved syntenies show that *apln* (ENSDARG00000053279) and *APLN* occupy orthologous chromosome segments.

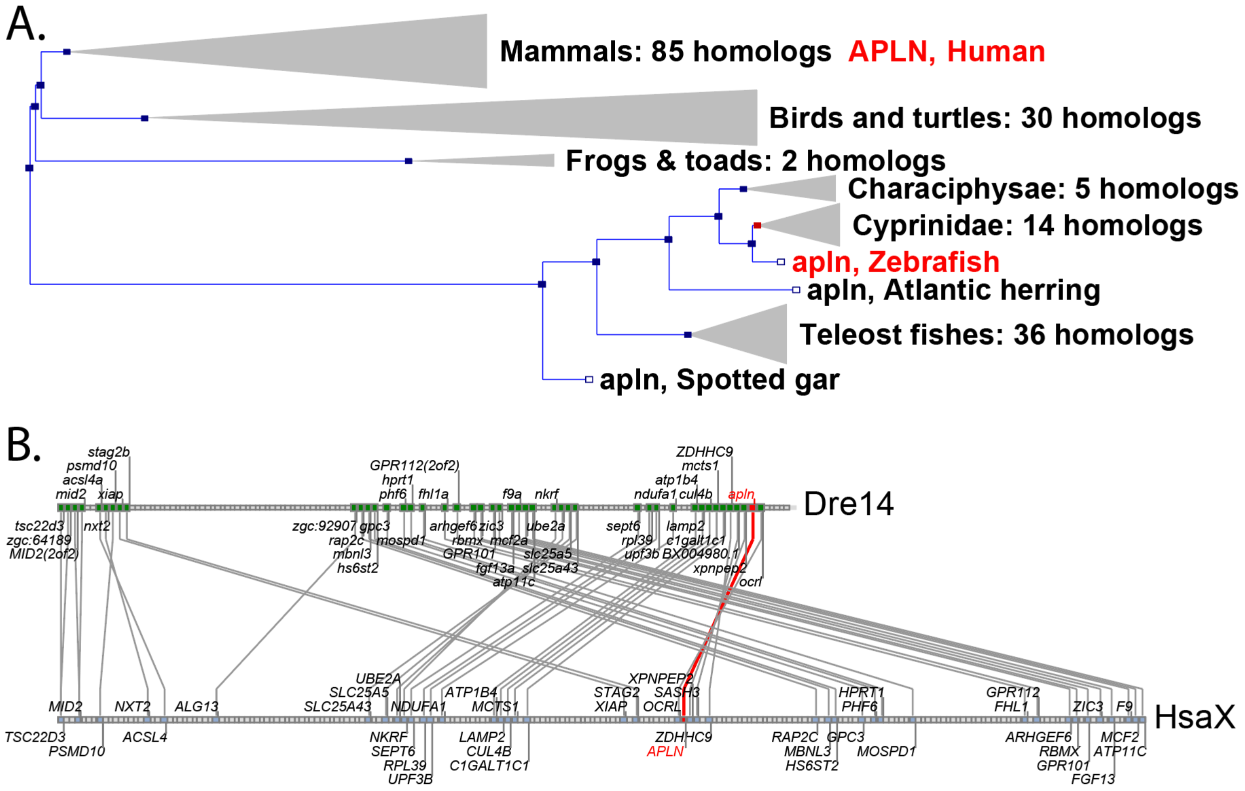


Biology Open • Supplementary information

Figure S10. Genes discussed in the text are shown in the context of the entire dataset. Original data available at: https://cells.ucsc.edu/?ds=zebrafish-dev.

## A. Colored by cluster.

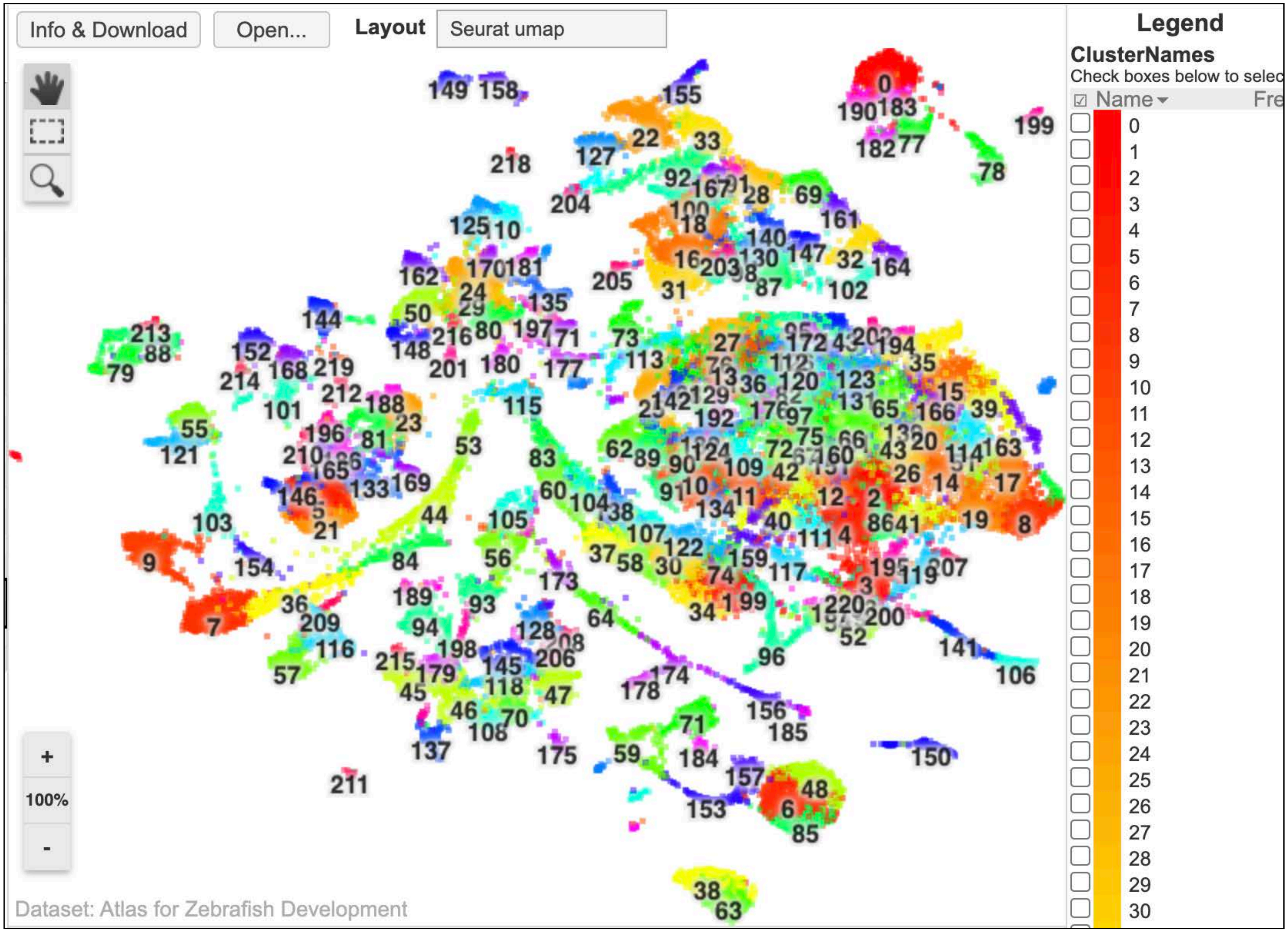


Biology Open • Supplementary information

B. Colored by age (1, 2, and 5 days post fertilization) and replicate (two replicates for each age).

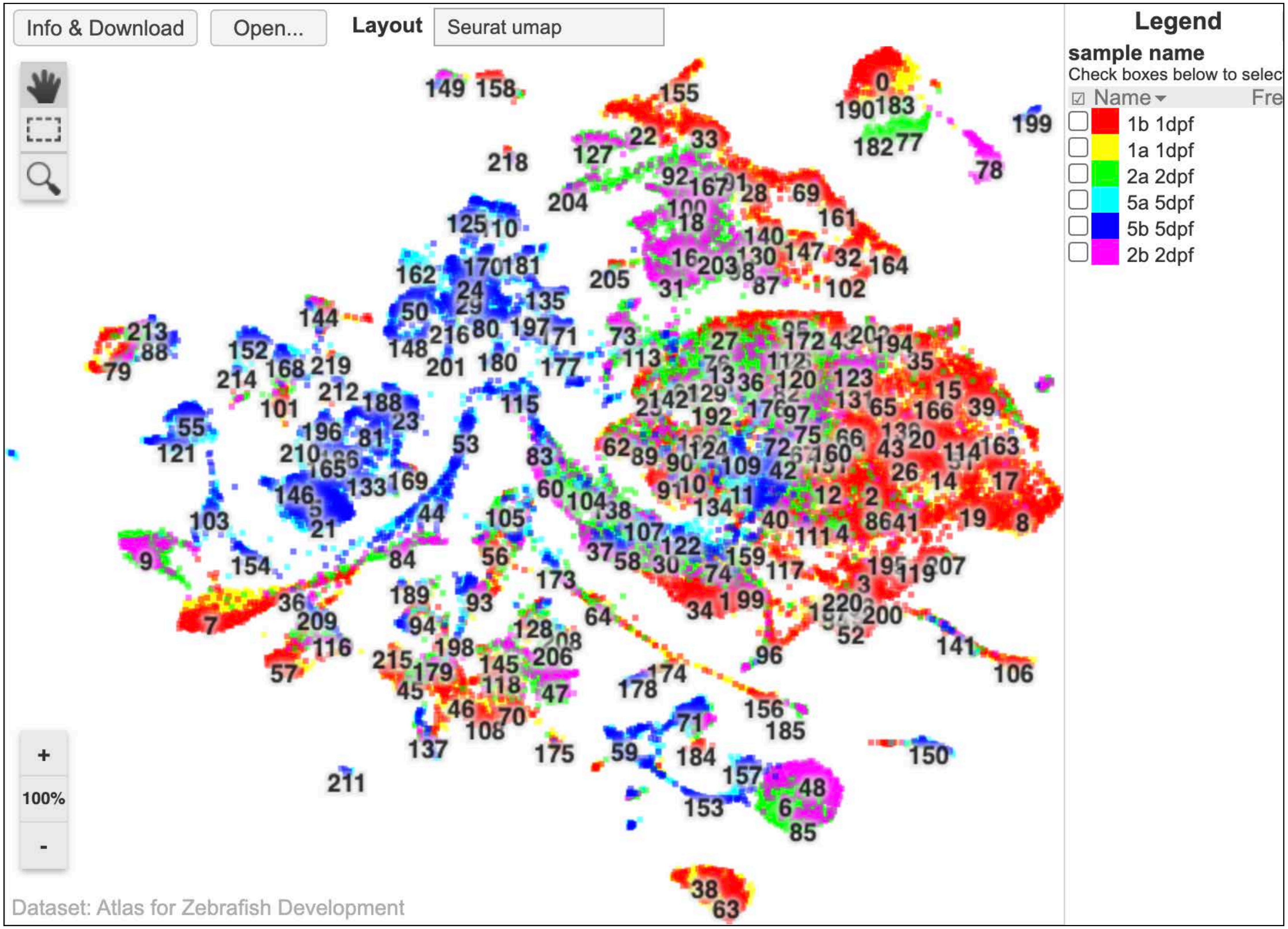

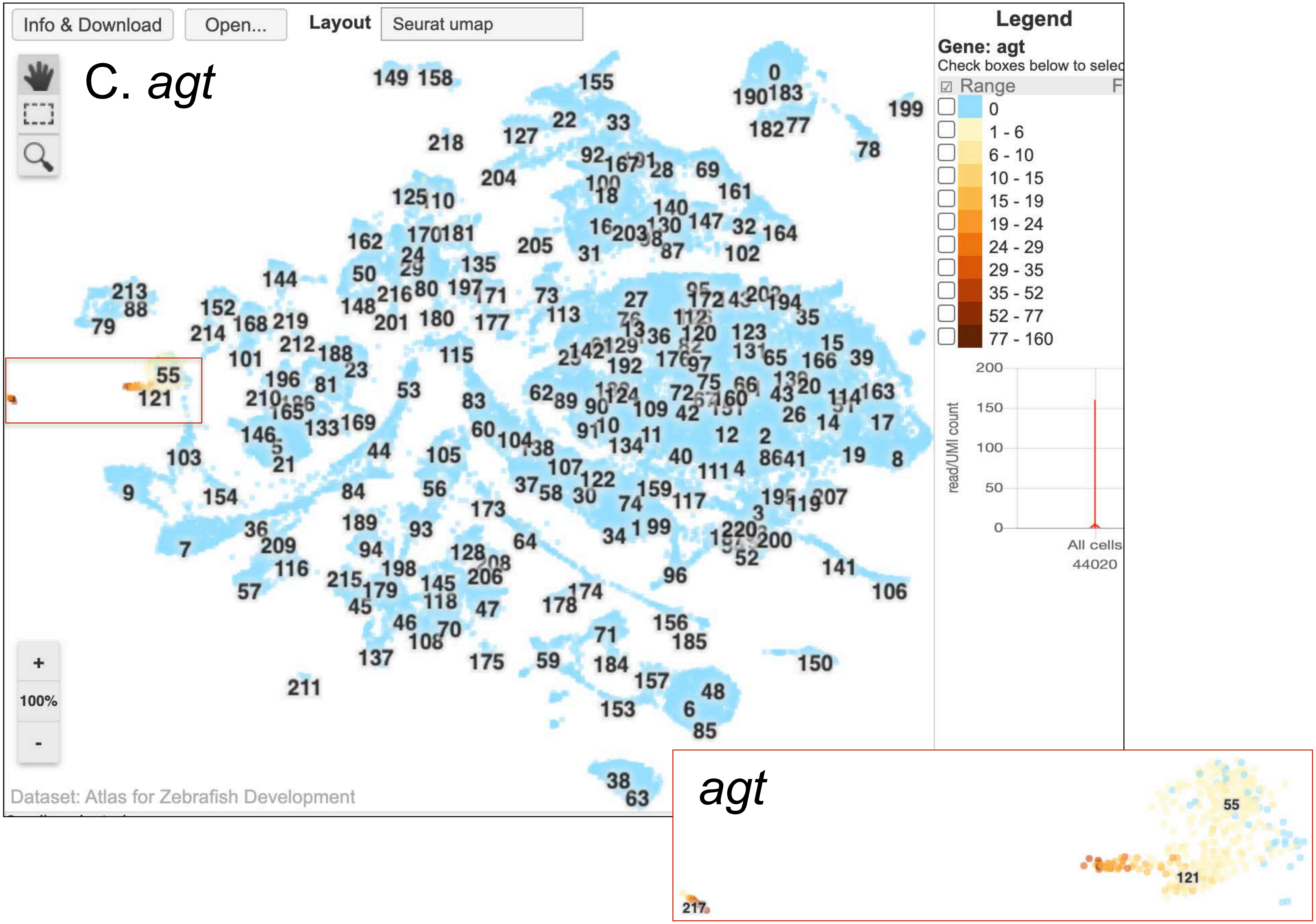


Biology Open • Supplementary information

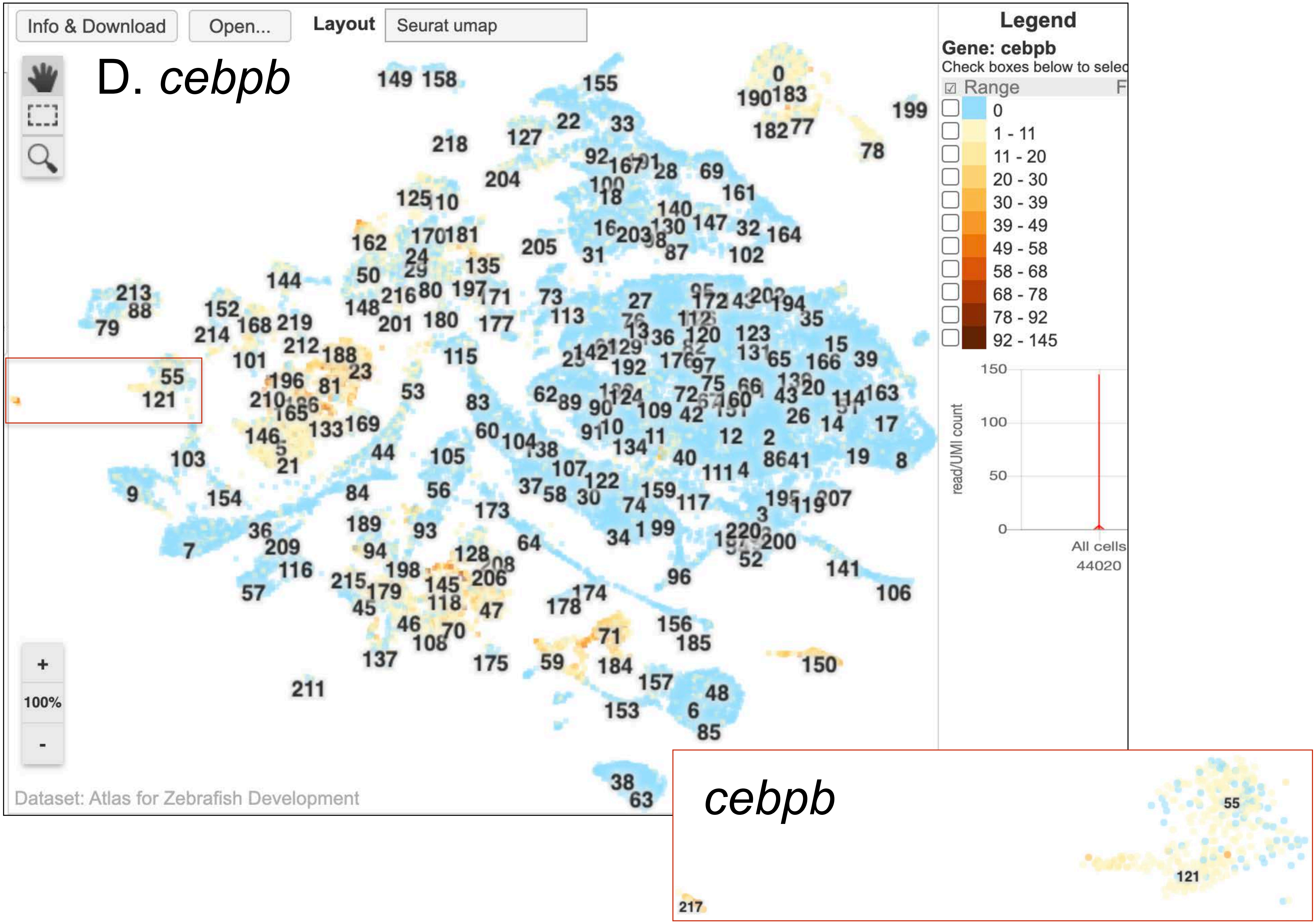

Info & Download
Open...
Layout
Seurat umap
D. cebpb
Legend
Gene: cebpb
Check boxes below to selec
Range
0
1 - 11
11 - 20
20 - 30
30 - 39
39 - 49
49 - 58
58 - 68
68 - 78
78 - 92
92 - 145
read/UMI count
150
100
50
0
All cells
44020
100%
Dataset: Atlas for Zebrafish Development
cebpb
55
121
217

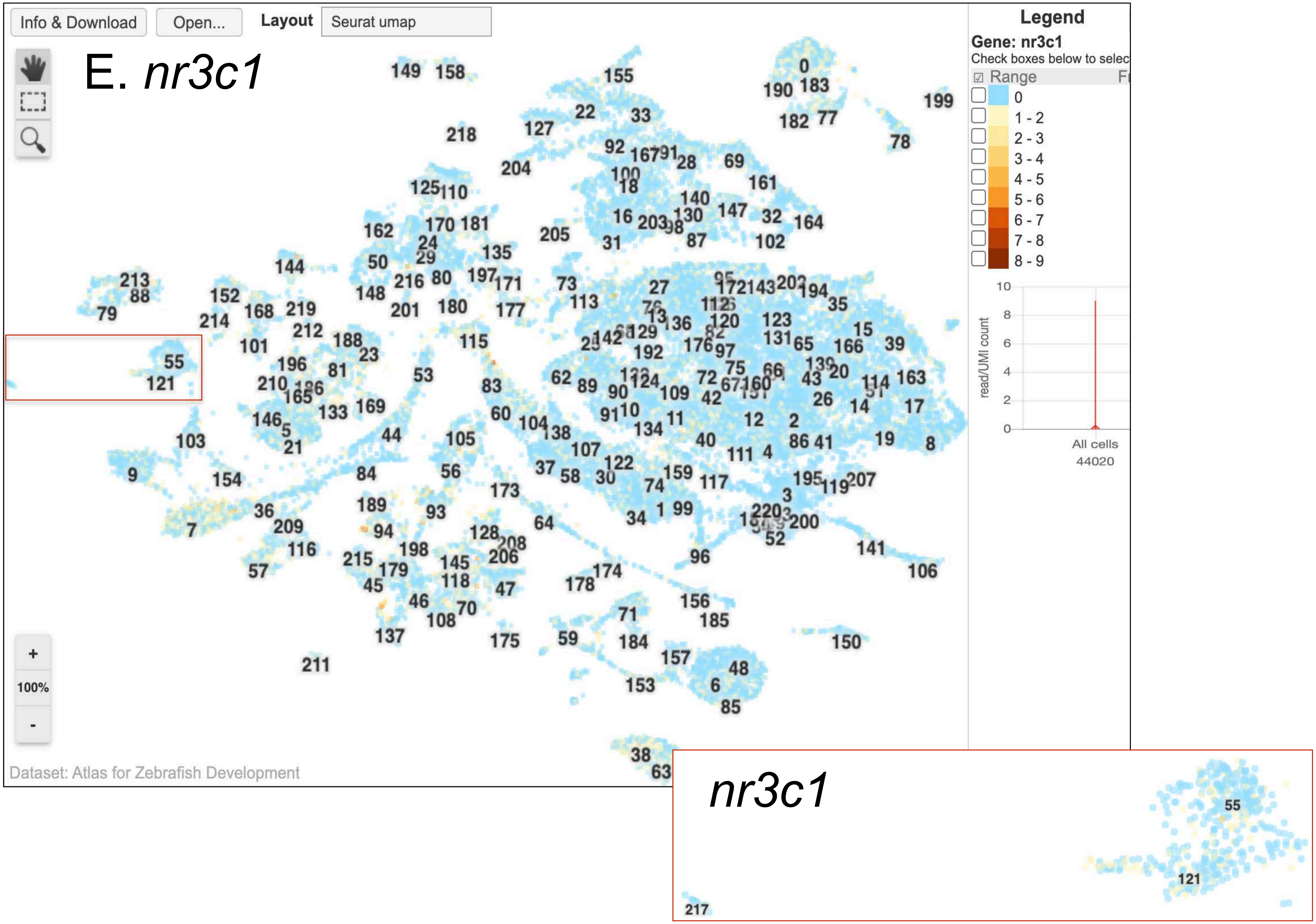


Biology Open • Supplementary information

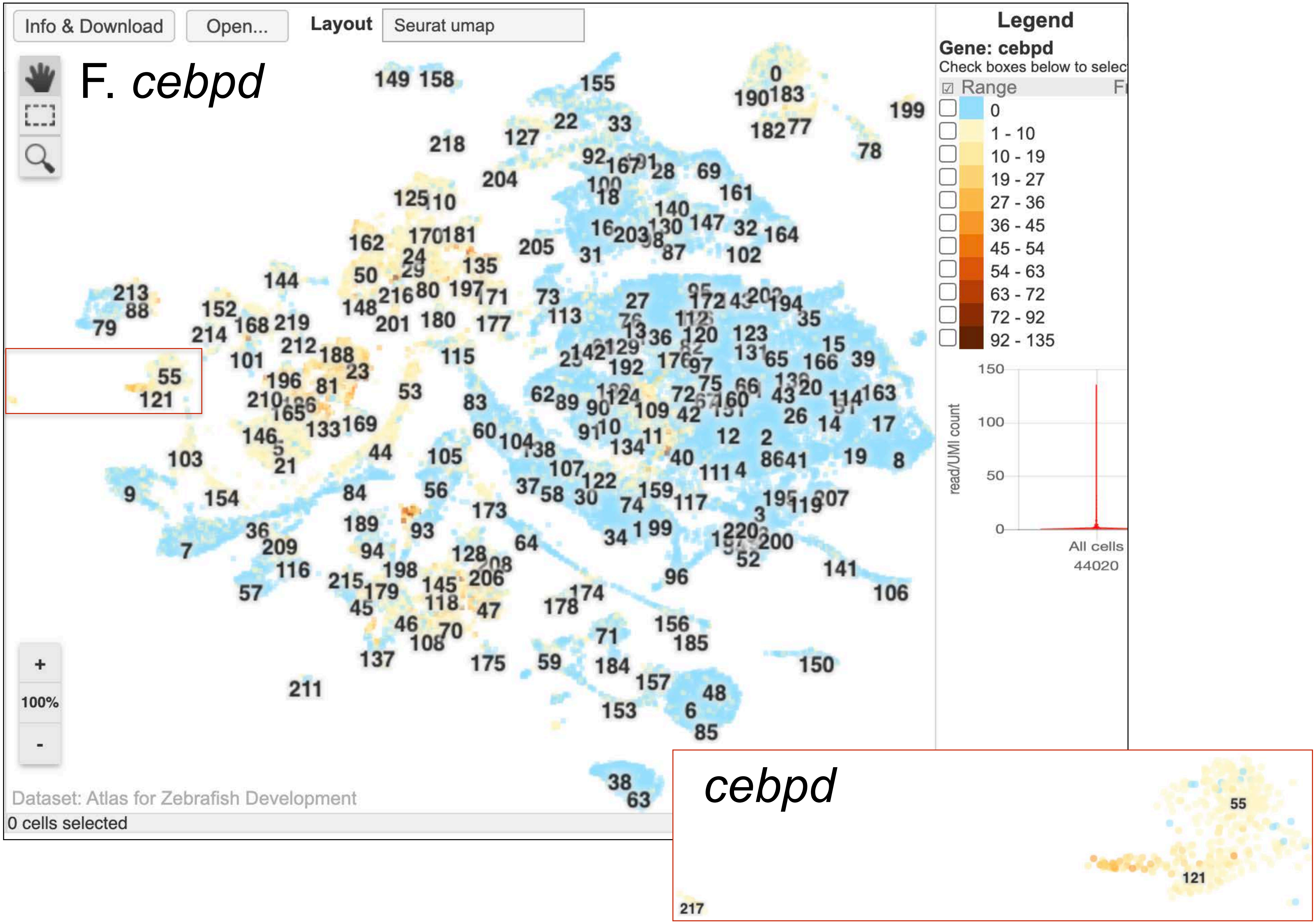


Biology Open • Supplementary information

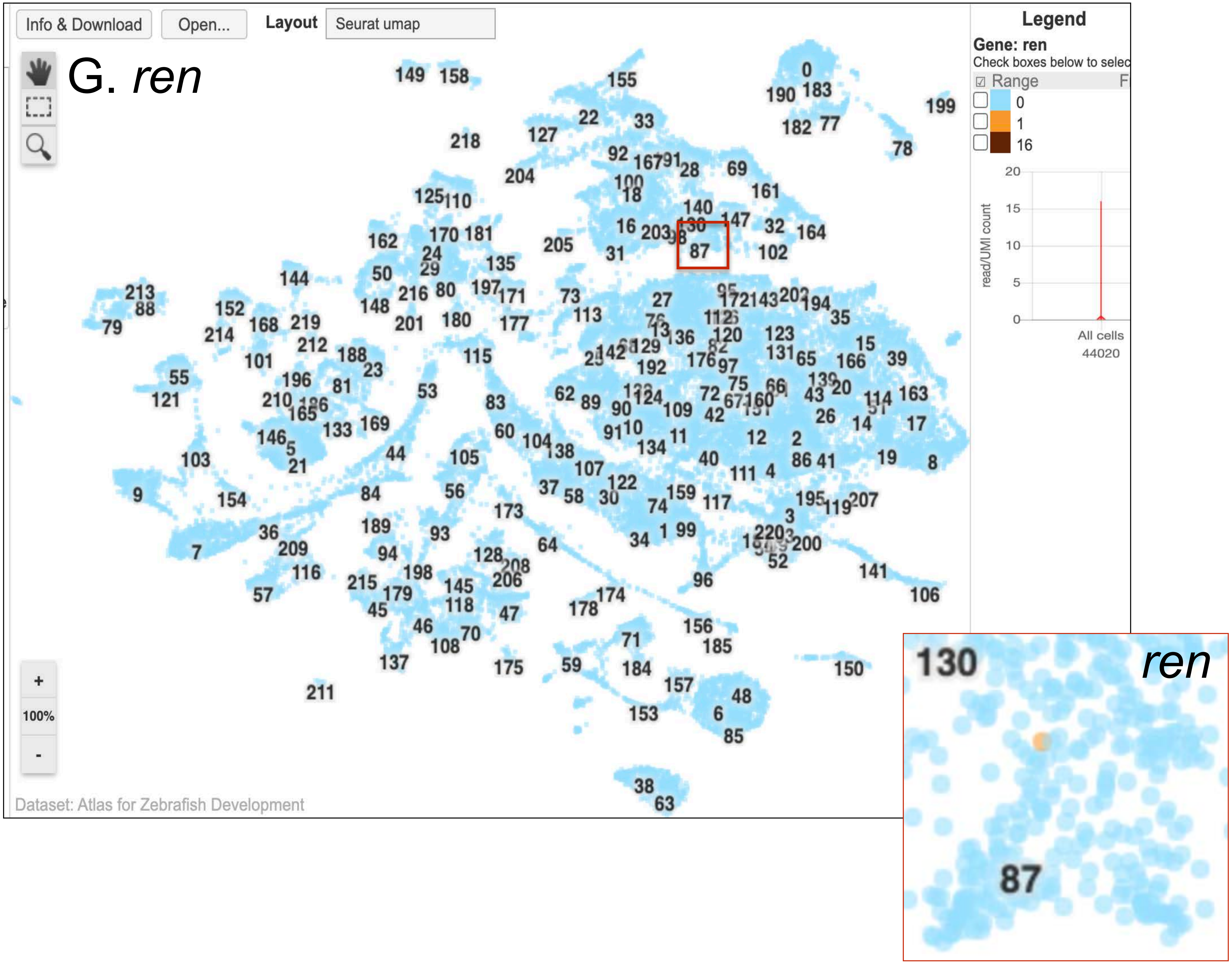


Biology Open • Supplementary information

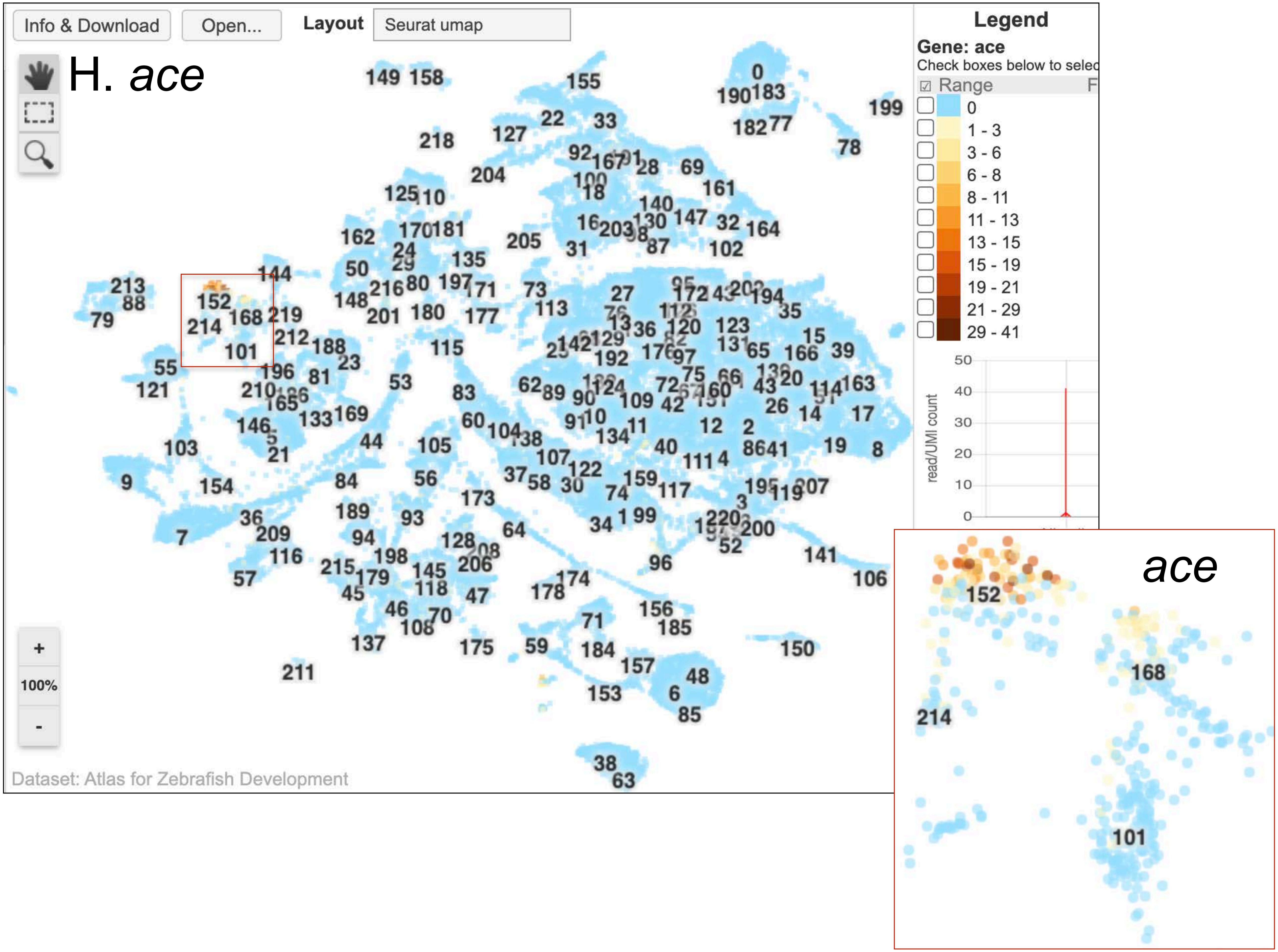


Biology Open • Supplementary information

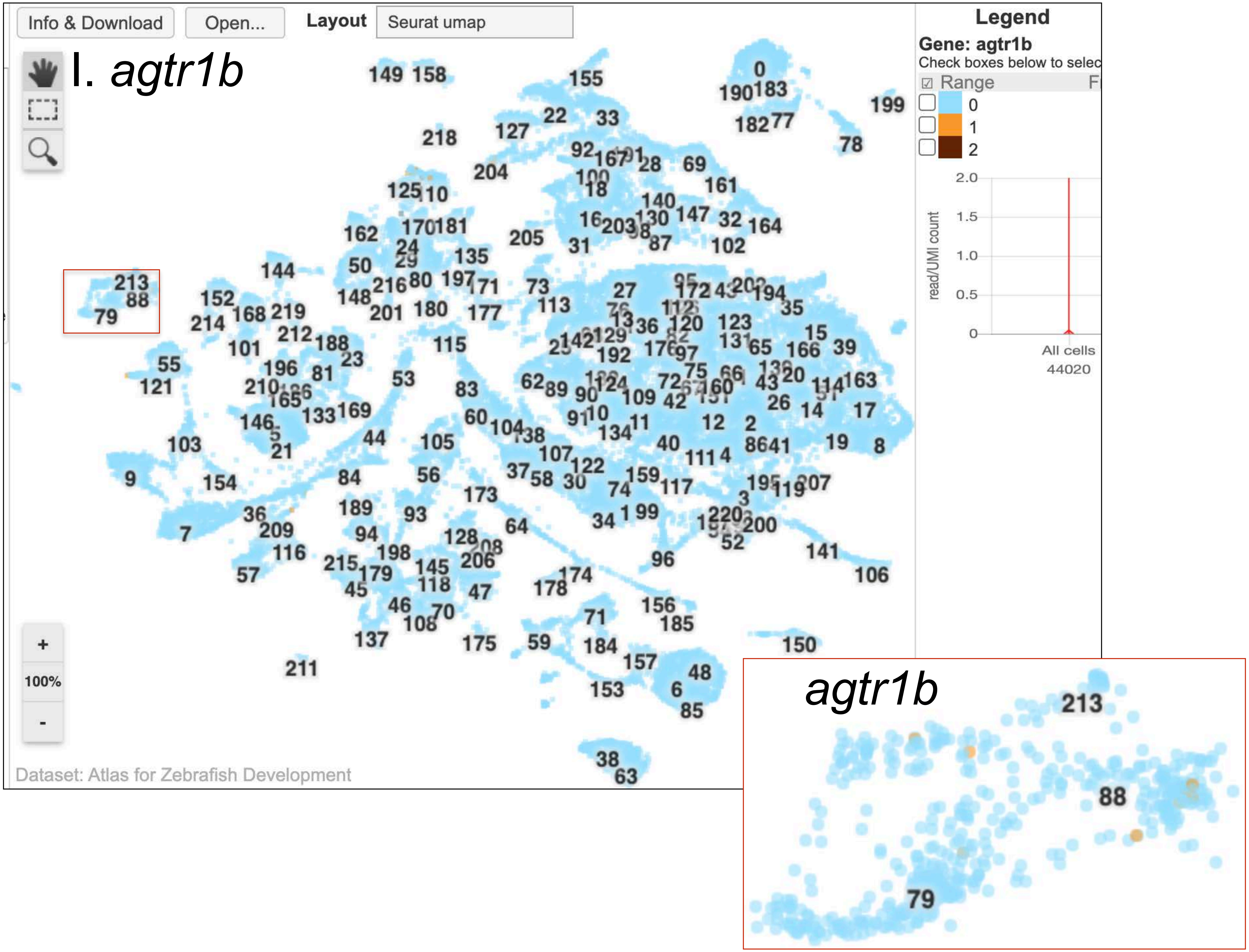
Info & Download
Open...
Layout
Seurat umap
I. agtr1b
Legend
Gene: agtr1b
Check boxes below to selec
Range
0
1
2
read/UMI count
All cells
44020
100%
Dataset: Atlas for Zebrafish Development
agtr1b
213
88
79

Biology Open (2021): doi:10.1242/bio.058172 Supplementary information

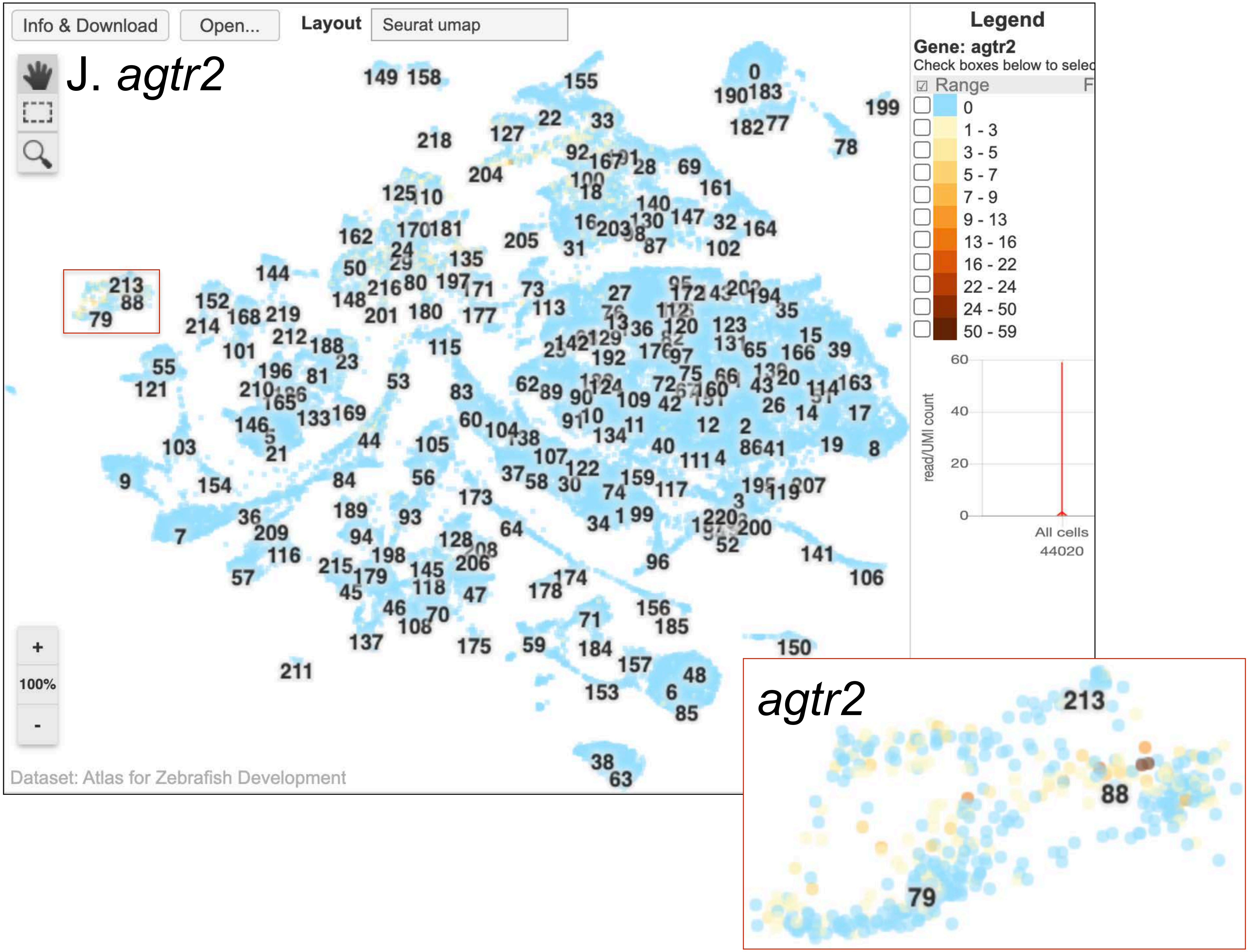


Biology Open • Supplementary information

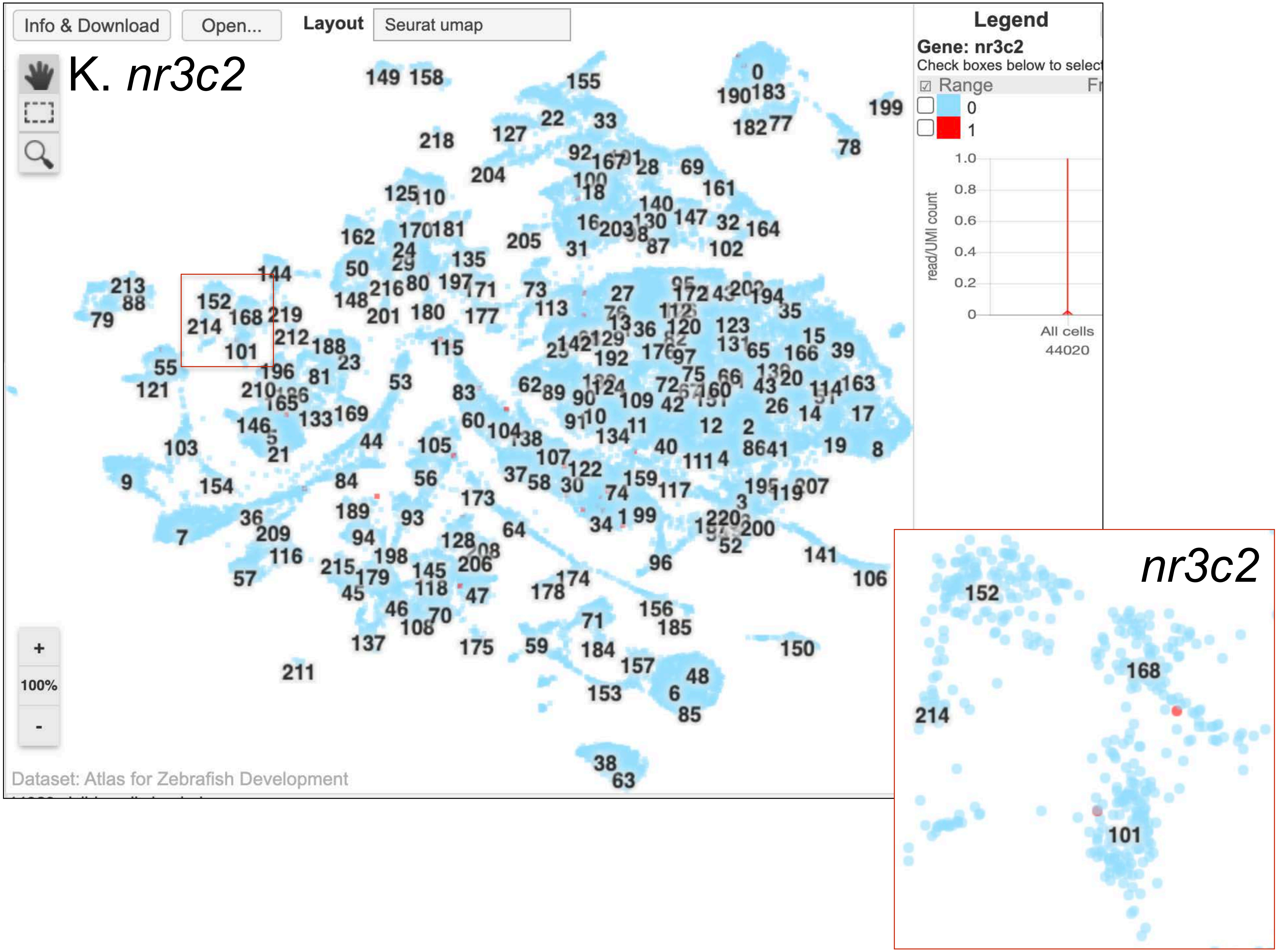


Biology Open • Supplementary information

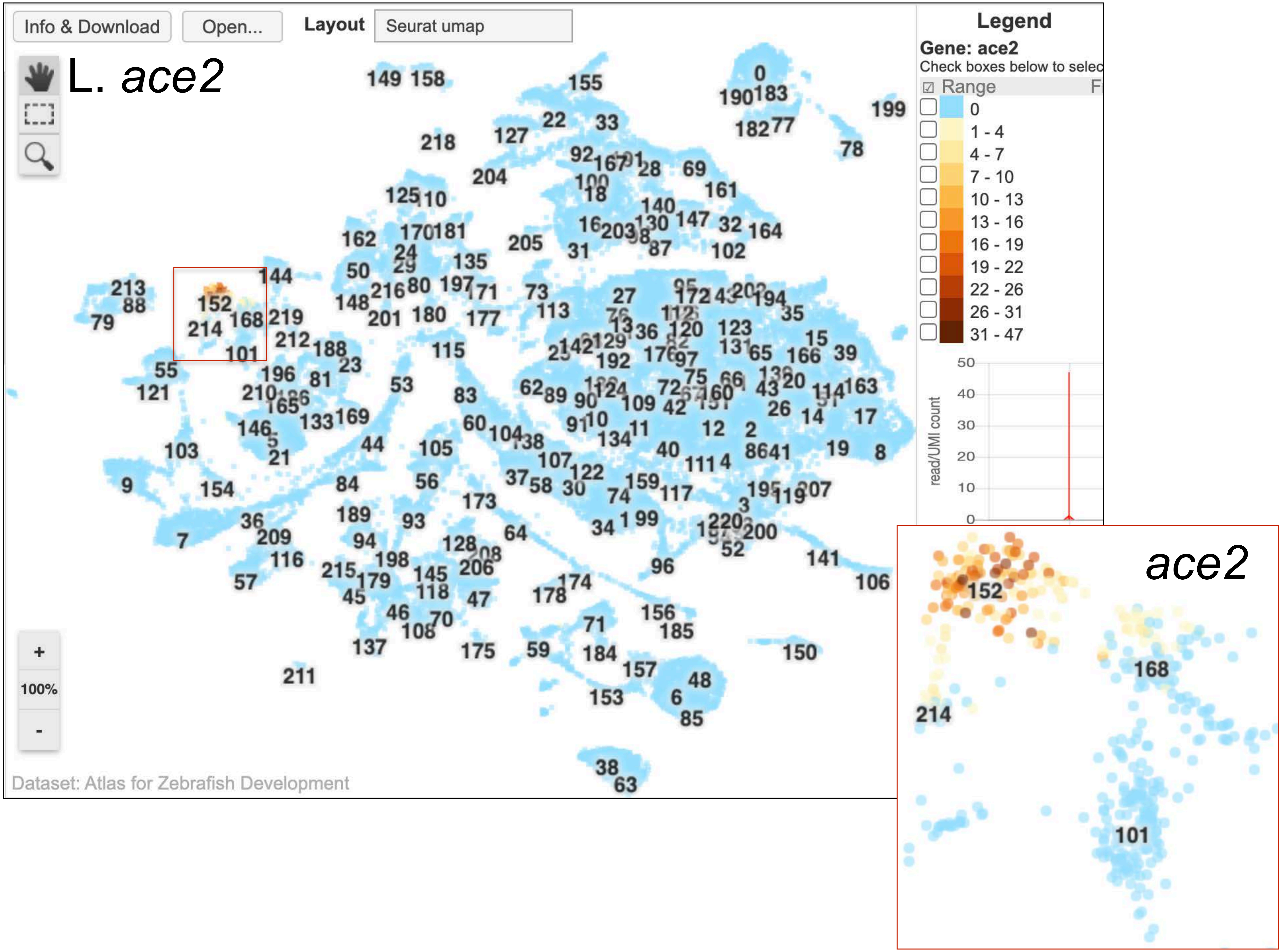
Info & Download
Open...
Layout
Seurat umap
L. ace2
Legend
Gene: ace2
Check boxes below to selec
Range
0
1 - 4
4 - 7
7 - 10
10 - 13
13 - 16
16 - 19
19 - 22
22 - 26
26 - 31
31 - 47
read/UMI count
100%
Dataset: Atlas for Zebrafish Development
ace2
152
168
214
101

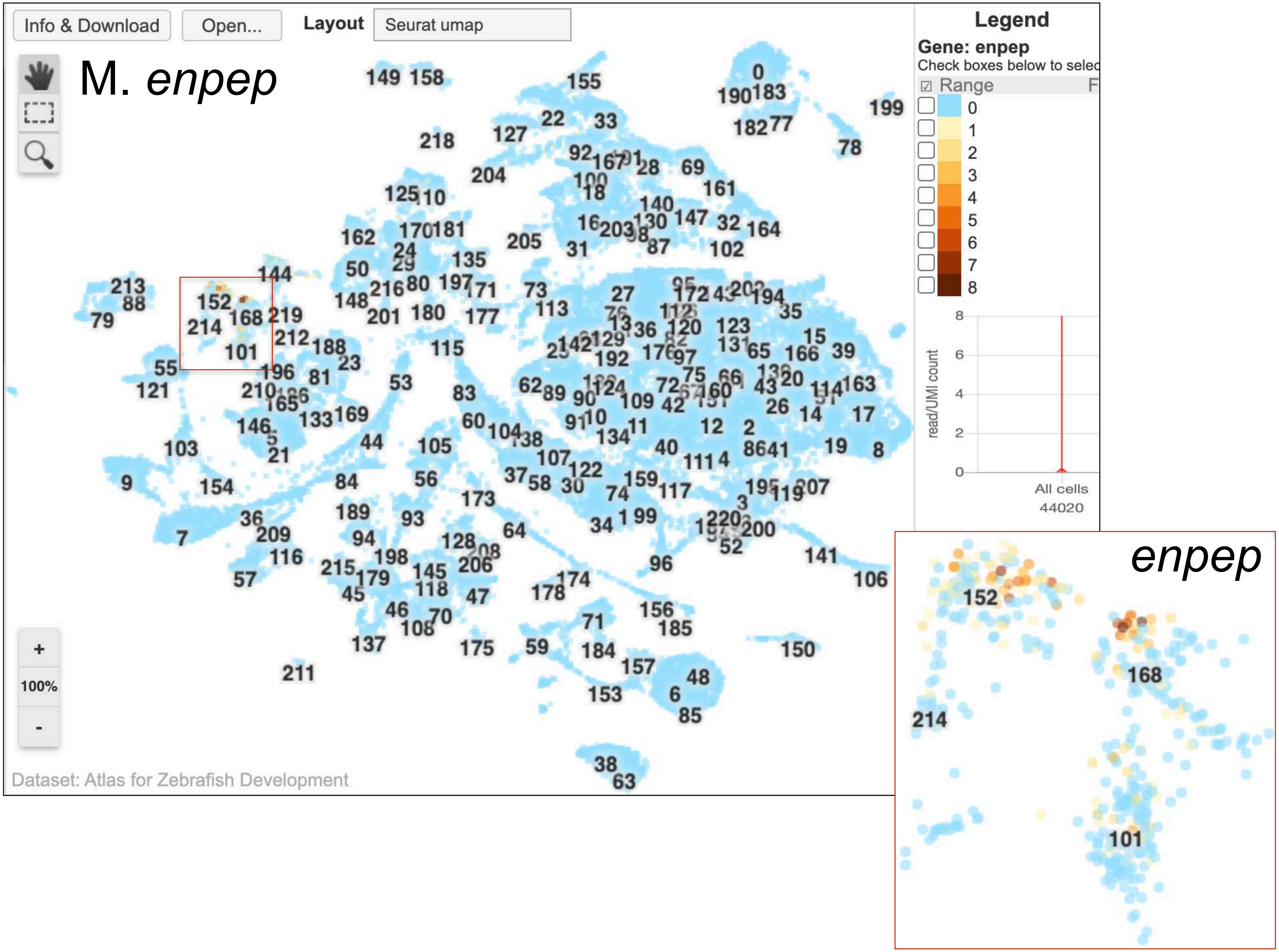
Info & Download
Open...
Layout
Seurat umap
M. enpep
Legend
Gene: enpep
Range
read/UMI count
All cells
44020
Dataset: Atlas for Zebrafish Development
enpep
152
168
214
101

Biology Open (2021): doi:10.1242/bio.058172 Supplementary information

N. *anpepb* (old name: *anpepa*)

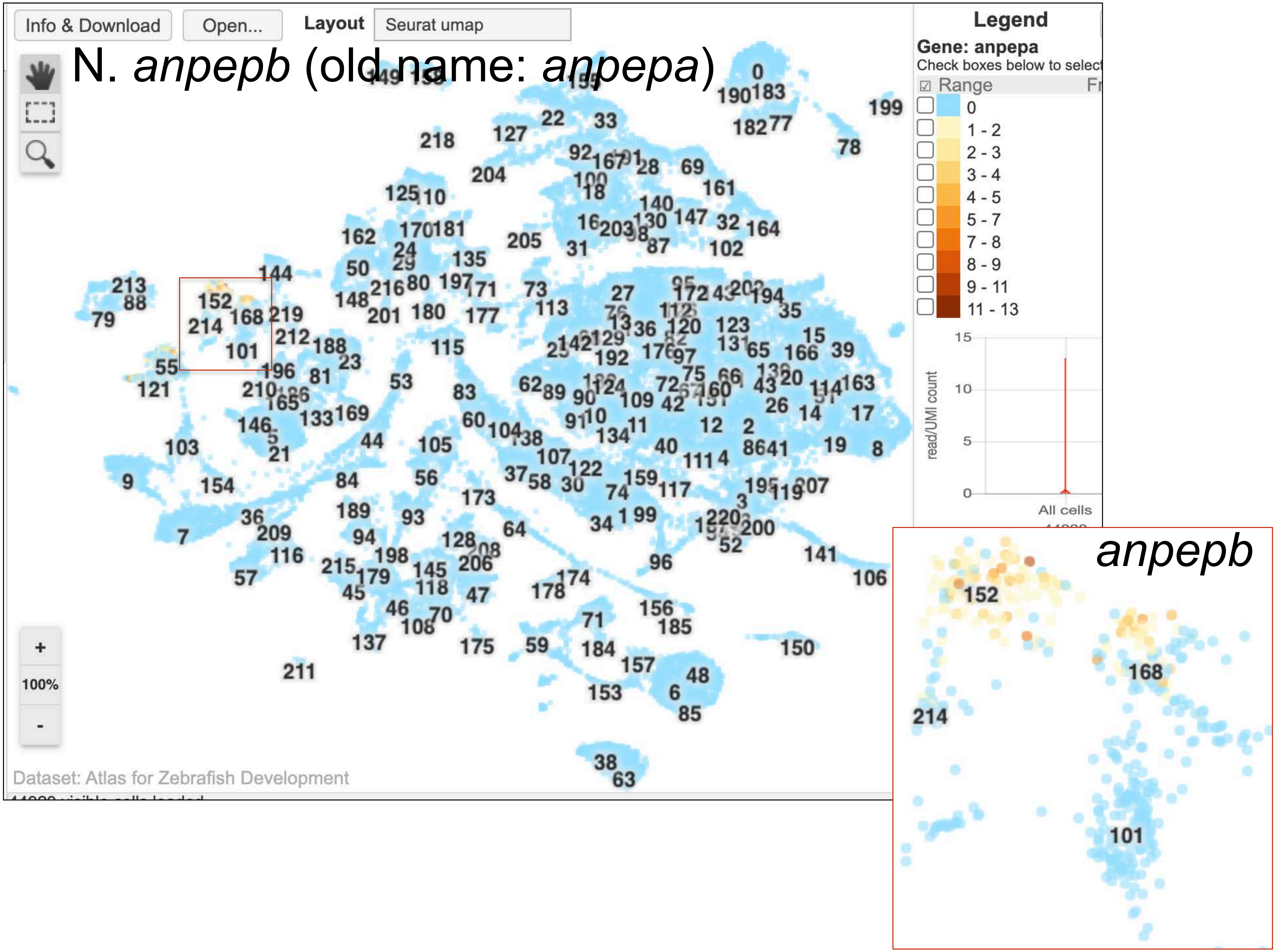


Biology Open • Supplementary information

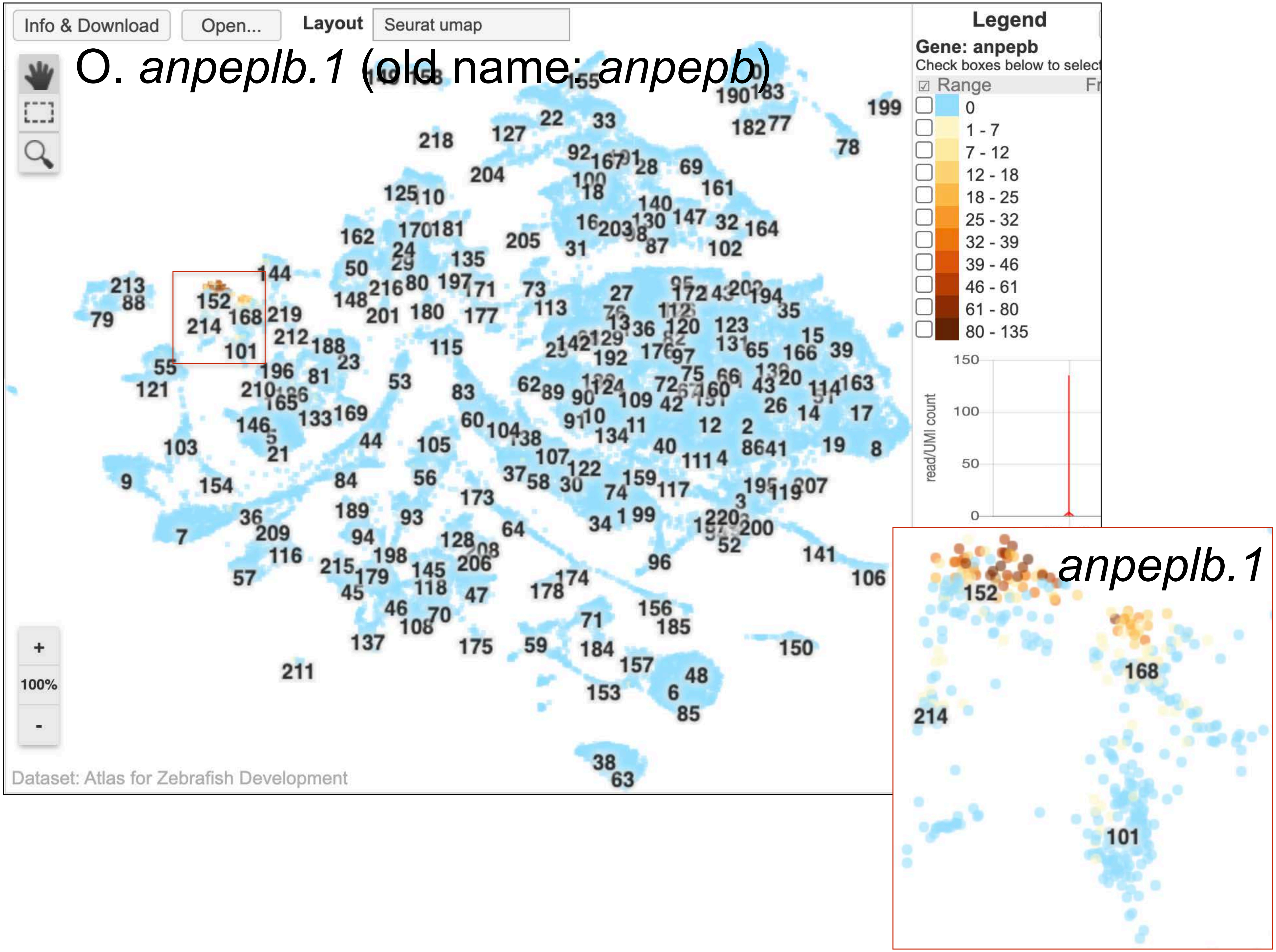
O. anpeplb.1 (old name: anpepb)
Info & Download
Open...
Layout
Seurat umap
Legend
Gene: anpepb
Check boxes below to select
Range
0
1 - 7
7 - 12
12 - 18
18 - 25
25 - 32
32 - 39
39 - 46
46 - 61
61 - 80
80 - 135
read/UMI count
150
100
50
0
100%
Dataset: Atlas for Zebrafish Development
anpeplb.1
152
168
214
101

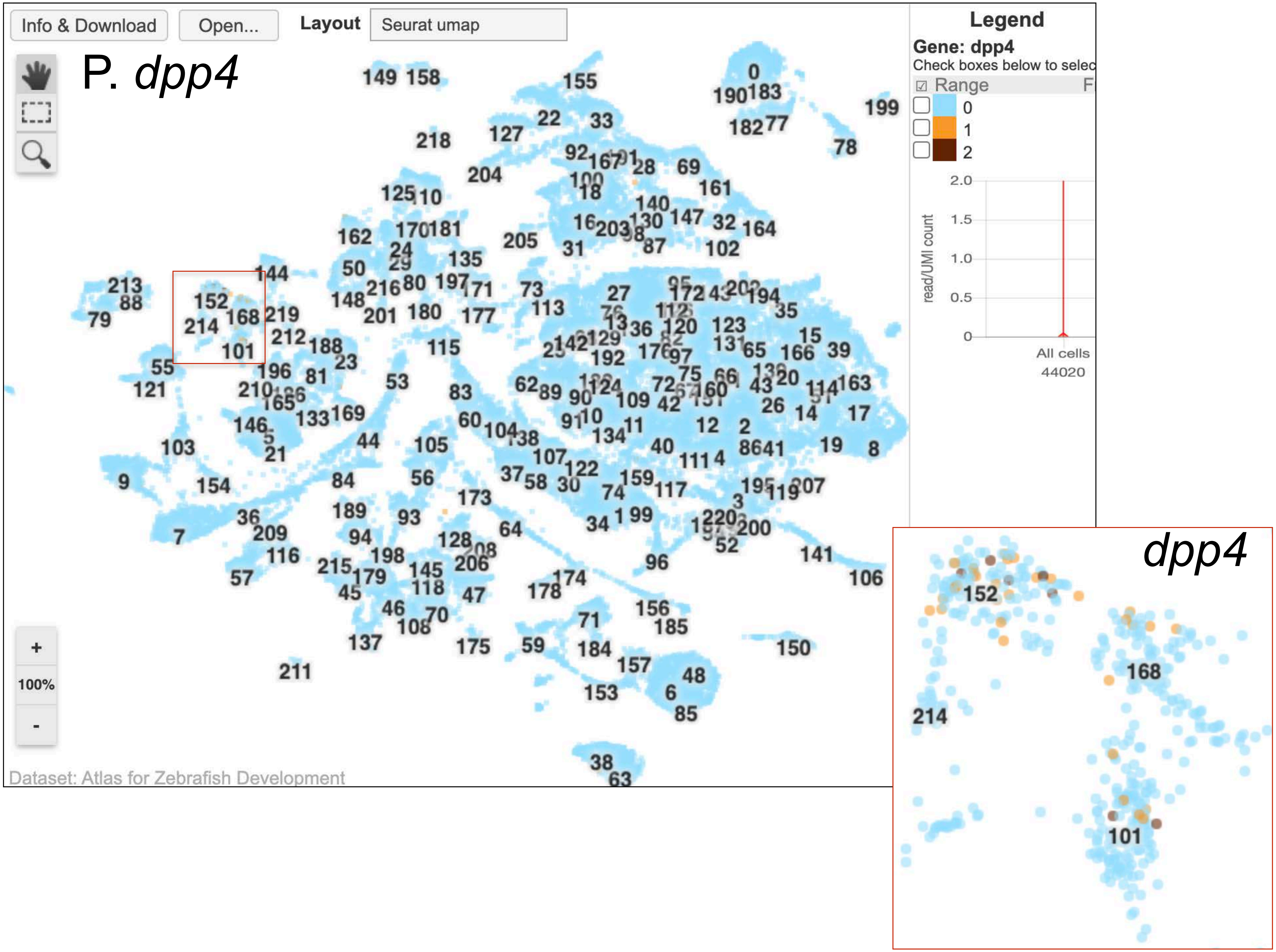
P. *dpp4*
Info & Download
Open...
Layout
Seurat umap
Legend
Gene: dpp4
Check boxes below to selec
Range
0
1
2
read/UMI count
All cells
44020
Dataset: Atlas for Zebrafish Development
*dpp4*
152
168
214
101

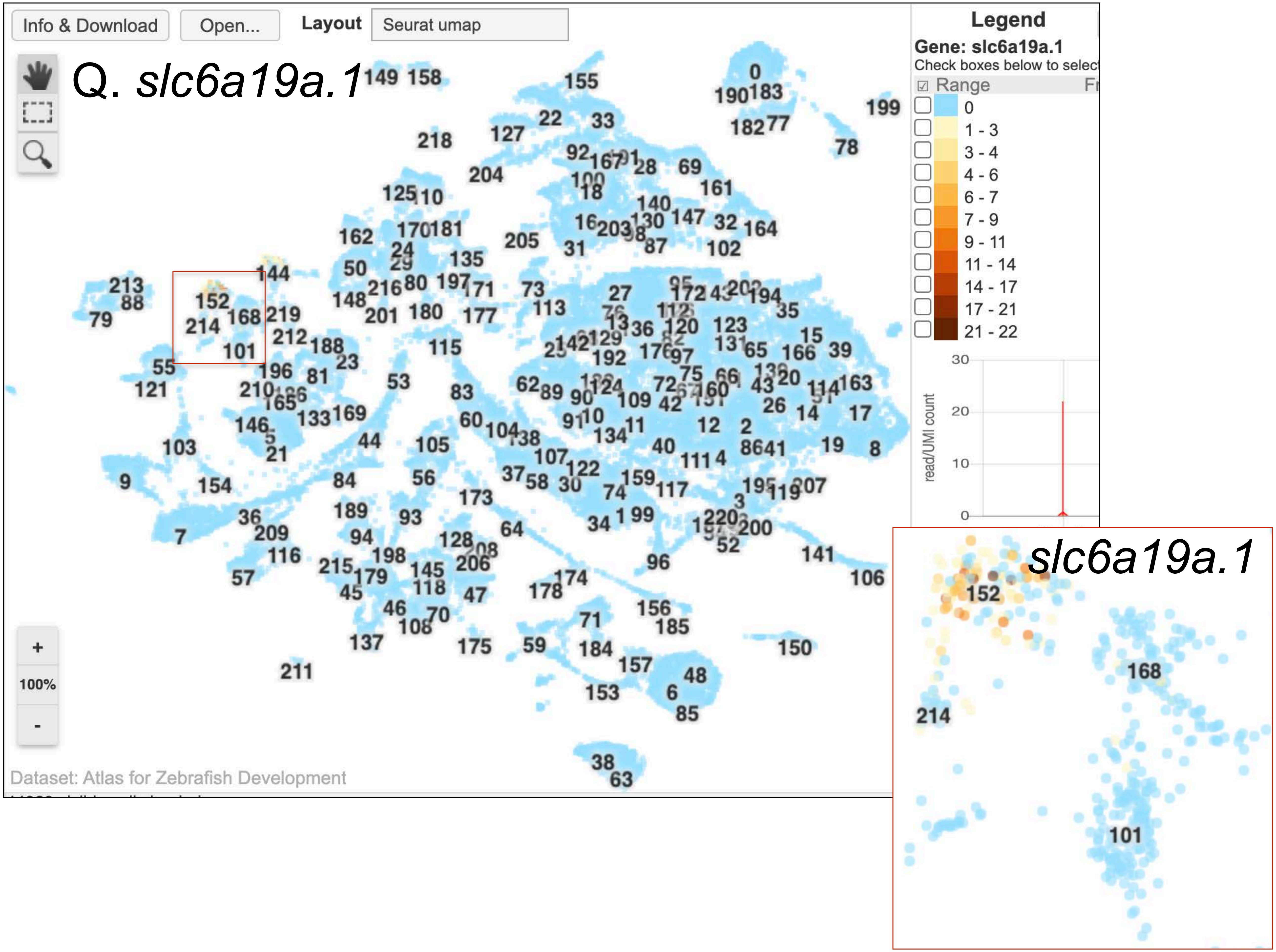
Info & Download
Open...
Layout
Seurat umap
Q. slc6a19a.1
Legend
Gene: slc6a19a.1
Check boxes below to select
Range
0
1 - 3
3 - 4
4 - 6
6 - 7
7 - 9
9 - 11
11 - 14
14 - 17
17 - 21
21 - 22
read/UMI count
30
20
10
0
100%
Dataset: Atlas for Zebrafish Development
slc6a19a.1
152
214
168
101

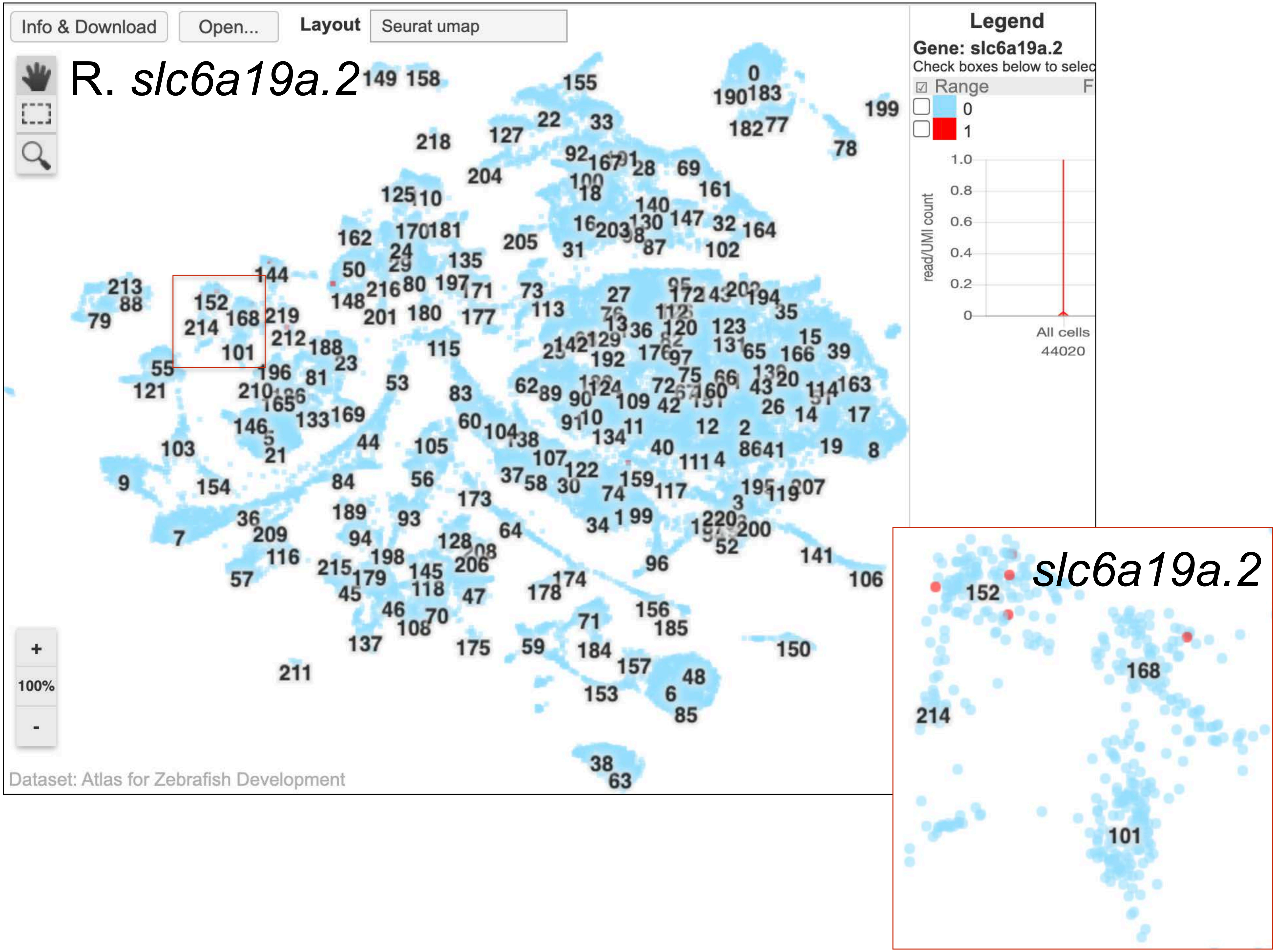
Info & Download
Open...
Layout
Seurat umap
R. slc6a19a.2
Legend
Gene: slc6a19a.2
Check boxes below to selec
Range
0
1
read/UMI count
1.0
0.8
0.6
0.4
0.2
0
All cells
44020
100%
Dataset: Atlas for Zebrafish Development
slc6a19a.2
152
168
214
101

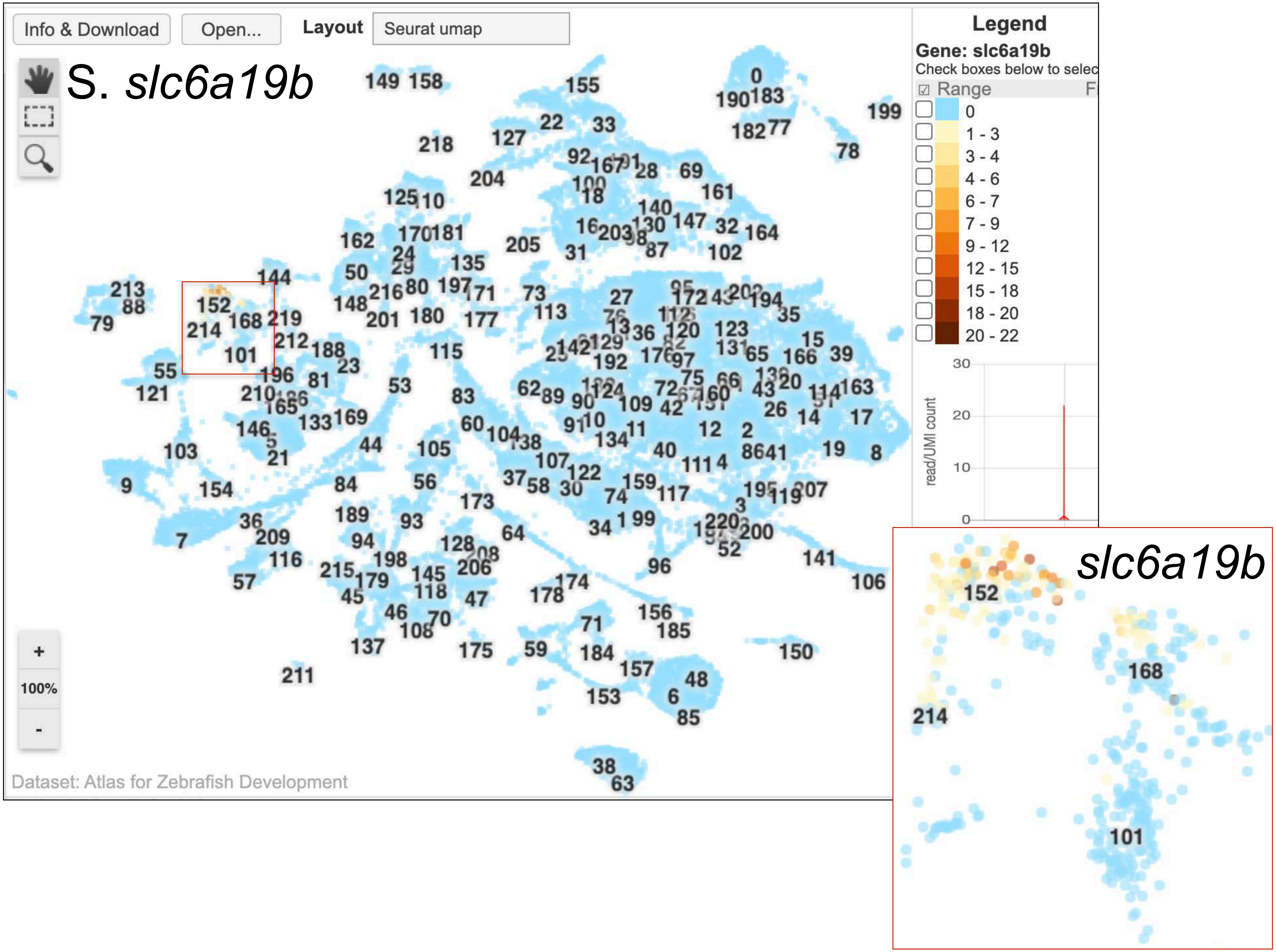


Biology Open • Supplementary information

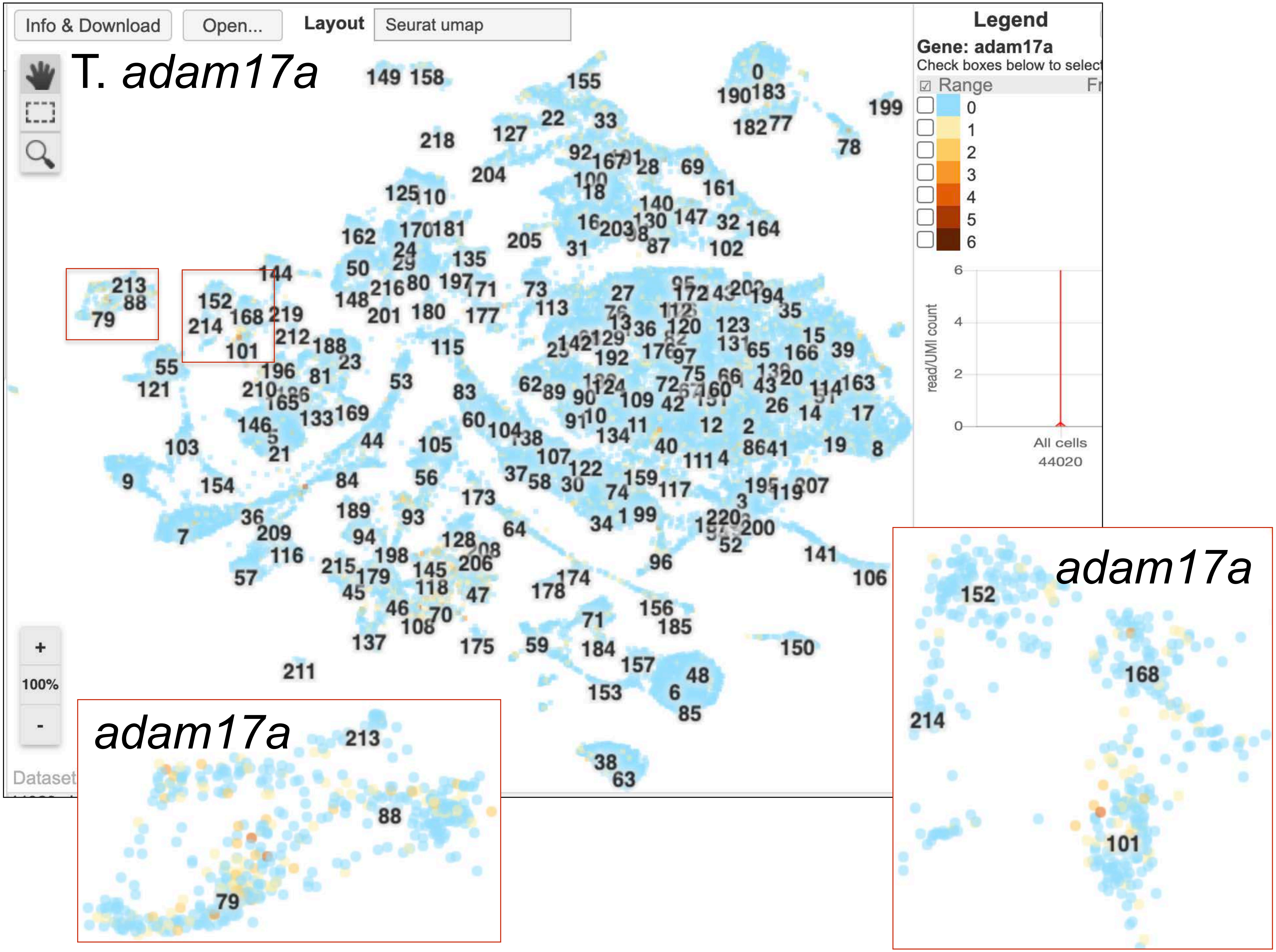


Biology Open • Supplementary information

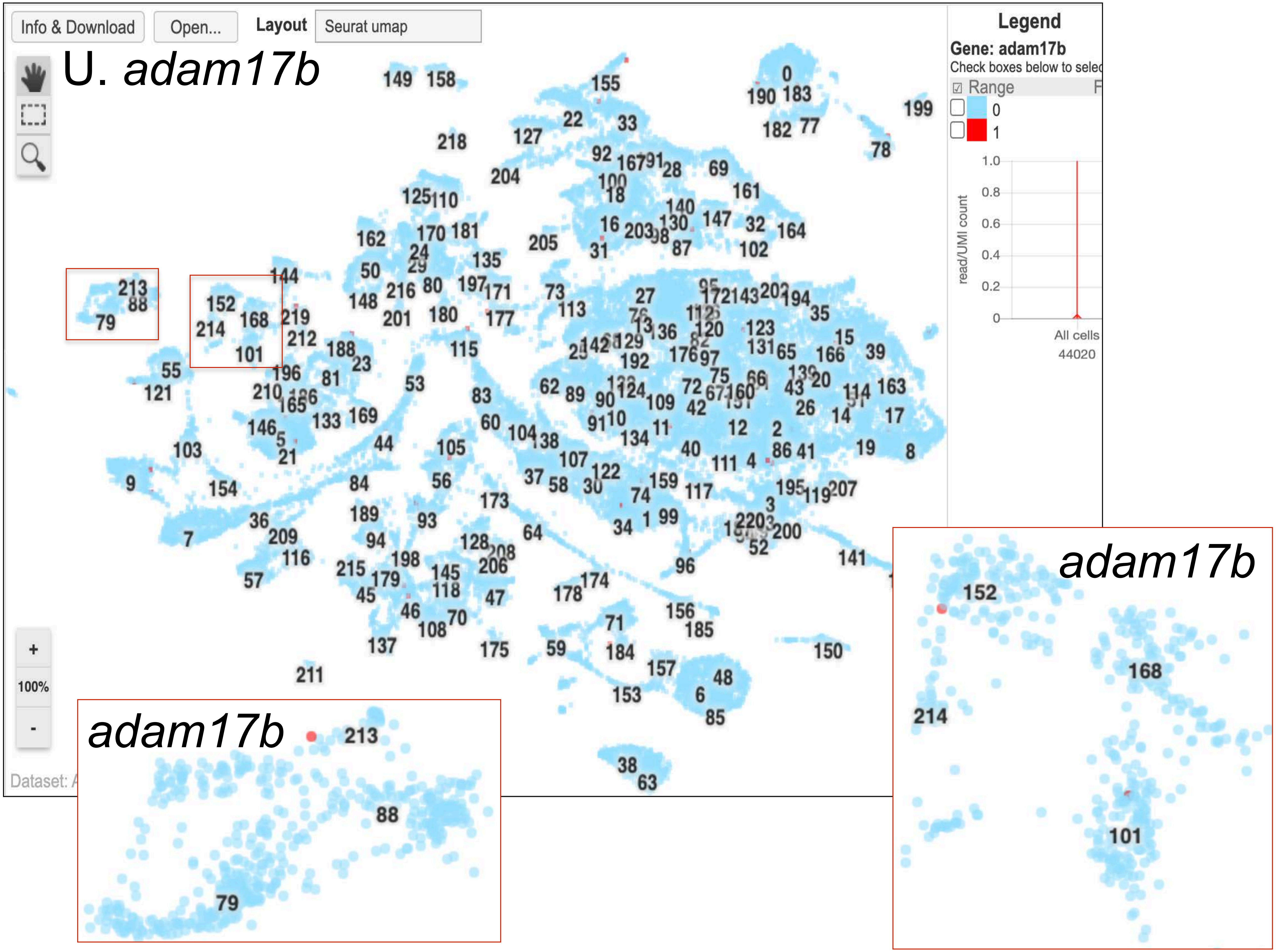


Biology Open • Supplementary information

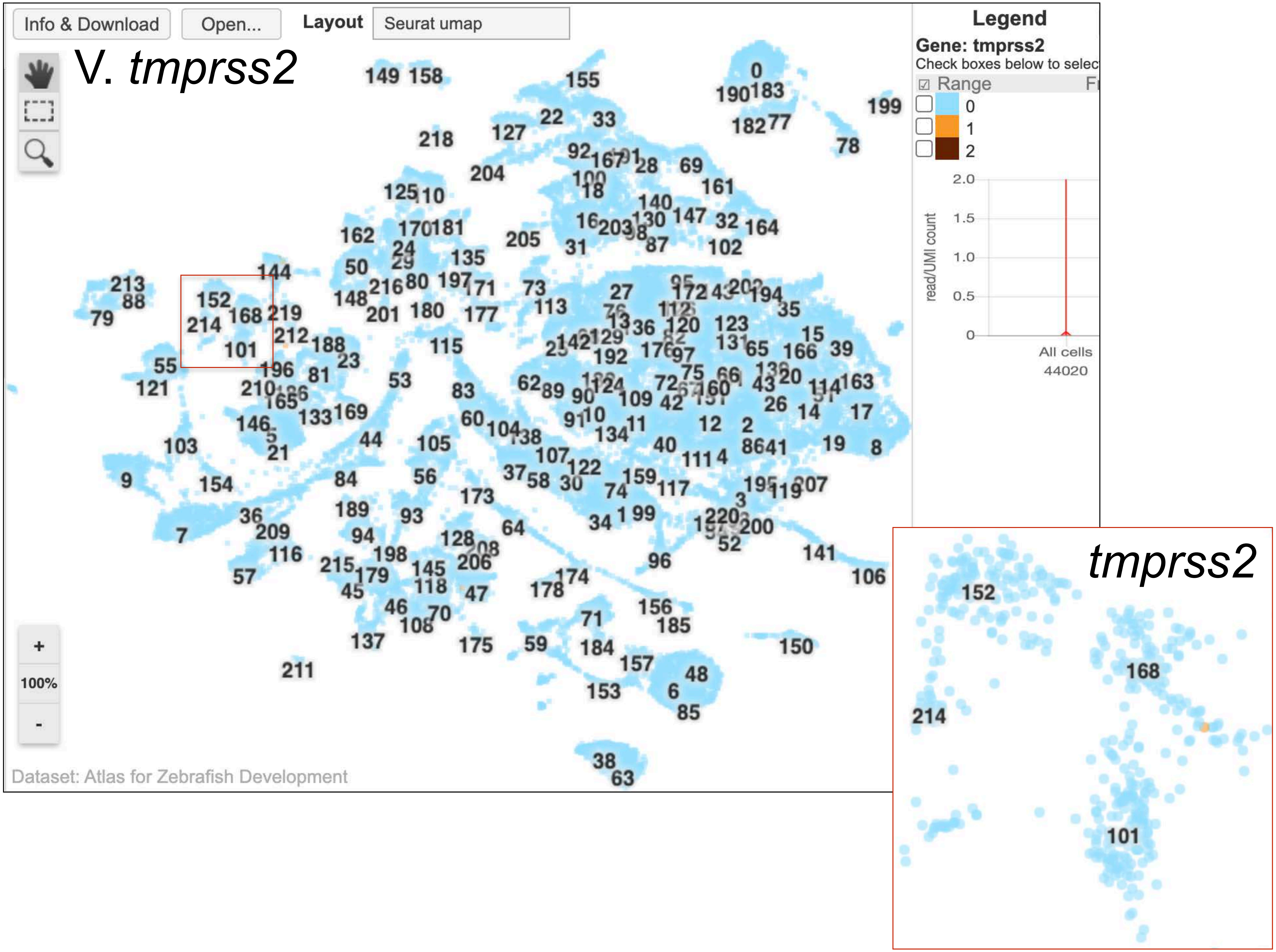


Biology Open • Supplementary information

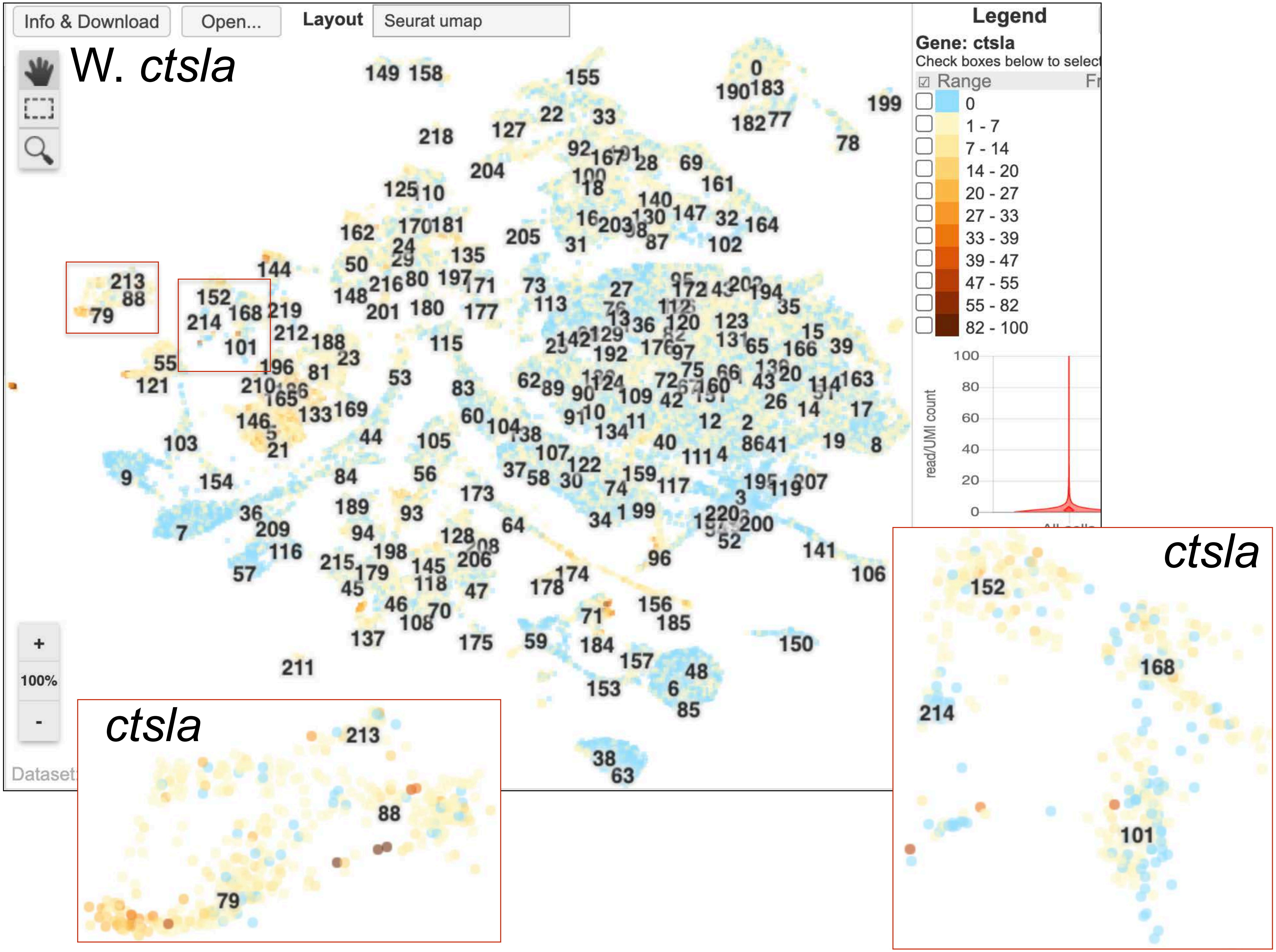
Info & Download
Open...
Layout
Seurat umap
W. ctsla
Legend
Gene: ctsla
Check boxes below to select
Range
0
1 - 7
7 - 14
14 - 20
20 - 27
27 - 33
33 - 39
39 - 47
47 - 55
55 - 82
82 - 100
read/UMI count
100
80
60
40
20
0
+
100%
-
ctsla
213
88
79
ctsla
152
168
214
101

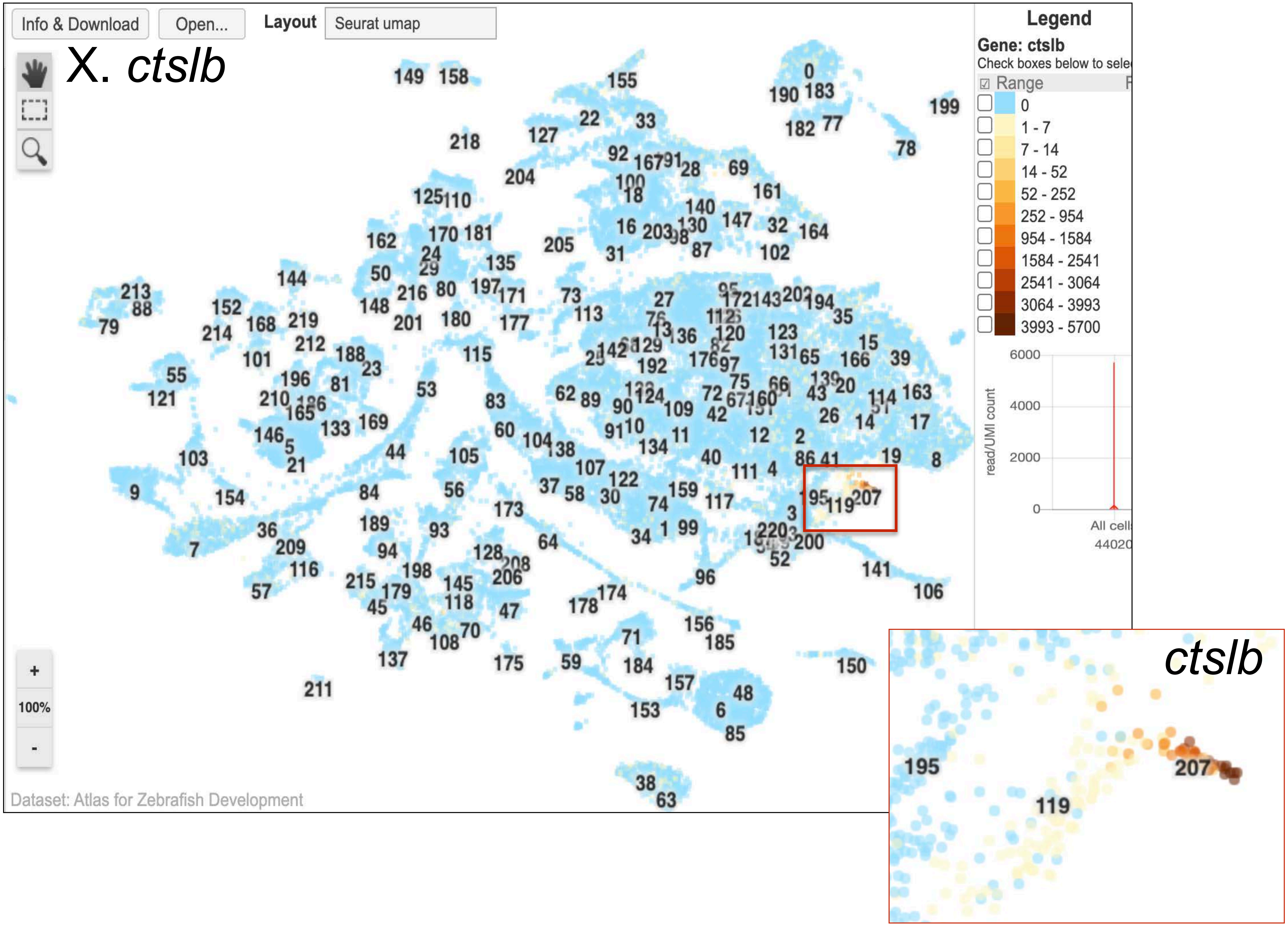
Info & Download
Open...
Layout
Seurat umap
X. *ctslb*
Legend
Gene: ctslb
Check boxes below to sele
Range
0
1 - 7
7 - 14
14 - 52
52 - 252
252 - 954
954 - 1584
1584 - 2541
2541 - 3064
3064 - 3993
3993 - 5700
6000
4000
2000
0
read/UMI count
All cell
44020
100%
Dataset: Atlas for Zebrafish Development
*ctslb*
195
119
207

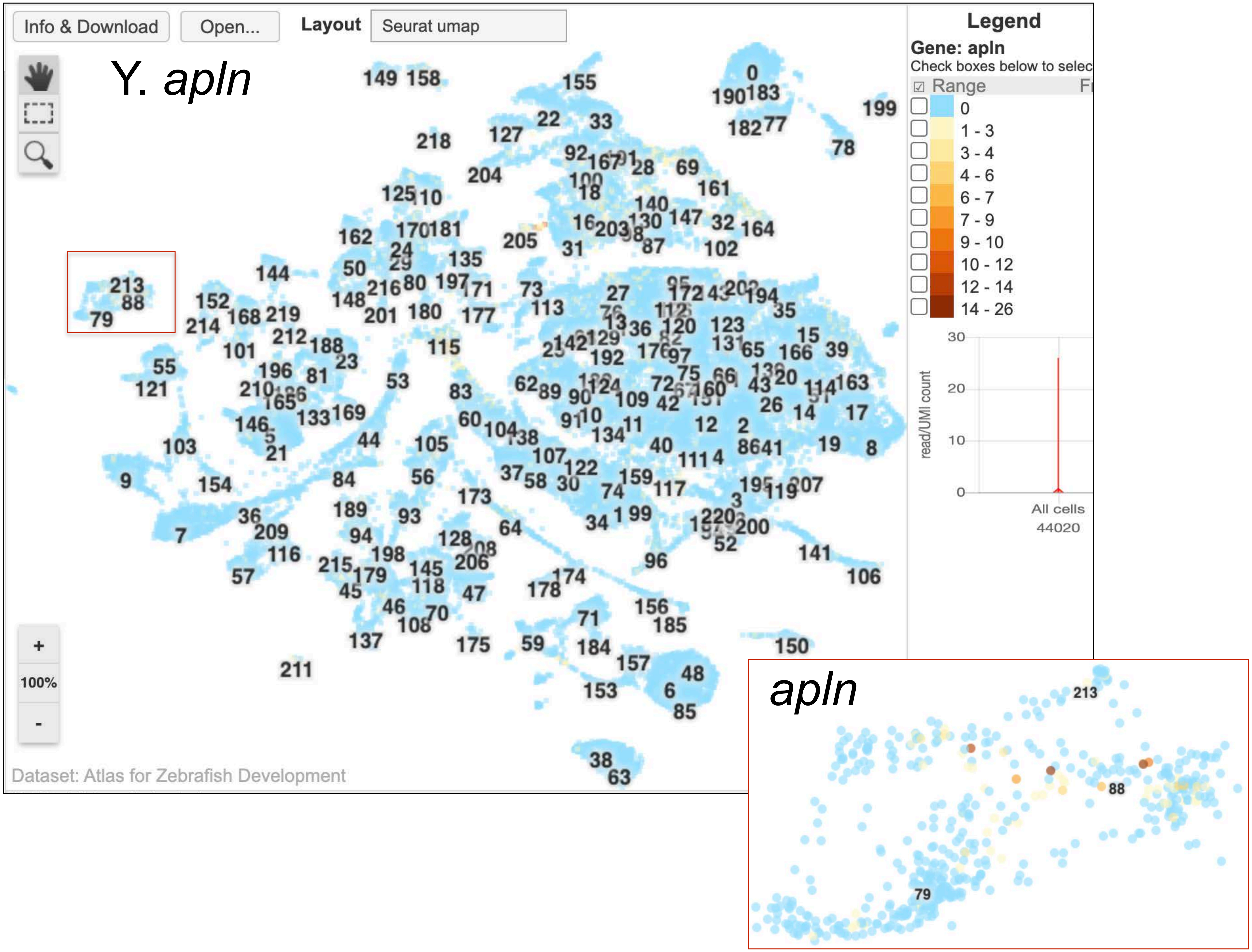


Biology Open • Supplementary information

Biology Open (2021): doi:10.1242/bio.058172 Supplementary information

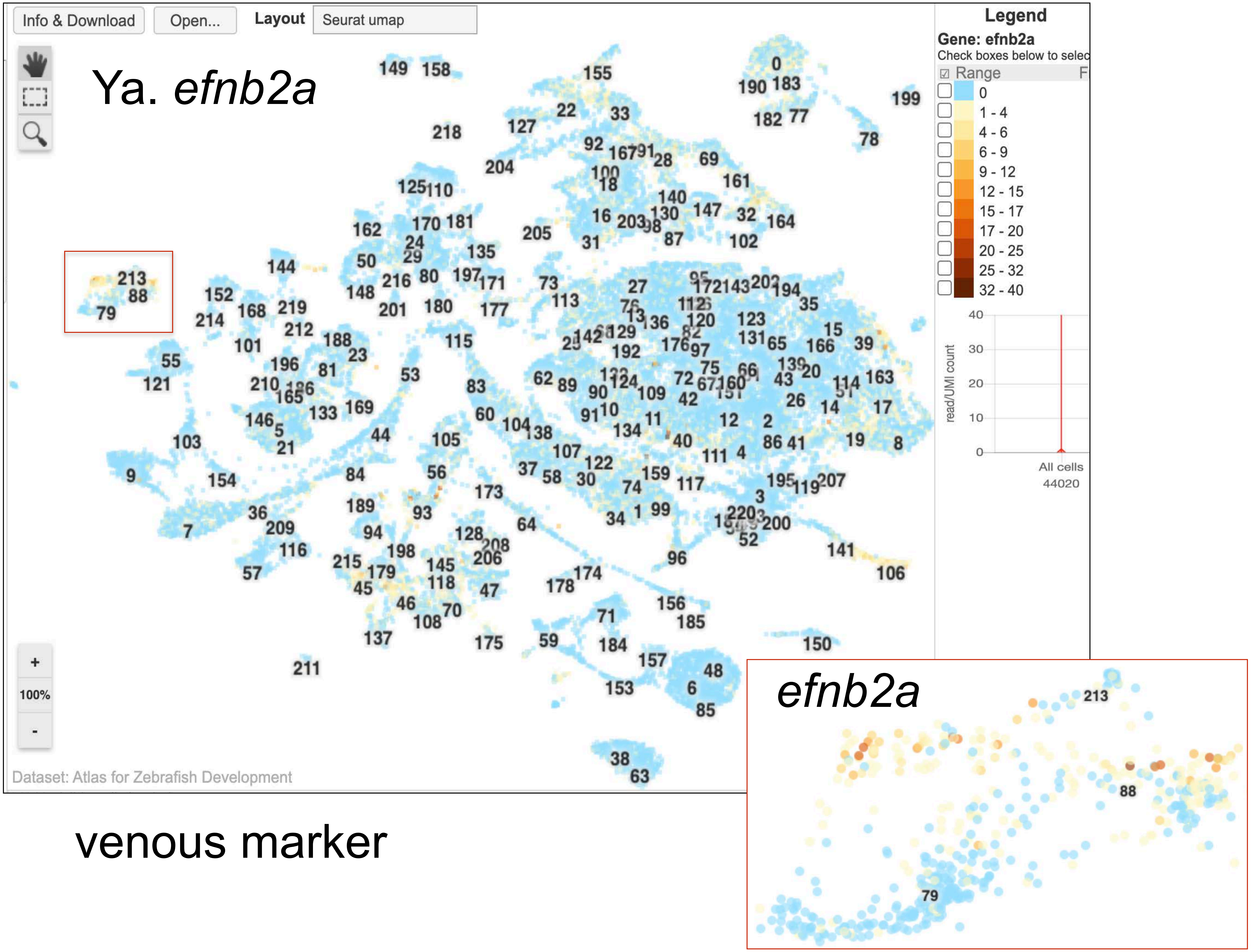


venous marker

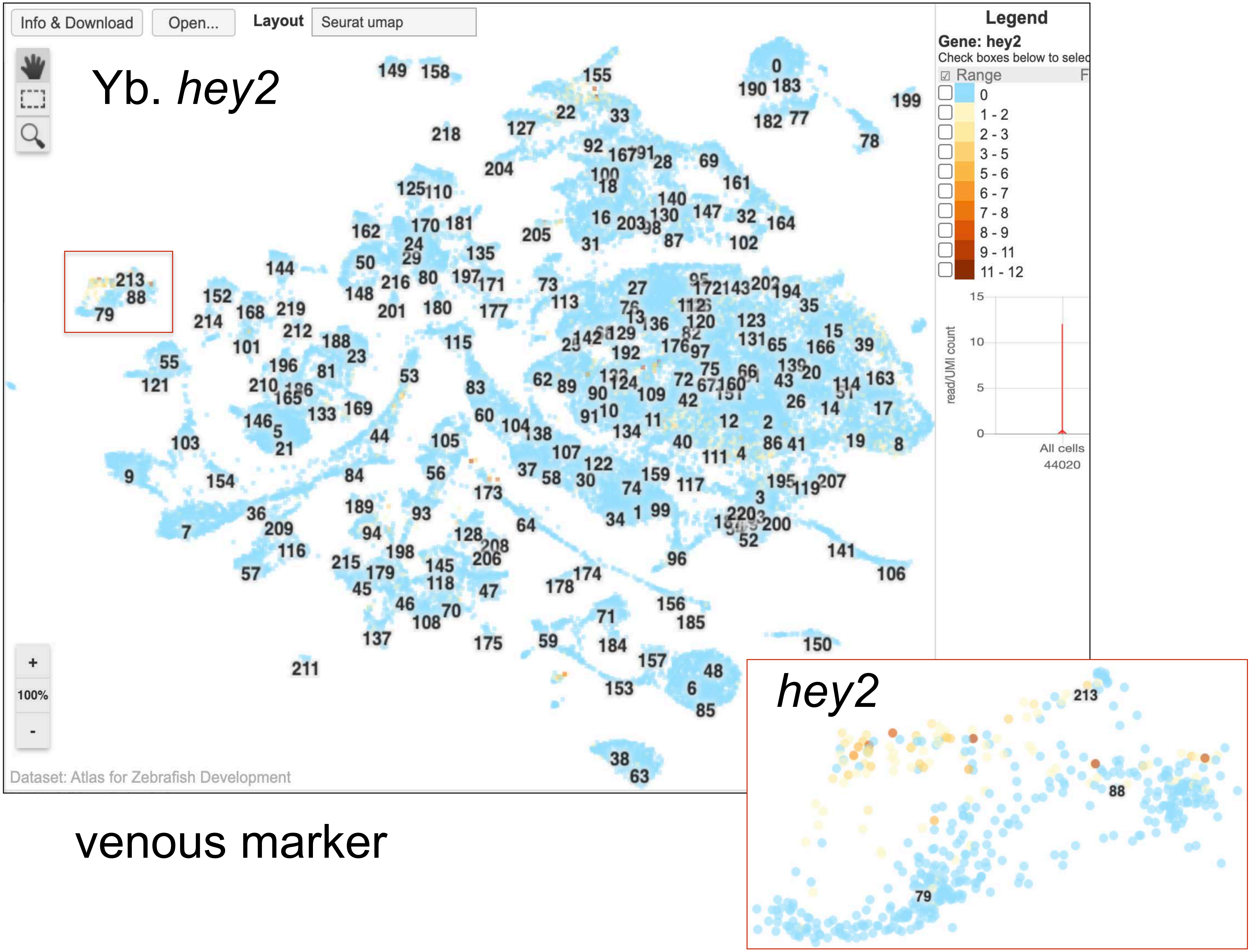


venous marker

Biology Open • Supplementary information

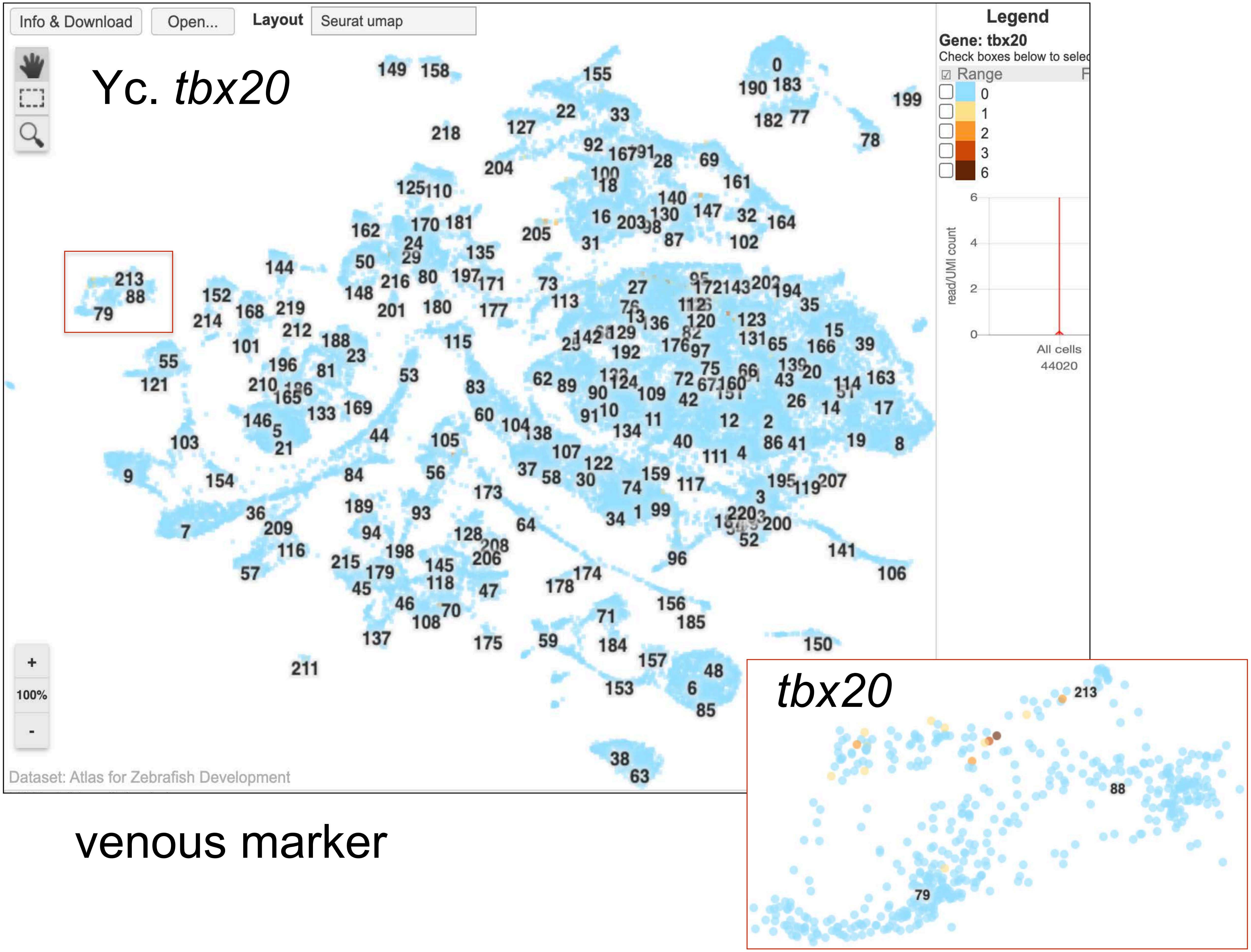


venous marker

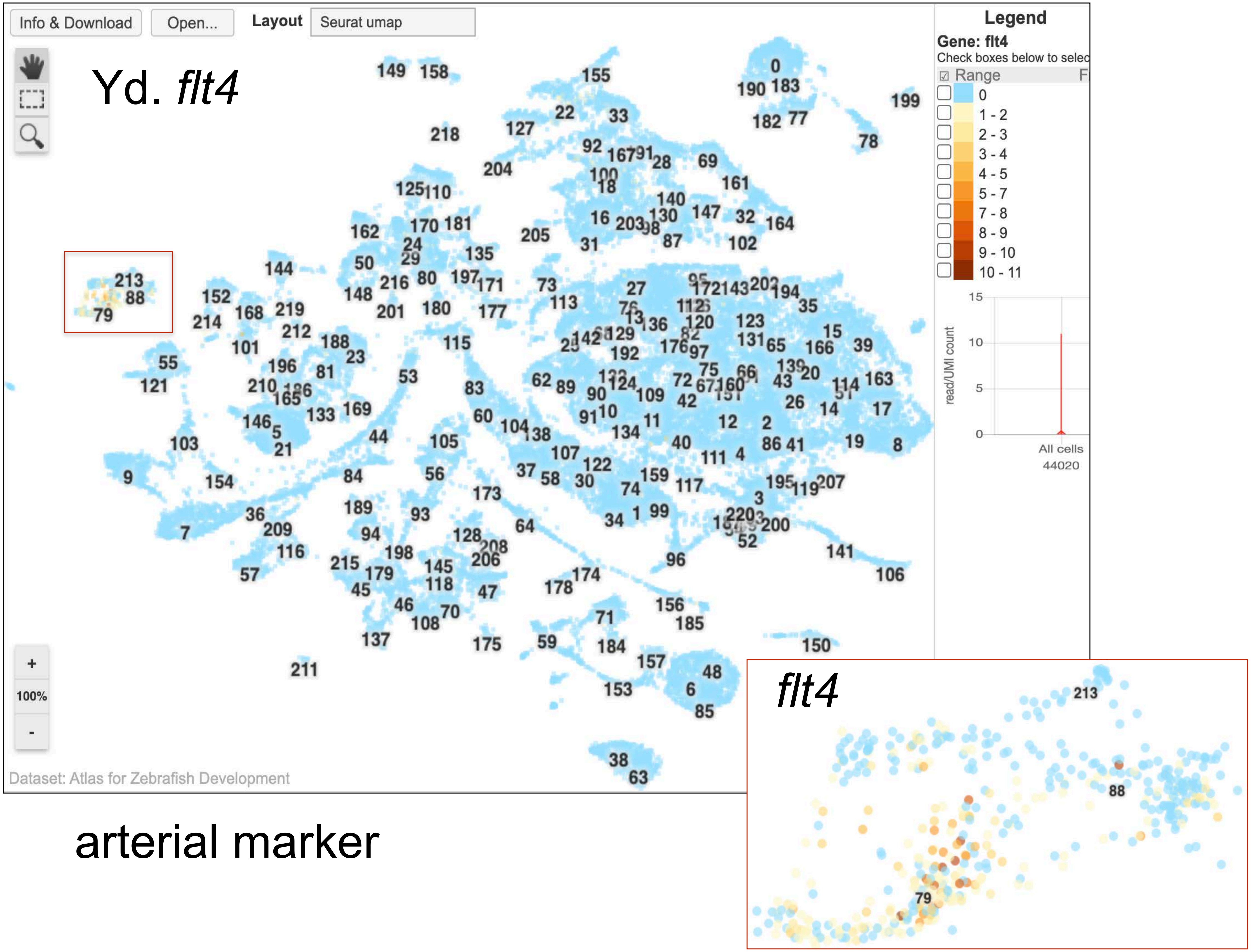


arterial marker

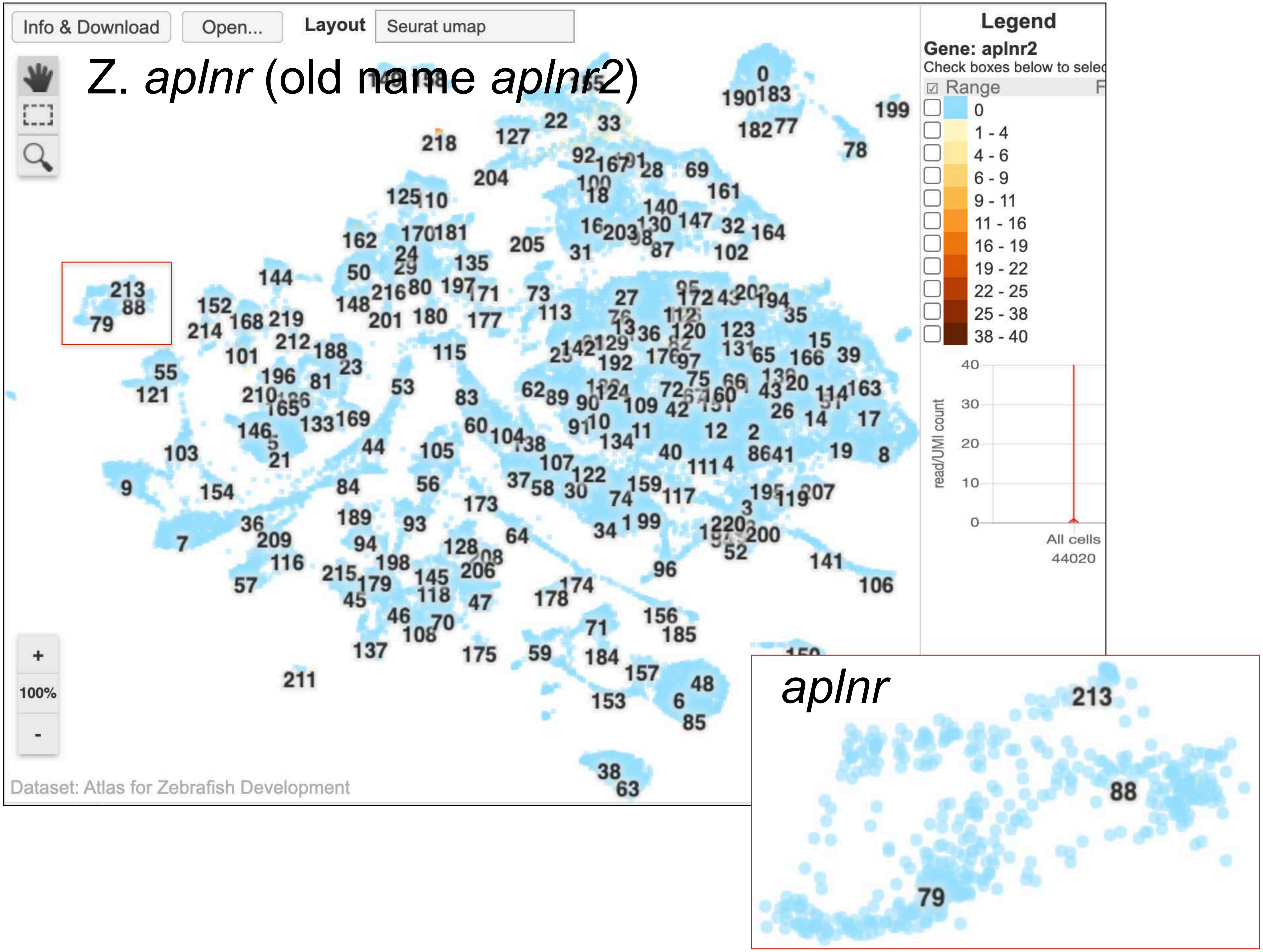


Biology Open • Supplementary information

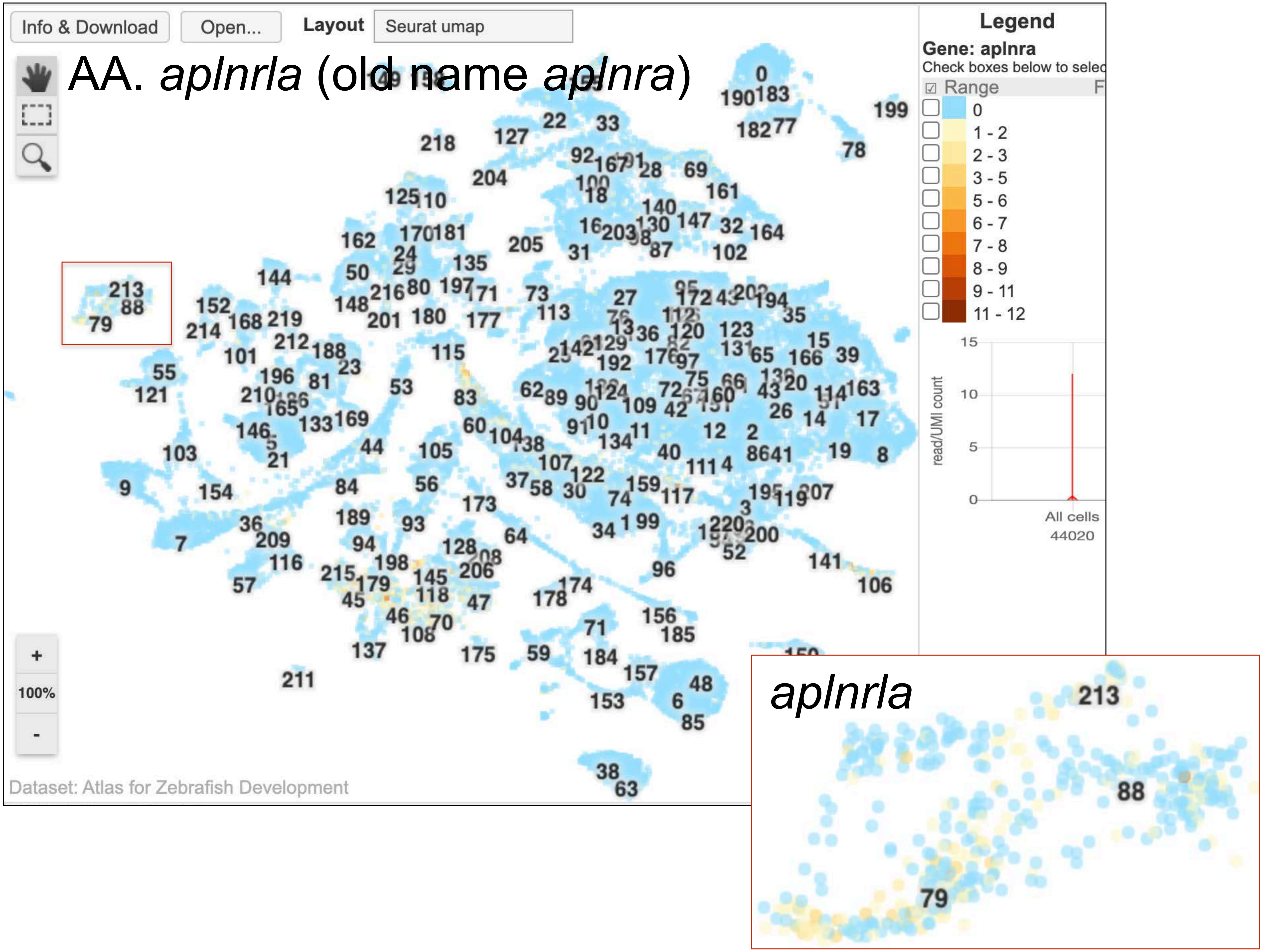
AA. aplnrla (old name aplnra)
Info & Download
Open...
Layout
Seurat umap
Legend
Gene: aplnra
Check boxes below to selec
Range
0
1 - 2
2 - 3
3 - 5
5 - 6
6 - 7
7 - 8
8 - 9
9 - 11
11 - 12
read/UMI count
All cells
44020
100%
Dataset: Atlas for Zebrafish Development
aplnrla
213
88
79

BB. *aplnrlb* (old name: *aplnrb*)

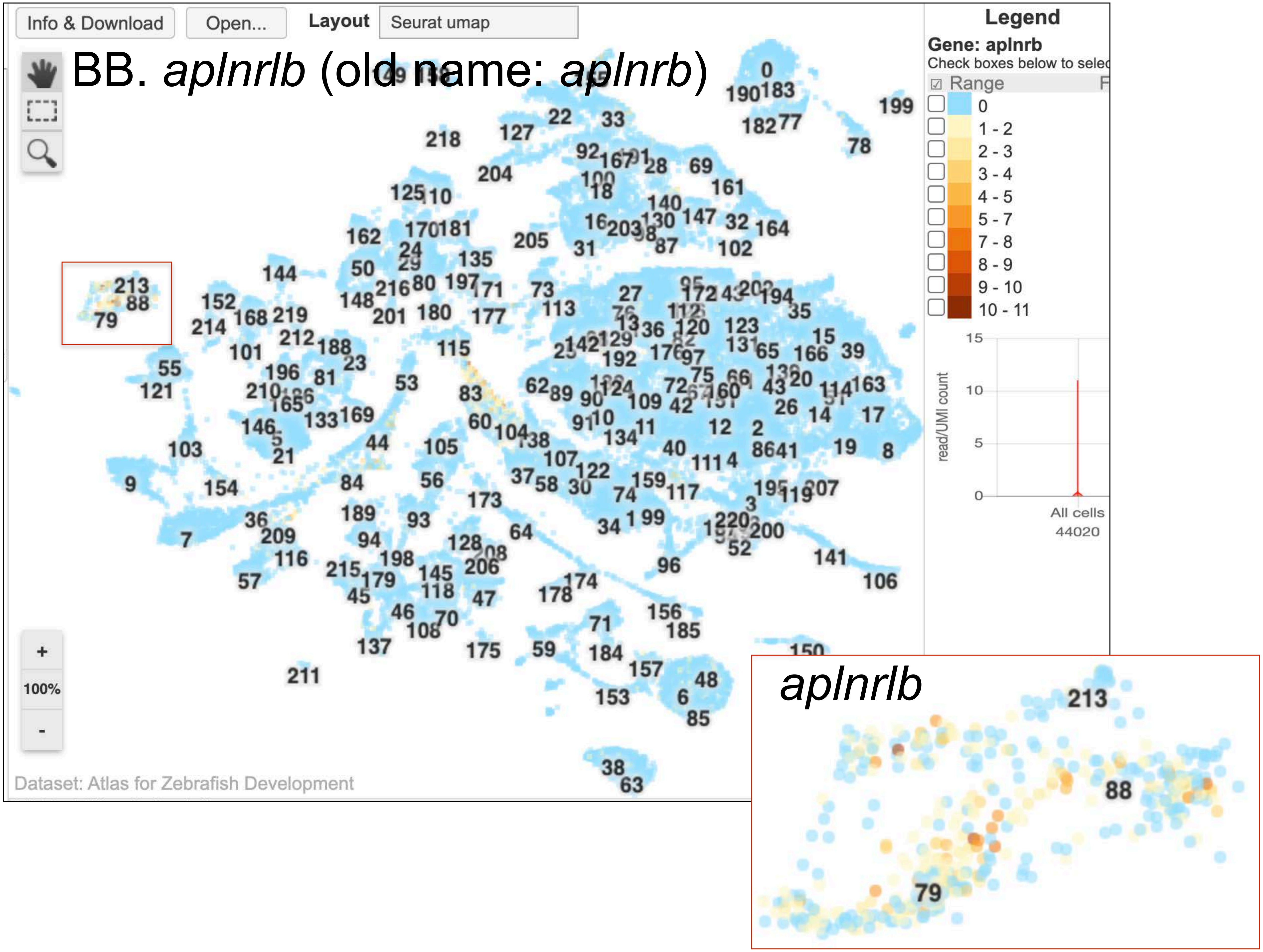


Biology Open • Supplementary information

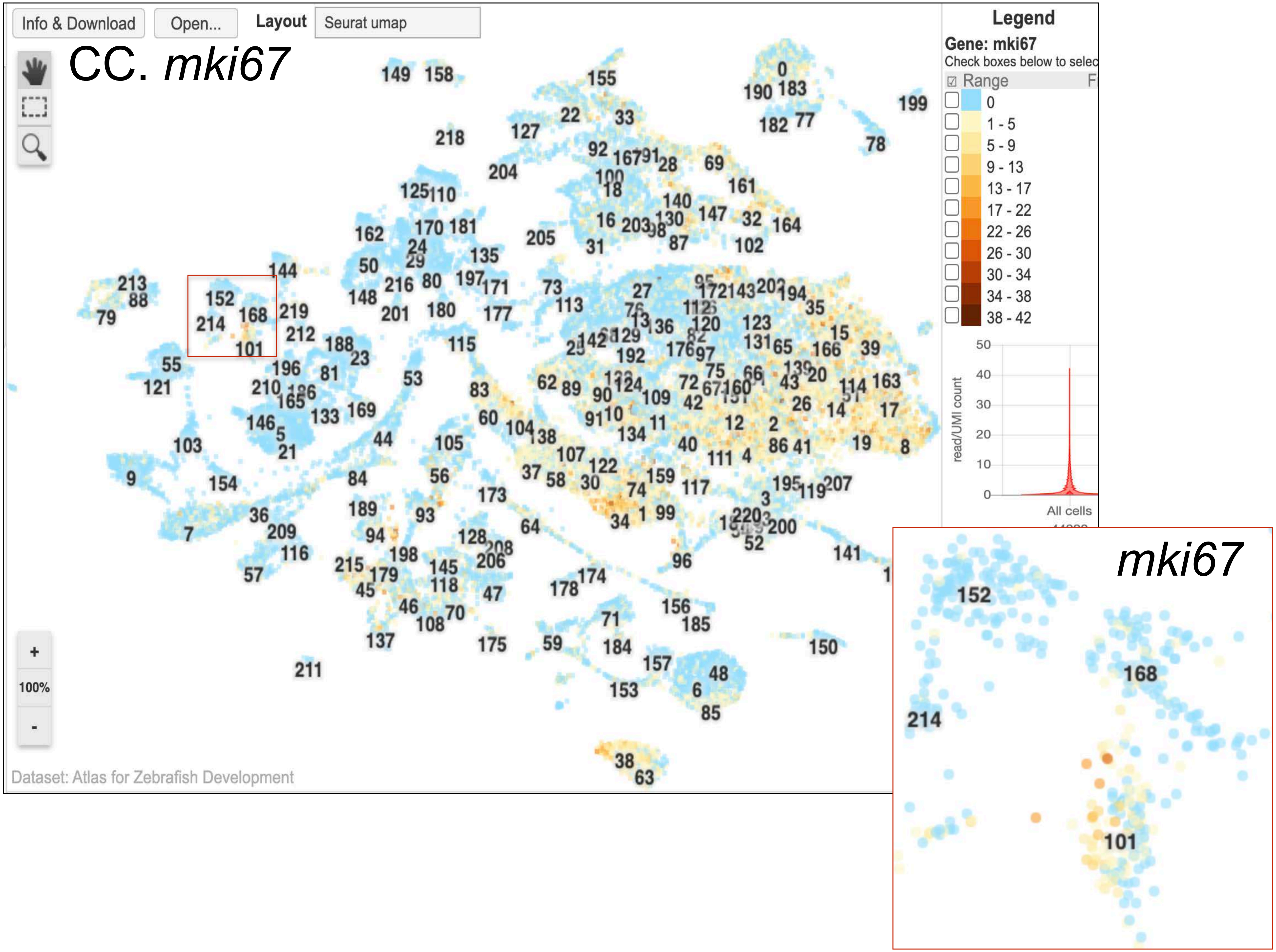
Info & Download
Open...
Layout
Seurat umap
CC. *mki67*
Legend
Gene: mki67
Check boxes below to selec
Range
0
1 - 5
5 - 9
9 - 13
13 - 17
17 - 22
22 - 26
26 - 30
30 - 34
34 - 38
38 - 42
read/UMI count
All cells
100%
Dataset: Atlas for Zebrafish Development
*mki67*
152
168
214
101

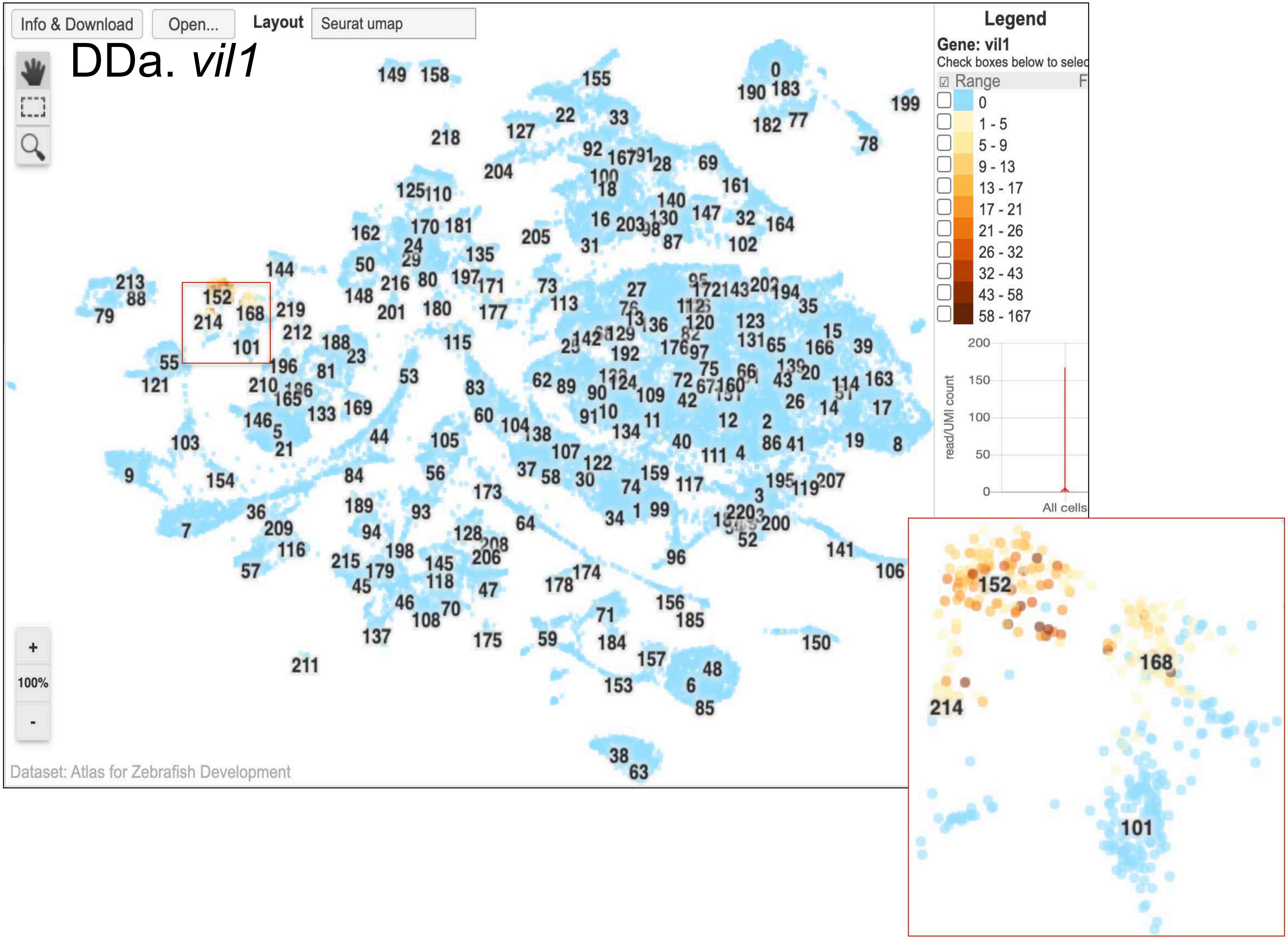


Biology Open • Supplementary information

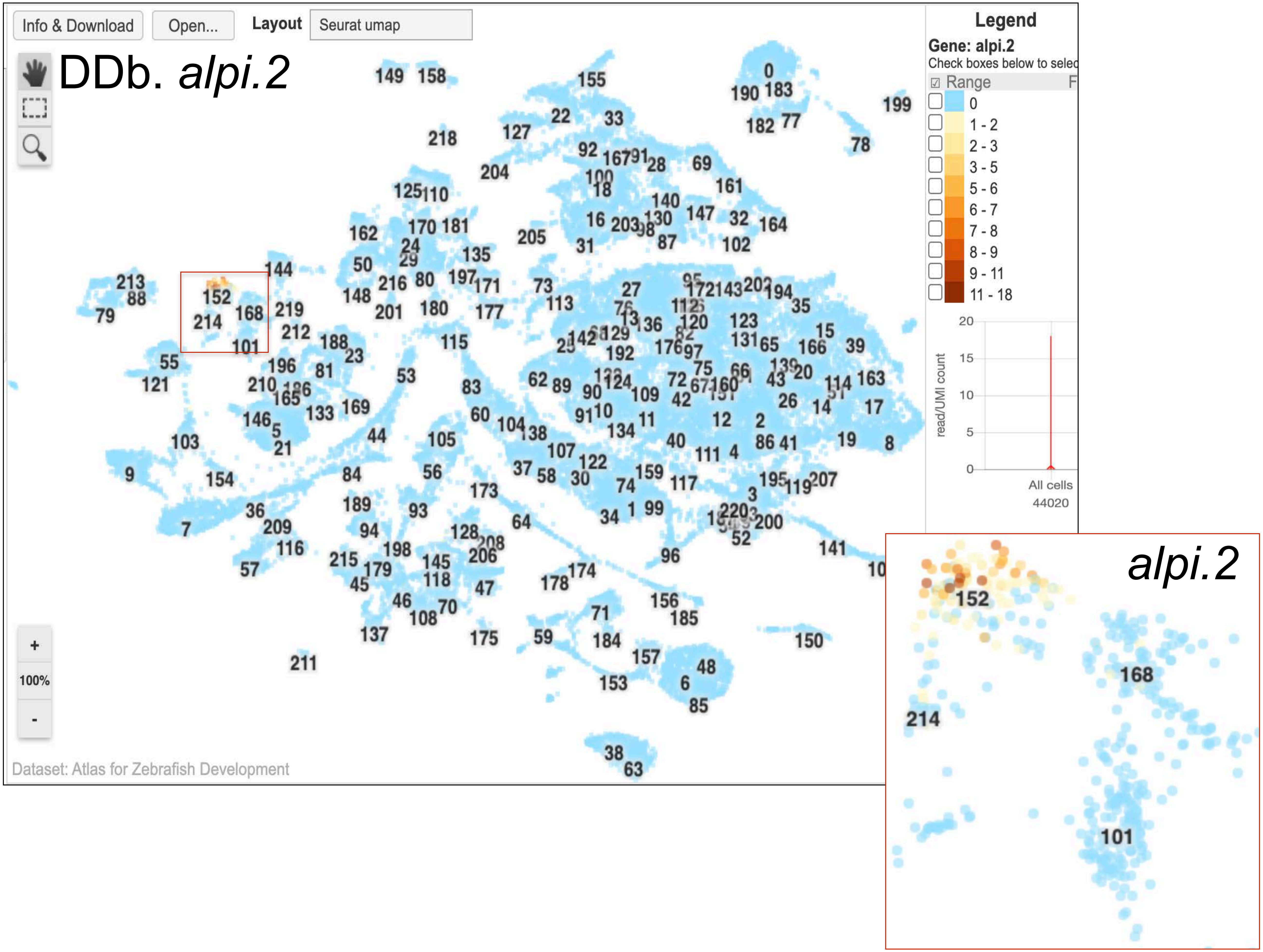
DDb. *alpi.2*
Info & Download
Open...
Layout
Seurat umap
Legend
Gene: alpi.2
Range
0
1 - 2
2 - 3
3 - 5
5 - 6
6 - 7
7 - 8
8 - 9
9 - 11
11 - 18
read/UMI count
All cells
44020
Dataset: Atlas for Zebrafish Development
*alpi.2*
152
168
214
101

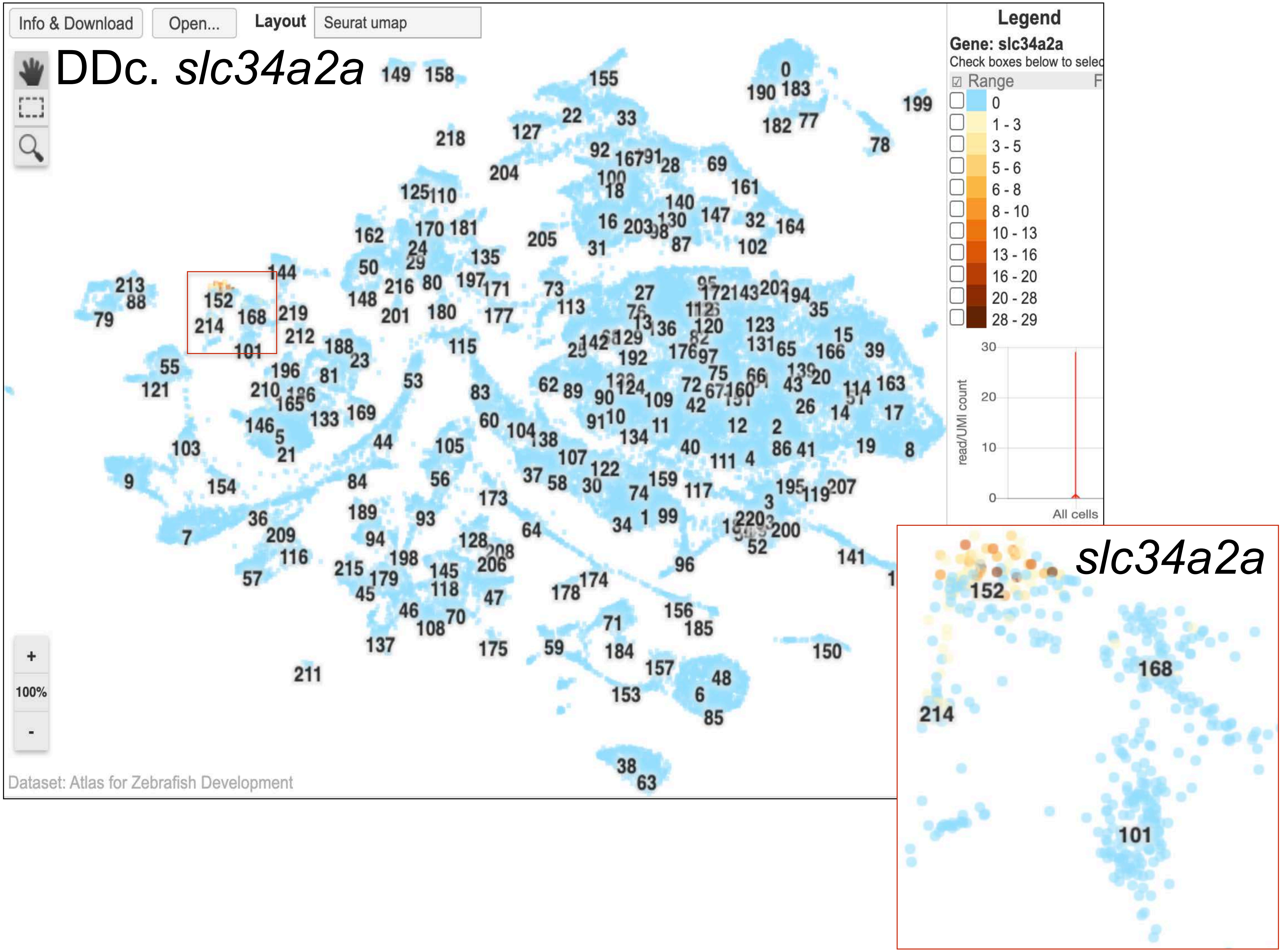


Biology Open • Supplementary information

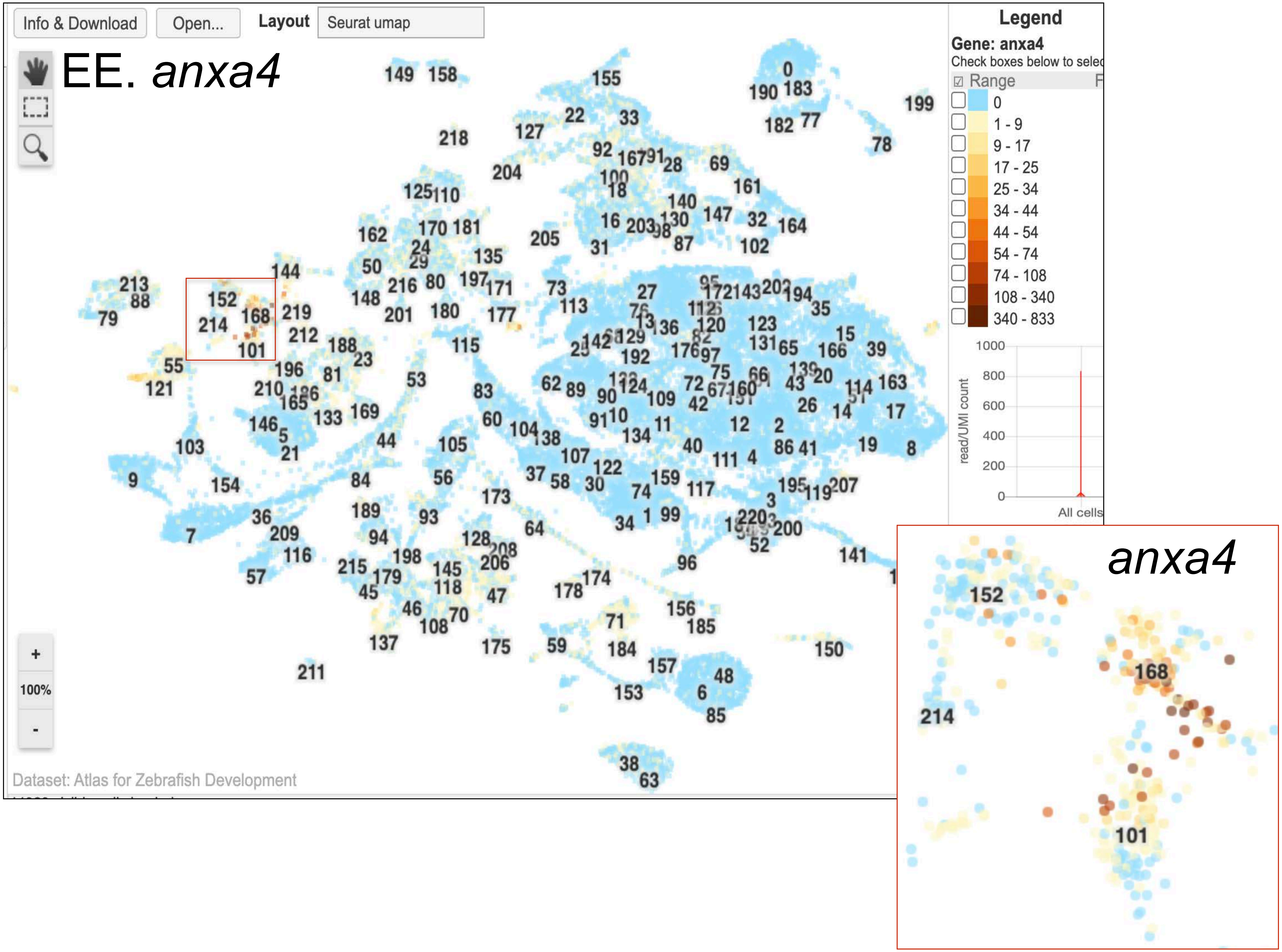


Biology Open • Supplementary information

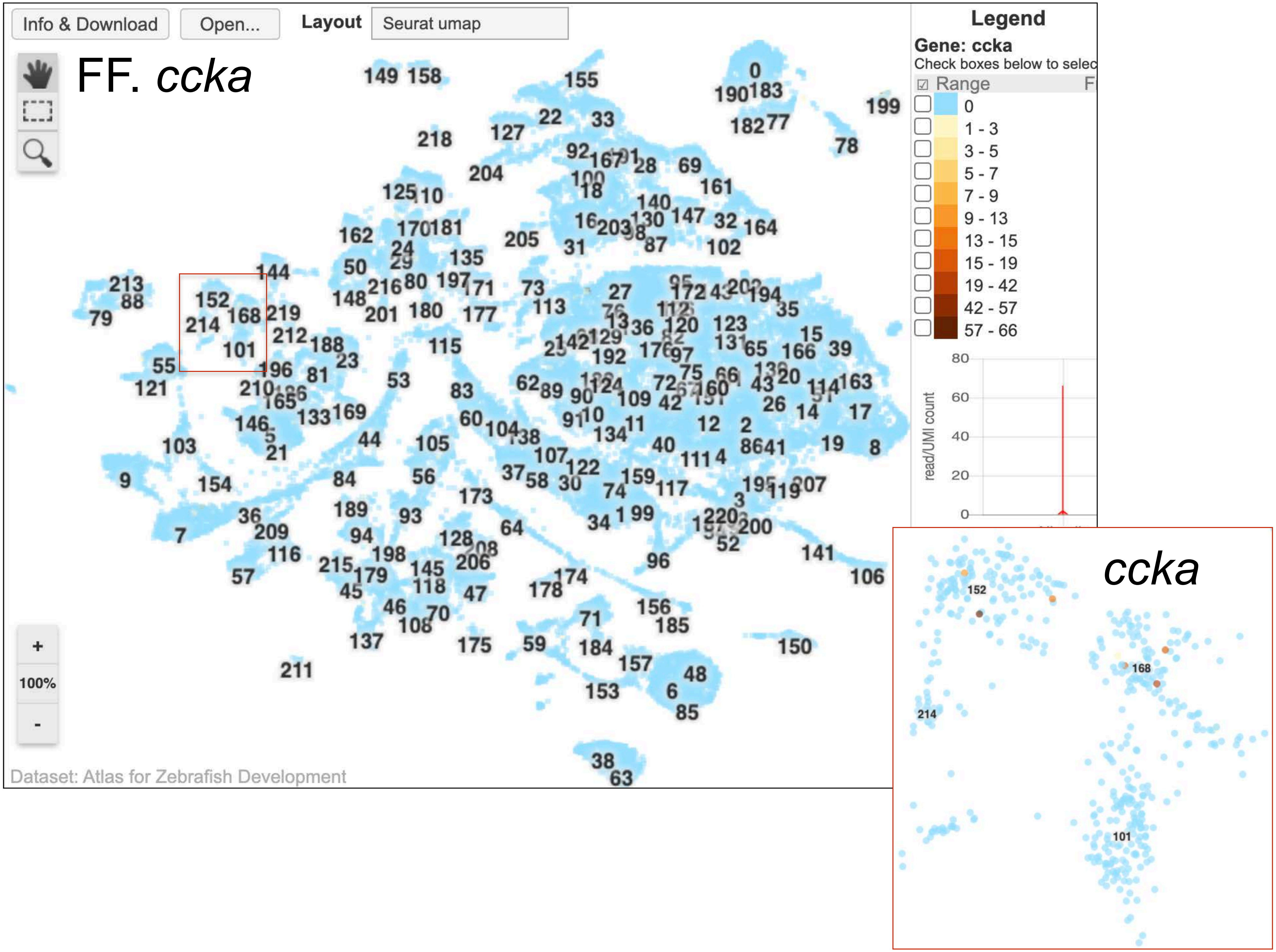


Biology Open • Supplementary information

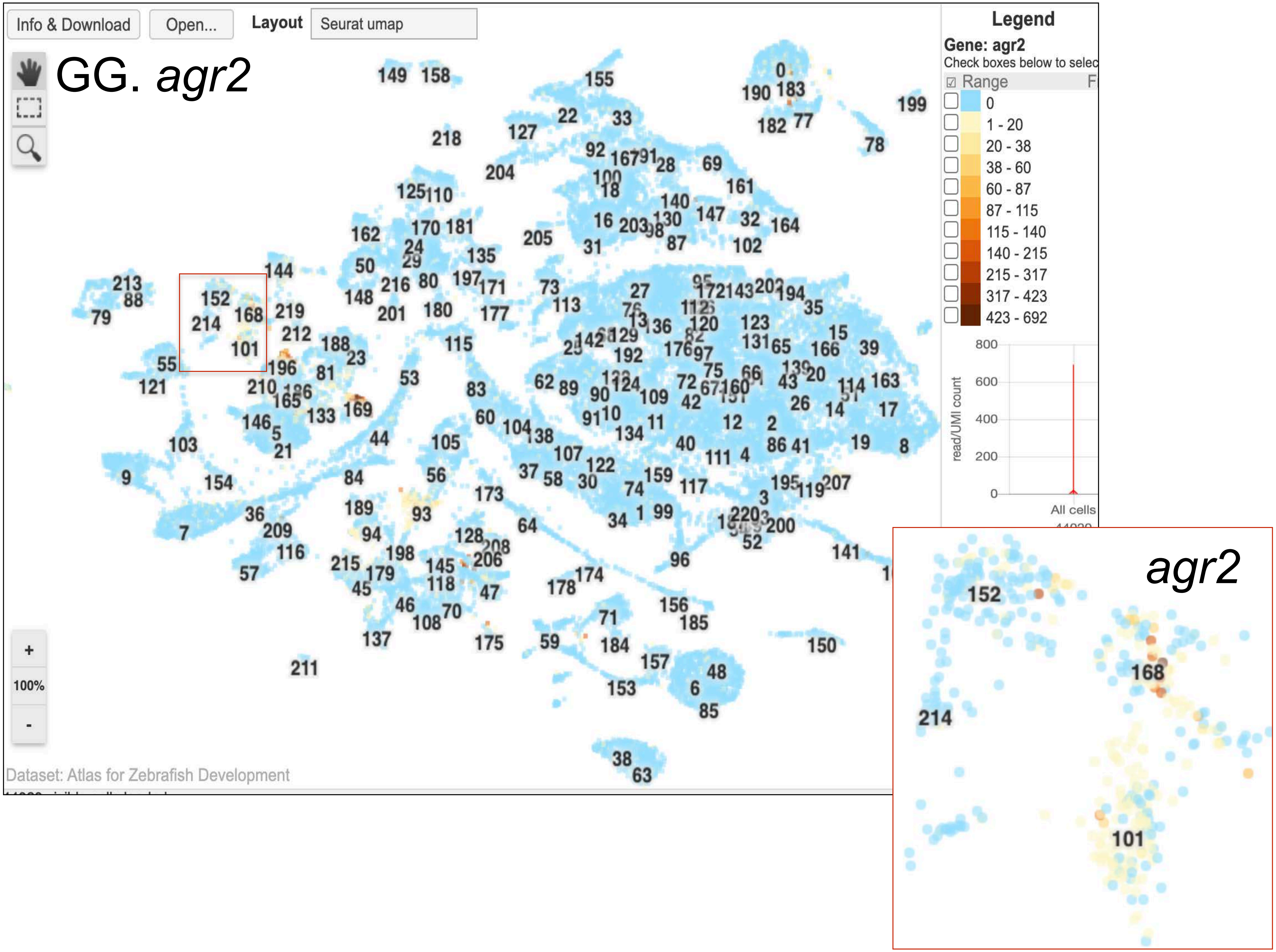


Biology Open • Supplementary information

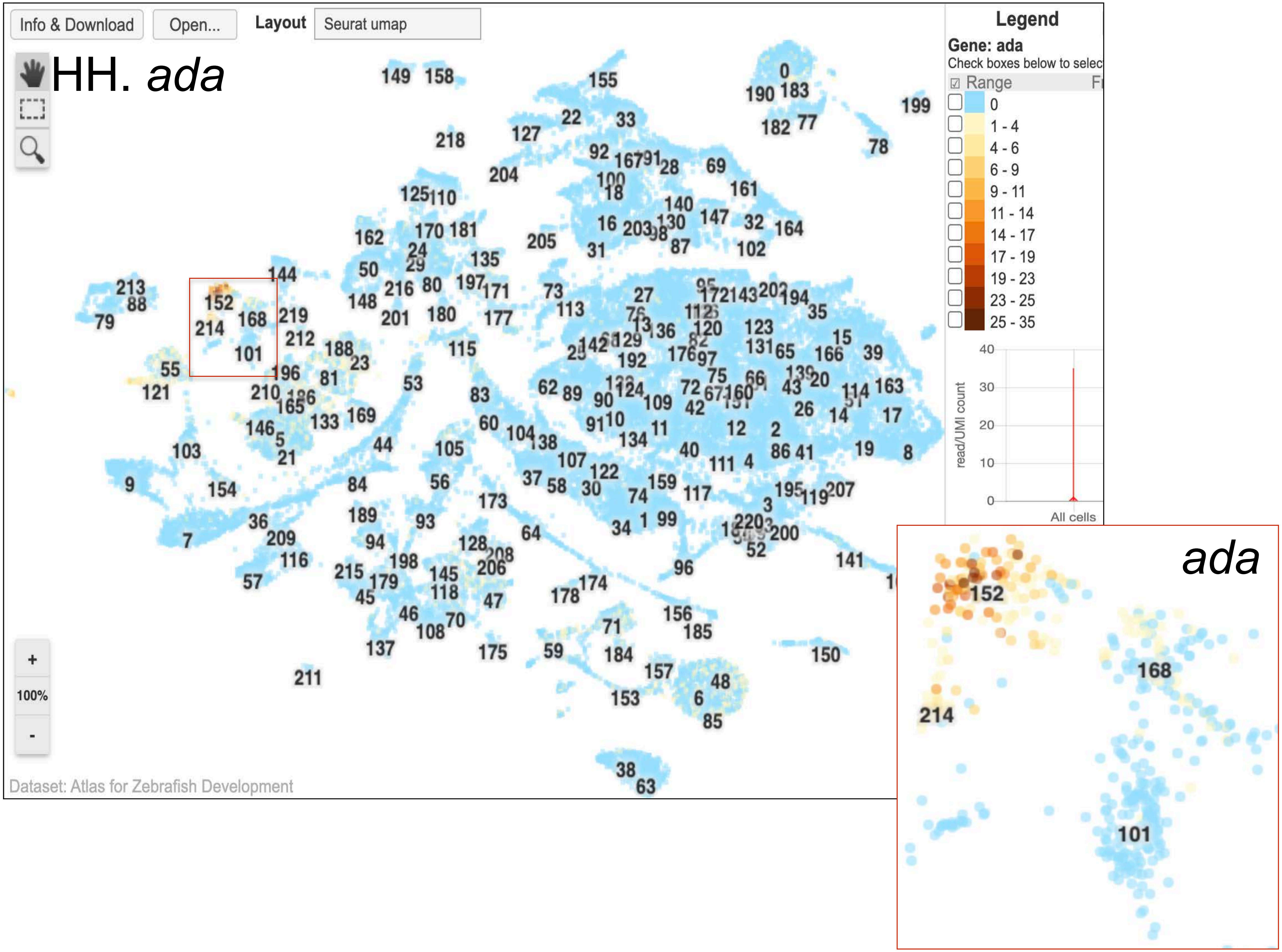
Info & Download
Open...
Layout
Seurat umap
HH. ada
Legend
Gene: ada
Check boxes below to selec
Range
0
1 - 4
4 - 6
6 - 9
9 - 11
11 - 14
14 - 17
17 - 19
19 - 23
23 - 25
25 - 35
read/UMI count
40
30
20
10
0
All cells
100%
Dataset: Atlas for Zebrafish Development
ada
152
168
214
101

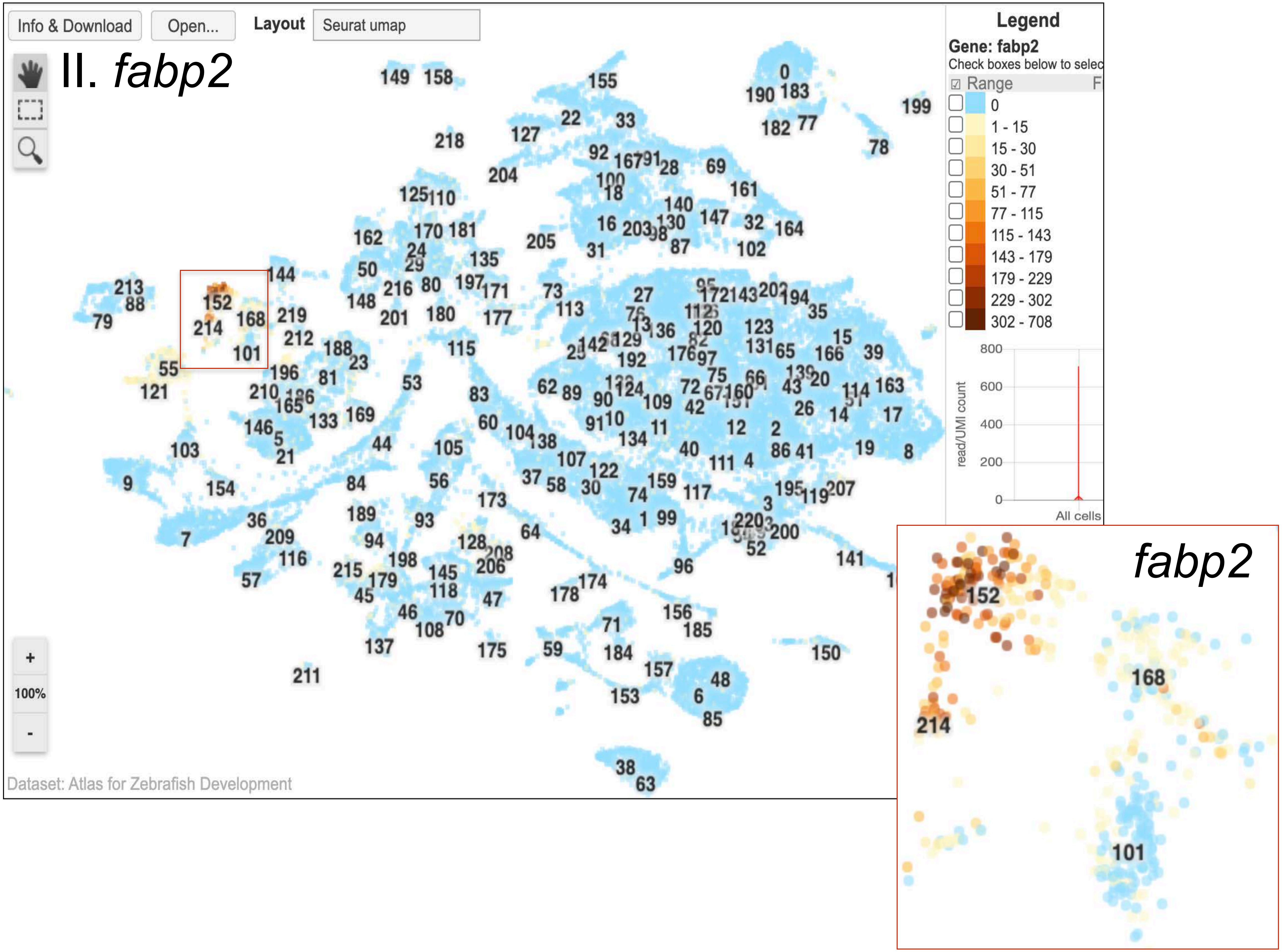


Biology Open • Supplementary information

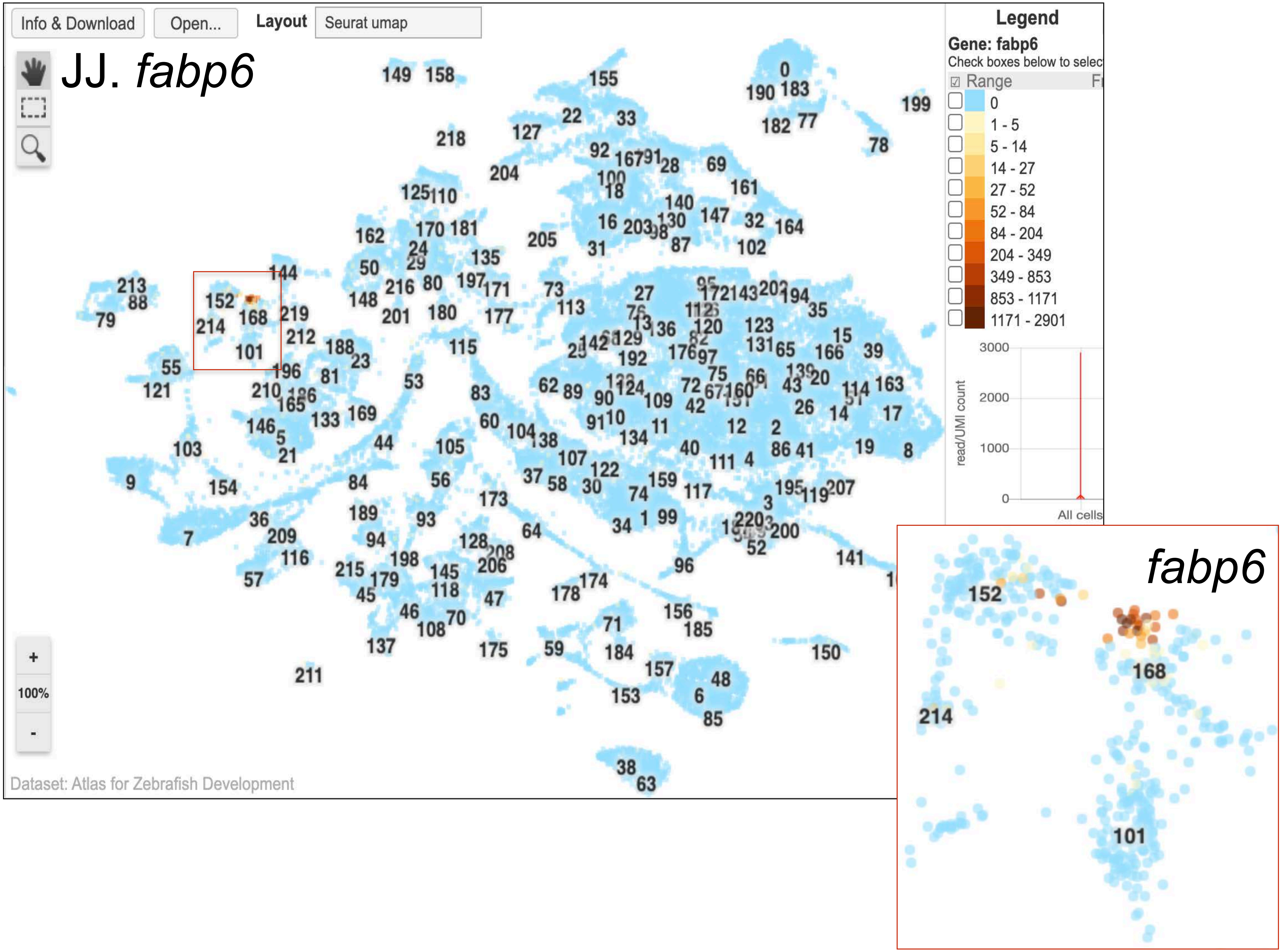


Biology Open • Supplementary information

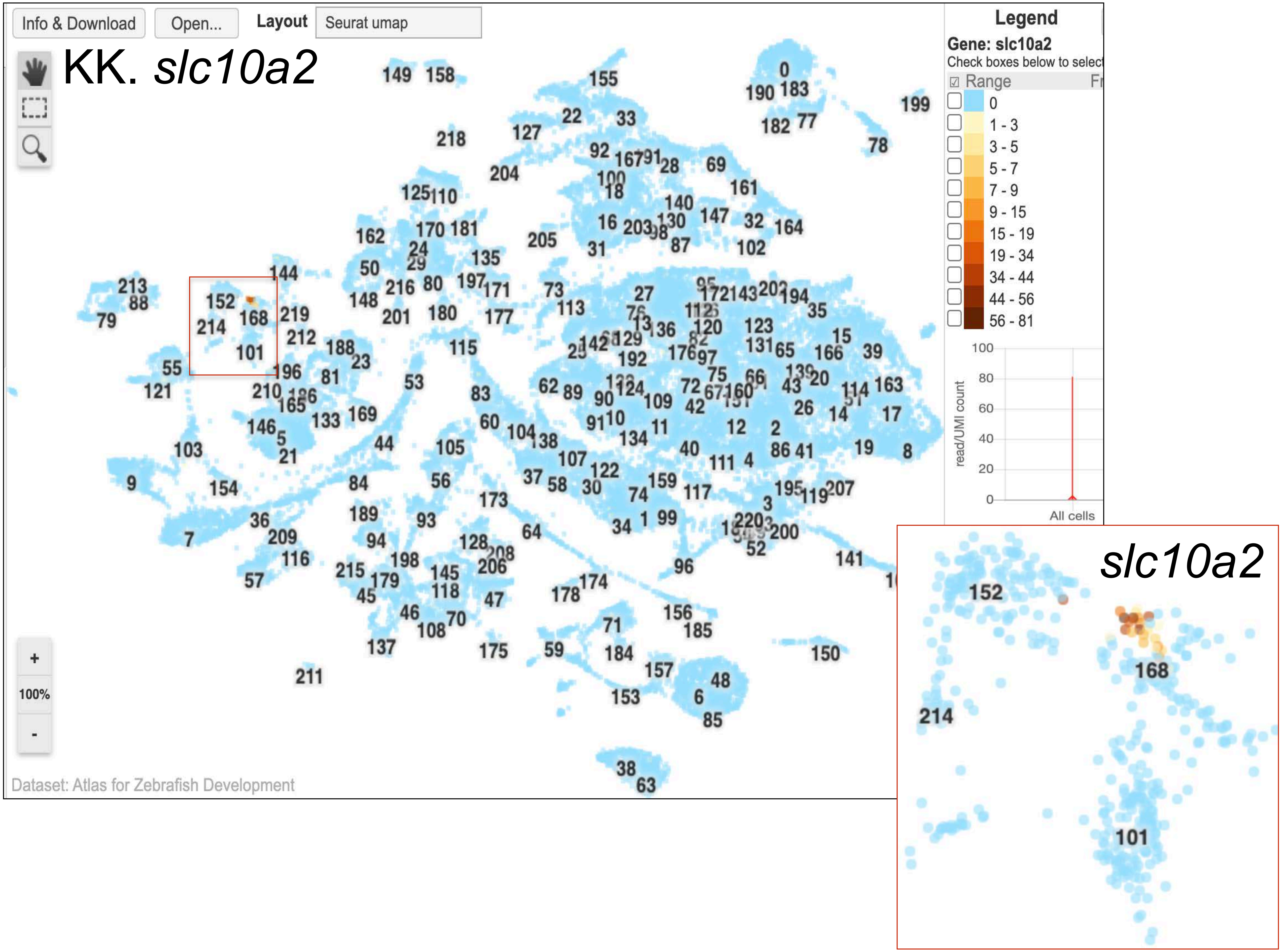


Biology Open • Supplementary information

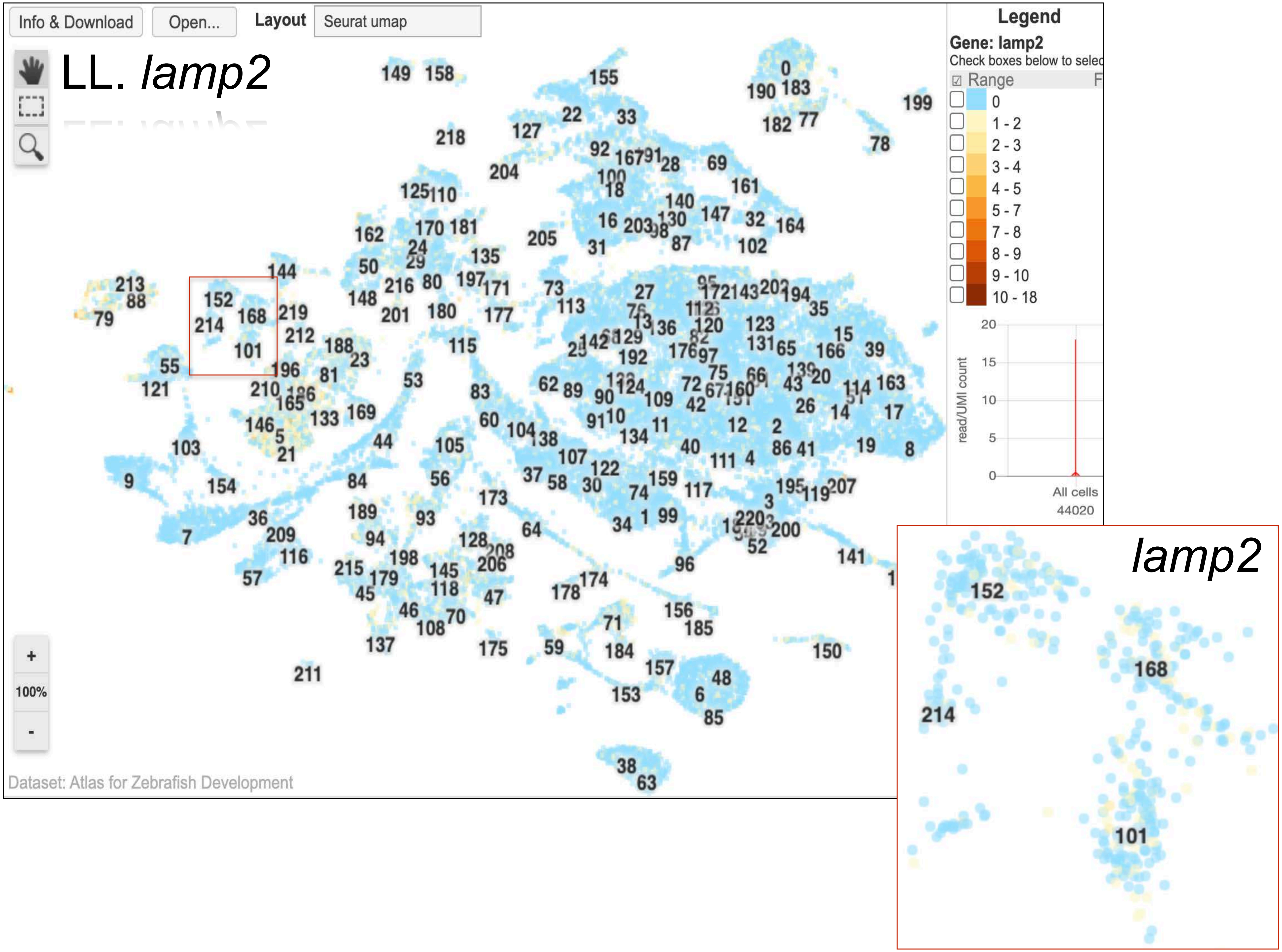


Biology Open • Supplementary information

Supplementary Table 1. Angiotensin sequences with Accession numbers for various vertebrate taxa.

| | *Species* | Common name | Taxon | Antiotensin | Accession Number |
|---|---|---|---|---|---|
| Sarcopterygii | *Myotis lucifugus* | little brown bat | Vespertilioniodea | DRLYIHPFHM | XP_006106579.1 |
| | *Phyllostomus discolor* | pale spear nosed bat | Noctilionoidea | DRVYVHPFHL | XP_028387106.1 |
| | *Hipposideros armiger* | great roundleaf bat | Yinpterochiroptera | DRVYIHPFHL | XP_019521404.1 |
| | *Canis lupus* | dog | Carnivora | DRVYIHPFHL | XP_025304321.1 |
| | *Equus caballus* | horse | Perissodactyla | DRVYIHPFHL | XP_005602671.1 |
| | *Ovis aries* | sheep | Artiodactyla | DRVYIHPFHL | XP_011960976.2 |
| | *Capra hircus* | goat | Artiodactyla | DRVYVHPFHL | XP_005699430.1 |
| | *Bos taurus* | cow | Artiodactyla | DRVYVHPFHL | XP_027386596.1 |
| | *Bison bison* | bison | Artiodactyla | DRVYVHPFHL | XP_010852803. |
| | *Cervus hanglu yarkandensis* | Yarkland deer | Artiodactyla | DRVYVHPFHL | KAF4018817.1 |
| | *Kogia sima* | dwarf sperm whale | Artiodactyla | DRVYVHPFHL | AGV55446.1 |
| | *Odocoileus virginianus* | white tailed deer | Artiodactyla | DRVYVHPFHL | XP_020725477.1 |
| | *Heterocephalus glaber* | naked mole rat | Guinea pig-related | DRVYIHPFHL | EHB11546.1 |
| | *Marmota marmota* | marmot | Squirril-related | DRVYVHPFHL | XP_015359803 |
| | *Urocitellus parryii* | Arctic ground squirrel | Squirril-related | DRVYVHPFHL | XP_026268640.1 |
| | *Castor canadensis* | beaver | Castorimorpha | DRVYIHPFHL | XP_020042418.1 |
| | *Jaculus jaculus* | jerboa | Dipodidae | DRVYIHPFHL | XP_004659683 |
| | *Mus musculus* | mouse | Muridae | DRVYIHPFHL | NP_031454.4 |
| | *Oryctolagus cuniculus* | rabbit | Lagomorpha | DRVYIHPFHL | XP_008266492.1 |
| | *Homo sapiens* | human | Primates | DRVYIHPFHL | NP_000020.1 |
| | *Saimiri boliviensis* | squirrel monkey | Primates | DRVYVHPFHL | XP_003940820.1 |
| | *Galeopterus variegatus* | lemur | Dermoptera | DRVYIHPFHL | XP_008588191.1 |
| | *Tupaia chinensis* | tree shrew | Scandentia | DRVYIHPFHL | ELW68162.1 |
| | *Loxodonta africana* | elephant | Afrotheria | DRVYIHPFHL | XP_010589414.1 |
| | *Monodelphis domestica* | opposum | Marsupialia | DRVYVHPFHL | XP_016285745.1 |
| | *Ornithorhynchus anatinus* | platypus | Monotremata | DRVYVHPFHF | XP_028903195.1 |
| | *Gallus gallus* | chicken | Dinosauria | DRVYVHPFSL | XP_004935550.1 |
| | *Alligator mississippiensis* | alligator | Crocodylomorpha | DRVYVHPFAL | AAB27391.1 |
| | *Ophiophagus hannah* | king cobra | Squamata | DRVYVHPFYL | ETE65448.1 |
| | *Bothrops jararaca* | pit viper | Serpentes | DRVYVHPFYL | Q10581.1 |
| | *Python bivittatus* | Burmese python | Serpentes | DRVYVHPFFL | XP_007423730.1 |

Biology Open • Supplementary information

| | | | | | |
|---|---|---|---|---|---|
| | *Gekko japonicus* | gekko | Gekkota | DRVYVHPFHL | XP_015276930.1 |
| | *Chelonia mydas* | green sea turtle | Testudines | DRVYVHPFHL | XP_007071121.1 |
| | *Xenopus tropicalis* | western clawed frog | Ampnibia | NRVYIHPFNL | AAI55494.1 |
| | *Rhinatrema bivittatum* | two lined caecilian | Amphibian | NRVYVHPFHF | XP_029450048.1 |
| | *Latimeria chalumnae* | coelacanth | Coelacanthomorpba | NRVYVHPFNL | XP_005994004.1 |
| Actinopterygii | *Erpetoichthys calabaricus* | reedfish | Polypteriiformes | NRVYVHPFKL | XP_028653133.1 |
| | *Acipenser ruthenus* | sterlet sturgeon | Acipenseriformes | NRVYVHPFNL | RXM35492.1 |
| | *Lepisosteus oculatus* | spotted gar | Lepisosteiformes | NRVYVHPFKL | XP_006638515.1 |
| | *Paramormyrops kingsleyae* | elephantfish | Osteoglossiformes | NRVYVHPFNL | XP_023689503.1 |
| | *Scleropages formosus* | arowana | Osteoglossiformes | NRVYVHPFSL | XP_018604956.2 |
| | *Clupea harengus* | herring | Clupeiformes | NRVYVHPFSL | XP_012669781.1 |
| | *Danio rerio* | zebrafish | Cypriniformes | NRVYVHPFNL | AAH95585.1 |
| | *Astyanax mexicanus* | mexican tetra | Cypriniformes | NRVYVHPFYL | XP_007256132.2 |
| | *Ictalurus punctata* | catfish | Cypriniformes | NRVYIHPFSL | XP_017319957.1 |
| | *Esox lucius* | Northern pike | Esociformes | NRVYVHPFHL | XP_012989289.1 |
| | *Plecoglossus altivelis* | ayu | Osmeriformes | NRVYVHPFNL | CAP57927.1 |
| | *Gadus morhua* | Atlantic cod | Gadiformes | NRVYIHPFYL | XP_030235081. |
| | *Periophthalmus modestus* | mudskipper | Gobiiformes | NRVYVHPFNL | BAZ91802.1 |
| | *Hippocampus comes* | seahorse | Syngnathiformes | NRVYIHPFHL | XP_019715980. |
| | *Scophthalmus maximus* | turbot | Peuronectiiformes | NRVYIHPFQL | AWP13832.1 |
| | *Oreochromis niloticus* | tilapia | Cichlidiformes | NRVYVHPFYL | XP_003438333.1 |
| | *Oryzias latipes* | medaka | Beloniformes | NRVYVHPFYL | XP_004077120.1 |
| | *Oplegnathus fasciatus* | knife jaw | Centrarchiformes | NRVYVHPFHL | AEJ91608.1 |
| | *Labrus bergylta* | wrasse | Labriformes | NRVYIHPFHL | XP_020494818.1 |
| | *Sparus aurata* | sea bream | Spariformes | NRVYIHPFHL | XP_030296777.1 |
| | *Takifugu rubripes* | pufferfish | Tetraodontiformes | NRVYVHPFSL | XP_003963811.1 |
| Chondrichthyes | *Triakis scyllium* | houndshark | Chondrichthyes | NRPYIHPFQL | BAH10143.1 |
| | *Hemitrygon akajei* | stingray | Chondrichthyes | DRPYIHPFFL | BAH10144.1 |
| | *Leucoraja erinacea* | little skate | Chondrichthyes | YRPYIHPFSL | BAH10146.1 |
| | *Amblyraja radiata* | thorny skate | Chondrichthyes | YRPYIHPFSL | XP_032877724.1 |
| Agnathans | *Petromyzon marinus* | sea lamprey | Petromyzontiformes | DRPYMQPFHL | ADK22836.1 |
| | *Lampetra fluviatilis* | river lamprey | Petromyzontiformes | ERPYMQPFHL | CAV29466.1 |
| | *Lethenteron camtschaticum* | arctic lamprey | Petromyzontiformes | ERPYMQPFHL | ADK56280.1 |

Biology Open • Supplementary information

## REFERENCES

**Batlle, D., Wysocki, J. and Satchell, K.** (2020). Soluble angiotensin-converting enzyme 2: a potential approach for coronavirus infection therapy? *Clin Sci (Lond)* **134**, 543-545.

**Burrell, L. M., Risvanis, J., Kubota, E., Dean, R. G., MacDonald, P. S., Lu, S., Tikellis, C., Grant, S. L., Lew, R. A., Smith, A. I. et al.** (2005). Myocardial infarction increases ACE2 expression in rat and humans. *Eur Heart J* **26**, 369-75; discussion 322-4.

**Camargo, S. M., Singer, D., Makrides, V., Huggel, K., Pos, K. M., Wagner, C. A., Kuba, K., Danilczyk, U., Skovby, F., Kleta, R. et al.** (2009). Tissue-specific amino acid transporter partners ACE2 and collectrin differentially interact with hartnup mutations. *Gastroenterology* **136**, 872-82.

**Ciaglia, E., Vecchione, C. and Puca, A. A.** (2020). COVID-19 Infection and Circulating ACE2 Levels: Protective Role in Women and Children. *Front Pediatr* **8**, 206.

**De Mota, N., Reaux-Le Goazigo, A., El Messari, S., Chartrel, N., Roesch, D., Dujardin, C., Kordon, C., Vaudry, H., Moos, F. and Llorens-Cortes, C.** (2004). Apelin, a potent diuretic neuropeptide counteracting vasopressin actions through inhibition of vasopressin neuron activity and vasopressin release. *Proc Natl Acad Sci U S A* **101**, 10464-9.

**Dray, C., Knauf, C., Daviaud, D., Waget, A., Boucher, J., Buleon, M., Cani, P. D., Attane, C., Guigne, C., Carpene, C. et al.** (2008). Apelin stimulates glucose utilization in normal and obese insulin-resistant mice. *Cell Metab* **8**, 437-45.

**Ferrario, C. M., Jessup, J., Chappell, M. C., Averill, D. B., Brosnihan, K. B., Tallant, E. A., Diz, D. I. and Gallagher, P. E.** (2005). Effect of angiotensin-converting enzyme inhibition and angiotensin II receptor blockers on cardiac angiotensin-converting enzyme 2. *Circulation* **111**, 2605-10.

**Fyhrquist, F. and Saijonmaa, O.** (2008). Renin-angiotensin system revisited. *J Intern Med* **264**, 224-36.

**Hoffmann, M., Kleine-Weber, H., Schroeder, S., Kruger, N., Herrler, T., Erichsen, S., Schiergens, T. S., Herrler, G., Wu, N. H., Nitsche, A. et al.** (2020). SARS-CoV-2 Cell Entry Depends on ACE2 and TMPRSS2 and Is Blocked by a Clinically Proven Protease Inhibitor. *Cell* **181**, 271-280 e8.

**Holmes, R. S., Spradling-Reeves, K. D. and Cox, L. A.** (2017). Mammalian Glutamyl Aminopeptidase Genes (ENPEP) and Proteins: Comparative Studies of a Major Contributor to Arterial Hypertension. *J Data Mining Genomics Proteomics* **8**.

**Jiang, F., Yang, J., Zhang, Y., Dong, M., Wang, S., Zhang, Q., Liu, F. F., Zhang, K. and Zhang, C.** (2014). Angiotensin-converting enzyme 2 and angiotensin 1-7: novel therapeutic targets. *Nat Rev Cardiol* **11**, 413-26.

**Kasai, A., Shintani, N., Oda, M., Kakuda, M., Hashimoto, H., Matsuda, T., Hinuma, S. and Baba, A.** (2004). Apelin is a novel angiogenic factor in retinal endothelial cells. *Biochem Biophys Res Commun* **325**, 395-400.

**Kuba, K., Imai, Y., Rao, S., Gao, H., Guo, F., Guan, B., Huan, Y., Yang, P., Zhang, Y., Deng, W. et al.** (2005). A crucial role of angiotensin converting enzyme 2 (ACE2) in SARS coronavirus-induced lung injury. *Nat Med* **11**, 875-9.

**Kuster, G. M., Pfister, O., Burkard, T., Zhou, Q., Twerenbold, R., Haaf, P., Widmer, A. F. and Osswald, S.** (2020). SARS-CoV2: should inhibitors of the renin-angiotensin system be withdrawn in patients with COVID-19? *Eur Heart J*.

**Lambert, D. W., Yarski, M., Warner, F. J., Thornhill, P., Parkin, E. T., Smith, A. I., Hooper, N. M. and Turner, A. J.** (2005). Tumor necrosis factor-alpha convertase (ADAM17) mediates regulated ectodomain shedding of the severe-acute respiratory syndrome-coronavirus (SARS-CoV) receptor, angiotensin-converting enzyme-2 (ACE2). *J Biol Chem* **280**, 30113-9.

**Massiera, F., Seydoux, J., Geloen, A., Quignard-Boulange, A., Turban, S., Saint-Marc, P., Fukamizu, A., Negrel, R., Ailhaud, G. and Teboul, M.** (2001). Angiotensinogen-deficient mice exhibit impairment of diet-induced weight gain with alteration in adipose tissue development and increased locomotor activity. *Endocrinology* **142**, 5220-5.

**Messerli, F. H., Bangalore, S., Bavishi, C. and Rimoldi, S. F.** (2018). Angiotensin-Converting Enzyme Inhibitors in Hypertension: To Use or Not to Use? *J Am Coll Cardiol* **71**, 1474-1482.

**Millet, J. K. and Whittaker, G. R.** (2015). Host cell proteases: Critical determinants of coronavirus tropism and pathogenesis. *Virus Res* **202**, 120-34.

**Nakatani, Y. and McLysaght, A.** (2017). Genomes as documents of evolutionary history: a probabilistic macrosynteny model for the reconstruction of ancestral genomes. *Bioinformatics* **33**, i369-i378.

**Natesh, R., Schwager, S. L., Evans, H. R., Sturrock, E. D. and Acharya, K. R.** (2004). Structural details on the binding of antihypertensive drugs captopril and enalaprilat to human testicular angiotensin I-converting enzyme. *Biochemistry* **43**, 8718-24.

**Rice, G. I., Thomas, D. A., Grant, P. J., Turner, A. J. and Hooper, N. M.** (2004). Evaluation of angiotensin-converting enzyme (ACE), its homologue ACE2 and neprilysin in angiotensin peptide metabolism. *Biochem J* **383**, 45-51.

**Richardson, S., Hirsch, J. S., Narasimhan, M., Crawford, J. M., McGinn, T., Davidson, K. W., and the Northwell, C.-R. C., Barnaby, D. P., Becker, L. B., Chelico, J. D. et al.** (2020). Presenting Characteristics, Comorbidities, and Outcomes Among 5700 Patients Hospitalized With COVID-19 in the New York City Area. *JAMA*.

**Roca-Ho, H., Riera, M., Palau, V., Pascual, J. and Soler, M. J.** (2017). Characterization of ACE and ACE2 Expression within Different Organs of the NOD Mouse. *Int J Mol Sci* **18**.

**Sato, T., Suzuki, T., Watanabe, H., Kadowaki, A., Fukamizu, A., Liu, P. P., Kimura, A., Ito, H., Penninger, J. M., Imai, Y. et al.** (2013). Apelin is a positive regulator of ACE2 in failing hearts. *J Clin Invest* **123**, 5203-11.

**Sommerstein, R., Kochen, M. M., Messerli, F. H. and Grani, C.** (2020). Coronavirus Disease 2019 (COVID-19): Do Angiotensin-Converting Enzyme Inhibitors/Angiotensin Receptor Blockers Have a Biphasic Effect? *J Am Heart Assoc* **9**, e016509.

**Szokodi, I., Tavi, P., Foldes, G., Voutilainen-Myllyla, S., Ilves, M., Tokola, H., Pikkarainen, S., Piuhola, J., Rysa, J., Toth, M. et al.** (2002). Apelin, the novel endogenous ligand of the orphan receptor APJ, regulates cardiac contractility. *Circ Res* **91**, 434-40.

**Uri, K., Fagyas, M., Kertesz, A., Borbely, A., Jenei, C., Bene, O., Csanadi, Z., Paulus, W. J., Edes, I., Papp, Z. et al.** (2016). Circulating ACE2 activity correlates with cardiovascular disease development. *J Renin Angiotensin Aldosterone Syst* **17**.

**Uri, K., Fagyas, M., Manyine Siket, I., Kertesz, A., Csanadi, Z., Sandorfi, G., Clemens, M., Fedor, R., Papp, Z., Edes, I. et al.** (2014). New perspectives in the renin-angiotensin-aldosterone system (RAAS) IV: circulating ACE2 as a biomarker of systolic dysfunction in human hypertension and heart failure. *PLoS One* **9**, e87845.

**Vaduganathan, M., Vardeny, O., Michel, T., McMurray, J. J. V., Pfeffer, M. A. and Solomon, S. D.** (2020). Renin-Angiotensin-Aldosterone System Inhibitors in Patients with Covid-19. *N Engl J Med* **382**, 1653-1659.

**Vuille-dit-Bille, R. N., Camargo, S. M., Emmenegger, L., Sasse, T., Kummer, E., Jando, J., Hamie, Q. M., Meier, C. F., Hunziker, S., Forras-Kaufmann, Z. et al.** (2015). Human intestine luminal ACE2 and amino acid transporter expression increased by ACE-inhibitors. *Amino Acids* **47**, 693-705.

**Walls, A. C., Park, Y. J., Tortorici, M. A., Wall, A., McGuire, A. T. and Veesler, D.** (2020). Structure, Function, and Antigenicity of the SARS-CoV-2 Spike Glycoprotein. *Cell* **181**, 281-292 e6.

**Walters, T. E., Kalman, J. M., Patel, S. K., Mearns, M., Velkoska, E. and Burrell, L. M.** (2017). Angiotensin converting enzyme 2 activity and human atrial fibrillation: increased plasma angiotensin converting enzyme 2 activity is associated with atrial fibrillation and more advanced left atrial structural remodelling. *Europace* **19**, 1280-1287.

**Wang, B., Li, R., Lu, Z. and Huang, Y.** (2020). Does comorbidity increase the risk of patients with COVID-19: evidence from meta-analysis. *Aging (Albany NY)* **12**.

**Yan, R., Zhang, Y., Li, Y., Xia, L., Guo, Y. and Zhou, Q.** (2020). Structural basis for the recognition of SARS-CoV-2 by full-length human ACE2. *Science* **367**, 1444-1448.

**Yang, P., Kuc, R. E., Brame, A. L., Dyson, A., Singer, M., Glen, R. C., Cheriyan, J., Wilkinson, I. B., Davenport, A. P. and Maguire, J. J.** (2017). [Pyr(1)]Apelin-13(1-12) Is a Biologically Active ACE2 Metabolite of the Endogenous Cardiovascular Peptide [Pyr(1)]Apelin-13. *Front Neurosci* **11**, 92.

**Yvan-Charvet, L., Even, P., Bloch-Faure, M., Guerre-Millo, M., Moustaid-Moussa, N., Ferre, P. and Quignard-Boulange, A.** (2005). Deletion of the angiotensin type 2 receptor (AT2R) reduces adipose cell size and protects from diet-induced obesity and insulin resistance. *Diabetes* **54**, 991-9.
